# In-depth mapping of the mouse brain N-glycoproteome reveals widespread N-glycosylation of diverse brain proteins

**DOI:** 10.18632/oncotarget.9737

**Published:** 2016-05-31

**Authors:** Pan Fang, Xin-jian Wang, Yu Xue, Ming-qi Liu, Wen-feng Zeng, Yang Zhang, Lei Zhang, Xing Gao, Guo-quan Yan, Jun Yao, Hua-li Shen, Peng-yuan Yang

**Affiliations:** ^1^ Minhang Hospital and Institutes of Biomedical Sciences, Fudan University, Shanghai, China; ^2^ State Key Laboratory of Medical Neurobiology, Institutes of Brain Science, and Collaborative Innovation Center for Brain Science, Fudan University, Shanghai, China; ^3^ Department of Chemistry, Fudan University, Shanghai, China; ^4^ Key Laboratory of Intelligent Information Processing of Chinese Academy of Sciences (CAS), Institute of Computing Technology, CAS, Beijing, China; ^5^ Department of Systems Biology for Medicine and School of Basic Medical Sciences, Fudan University, Shanghai, China

**Keywords:** N-glycoproteomics, mouse brain, mass spectrometry, brain physiological activities, disease biomarker

## Abstract

N-glycosylation is one of the most prominent and abundant posttranslational modifications of proteins. It is estimated that over 50% of mammalian proteins undergo glycosylation. However, the analysis of N-glycoproteins has been limited by the available analytical technology. In this study, we comprehensively mapped the N-glycosylation sites in the mouse brain proteome by combining complementary methods, which included seven protease treatments, four enrichment techniques and two fractionation strategies. Altogether, 13492 N-glycopeptides containing 8386 N-glycosylation sites on 3982 proteins were identified. After evaluating the performance of the above methods, we proposed a simple and efficient workflow for large-scale N-glycosylation site mapping. The optimized workflow yielded 80% of the initially identified N-glycosylation sites with considerably less effort. Analysis of the identified N-glycoproteins revealed that many of the mouse brain proteins are N-glycosylated, including those proteins in critical pathways for nervous system development and neurological disease. Additionally, several important biomarkers of various diseases were found to be N-glycosylated. These data confirm that N-glycosylation is important in both physiological and pathological processes in the brain, and provide useful details about numerous N-glycosylation sites in brain proteins.

## INTRODUCTION

N-glycosylation is one of the most prominent posttranslational modifications of proteins, and is a key component of many key biological processes, including cell adhesion, molecular trafficking and clearance, receptor activation, signal transduction, and endocytosis [1]. Aberrant glycosylation is associated with a number of diseases, including neurodegenerative disorders and cancer [2, 3]. Many key proteins involved in disease progression are N-glycosylated, such as amyloid precursor protein (APP) and β-site amyloid precursor protein-cleaving enzyme 1 (BACE-1) in Alzheimer's disease (AD) and alpha-fetoprotein (a clinical biomarker) in hepatocellular carcinoma [4, 5]. Glycan-based therapies have gained increasing attention in the medical field as new tools and techniques have been developed [6]. Given the biological importance of N-glycosylation, the N-glycoproteomes of various tissues, cell lines or serum/plasma samples from humans or model animals have been extensively investigated in the past decade [7–12]. However, the extent of the N-glycoproteome—a prerequisite for a detailed understanding of its function—has not yet been thoroughly uncovered, due to the technological limitations of N-glycoproteomics.

Although it is estimated that more than 50% of mammalian proteins undergo glycosylation, glycopeptides are usually relatively low-abundance (2% to 5%) compared with non-glycosylated peptides [13, 14]. Furthermore, non-glycosylated species reduce the detection sensitivity of mass spectrometry (MS) analysis for glycopeptides by greatly diminishing their ionization efficiency. The microheterogeneity of glycans further reduces the relative amount of each glycoform. The enrichment of N-glycopeptides from complex biological samples is usually an essential step in their analysis, since N-glycopeptides may account for as little as 0.5% of the identified peptides without enrichment [7]. Many enrichment techniques have been developed in recent years, such as hydrophilic interaction liquid chromatography (HILIC), zwitterionic chromatography−hydrophilic interaction chromatography (ZIC-HILIC), lectin affinity chromatography, hydrazide chemistry, titanium dioxide (TiO_2_) chromatography, and so on [15]. However, the specificity and coverage of these enrichment techniques for glycopeptides have generally been insufficient. It is usually difficult to enrich all the glycopeptides in a complex sample with a single enrichment technique. In addition, glycans may impede the cleavage activity of trypsin at lysine or arginine when these residues are adjacent to the glycosite. A multi-protease strategy is an alternative to trypsin digestion in N-glycoproteomics [16]. Due to the high complexity and microheterogeneity of glycopeptides, offline fractionation strategies before or after enrichment are often adopted to enhance the separation of glycopeptides before routine reversed-phase liquid chromatography tandem mass spectrometry (RPLC-MS/MS) analysis [17, 18]. The diverse fractionation approaches used in proteomics, such as strong cation exchange chromatography (SCX), high-pH reversed-phase chromatography (bRP) and peptide immobilized pH gradient isoelectric focusing (IPG-IEF) [18–20], are also applicable to glycoproteomics.

The combination of different methods has been effective for in-depth mapping of the N-glycoproteome. Zielinska *et al.* developed a “filter aided sample preparation” (FASP)-based method, in which glycopeptides were enriched by their binding to a single or multiple lectin(s) on the top of a filter. In this manner, the authors mapped 6367 N-glycosylation sites on 2352 proteins in four mouse tissues and blood plasma [7]. More recently, Zhu *et al.* comprehensively mapped the N-glycosylation of human liver proteins by combining the enrichment techniques of hydrazide chemistry and click maltose−hydrophilic interaction chromatography, performing multi-enzyme digestion, and using two different types of mass spectrometers [12]. They identified 2210 N-glycoproteins and 4783 N-glycosylation sites, comprising the largest dataset of the human N-glycoproteome.

Despite the progress in profiling the N-glycoproteomes of biological samples, the available methods are varied, including multiple proteases, different enrichment techniques, diverse fractionation strategies and even various mass spectrometers. The combination of different methods increases the number of identified N-glycoproteins, but also greatly increases the required sample amounts, workloads, and measurement times on instruments. It is difficult for researchers to choose the appropriate method or combination of methods for large-scale characterization of the N-glycoproteome when they want to establish methods and evaluate a new biological system. For this reason, we not only combined multiple methods, including seven protease treatments, four enrichment techniques and two fractionation strategies for in-depth mapping of the mouse brain N-glycoproteome, but also evaluated the complementarity of different methods in the global analysis of the N-glycoproteome. Overall, we identified 13492 N-glycopeptides containing the N-!P-[S|T|C] sequence motif (where !P is not proline) in the mouse brain, corresponding to 8386 N-glycosylation sites on 3982 N-glycoproteins. Furthermore, we proposed a simple and efficient workflow, which only involved three protease treatments, two enrichment techniques and 1D RPLC-MS/MS, and which required about one-third of the measurement time of the combination of all the methods in this study. The optimized workflow yielded about 80% of the initially identified N-glycosylation sites with considerably less effort. As far as we know, ours is the largest N-glycoproteome dataset from a single mammalian tissue to date. Our results revealed the ubiquity of N-glycosylation in the mouse brain proteome.

## RESULTS

### Overview

**Figure 1:**
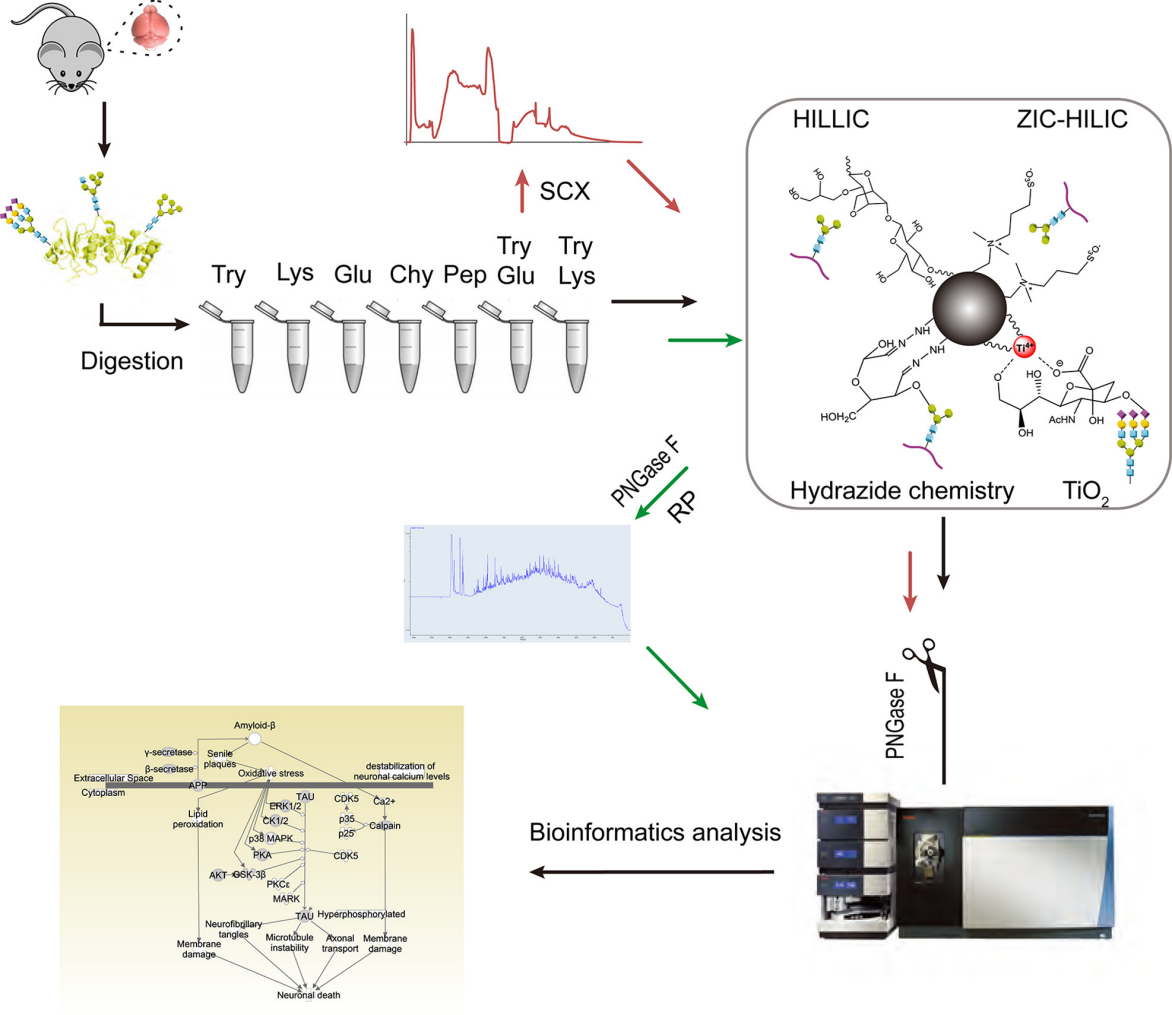
Experimental design for in-depth mapping of the N-glycoproteome in the mouse brain Mouse brain proteins were digested with seven sets of proteases. The efficiencies of the proteases were compared while ZIC-HILIC was used as the enrichment technique (black arrow). Then, four enrichment techniques were evaluated on tryptic peptides (black arrow). Finally, the influence of fractionation before (with SCX) or after (with bRP) enrichment with ZIC-HILIC was assessed. For all of the above experiments, N-glycans were detached with PNGase F before online RPLC-MS/MS detection.

We evaluated different methods for three different stages of N-glycoproteomic analysis: protease treatment, enrichment and fractionation. The experimental design of this study is depicted in Figure 1. Mouse brain proteins were first digested with seven sets of proteases, comprising three high-specificity proteases (trypsin, Glu-C and Lys-C), two low-specificity proteases (chymotrypsin and pepsin) and two protease combinations (trypsin coupled with Lys-C (TryLys) and trypsin coupled with Glu-C (TryGlu)). The detailed information about each type of protease is listed in Supplementary Table S1. Afterwards, four types of enrichment techniques with distinct mechanisms were compared, including hydrophilic interaction liquid chromatography (HILIC, Sepharose CL-4B media), zwitterionic chromatography−hydrophilic interaction liquid chromatography (ZIC-HILIC), hydrazide chemistry, and TiO_2_ chromatography. Furthermore, we examined two fractionation strategies: fractionation with strong cation exchange chromatography (SCX) before enrichment, and fractionation with bRP after enrichment. Finally, we performed bioinformatics analyses on all the identified N-glycoproteins.

### Improved identification of N-glycosylation sites through treatment with multiple proteases

**Figure 2:**
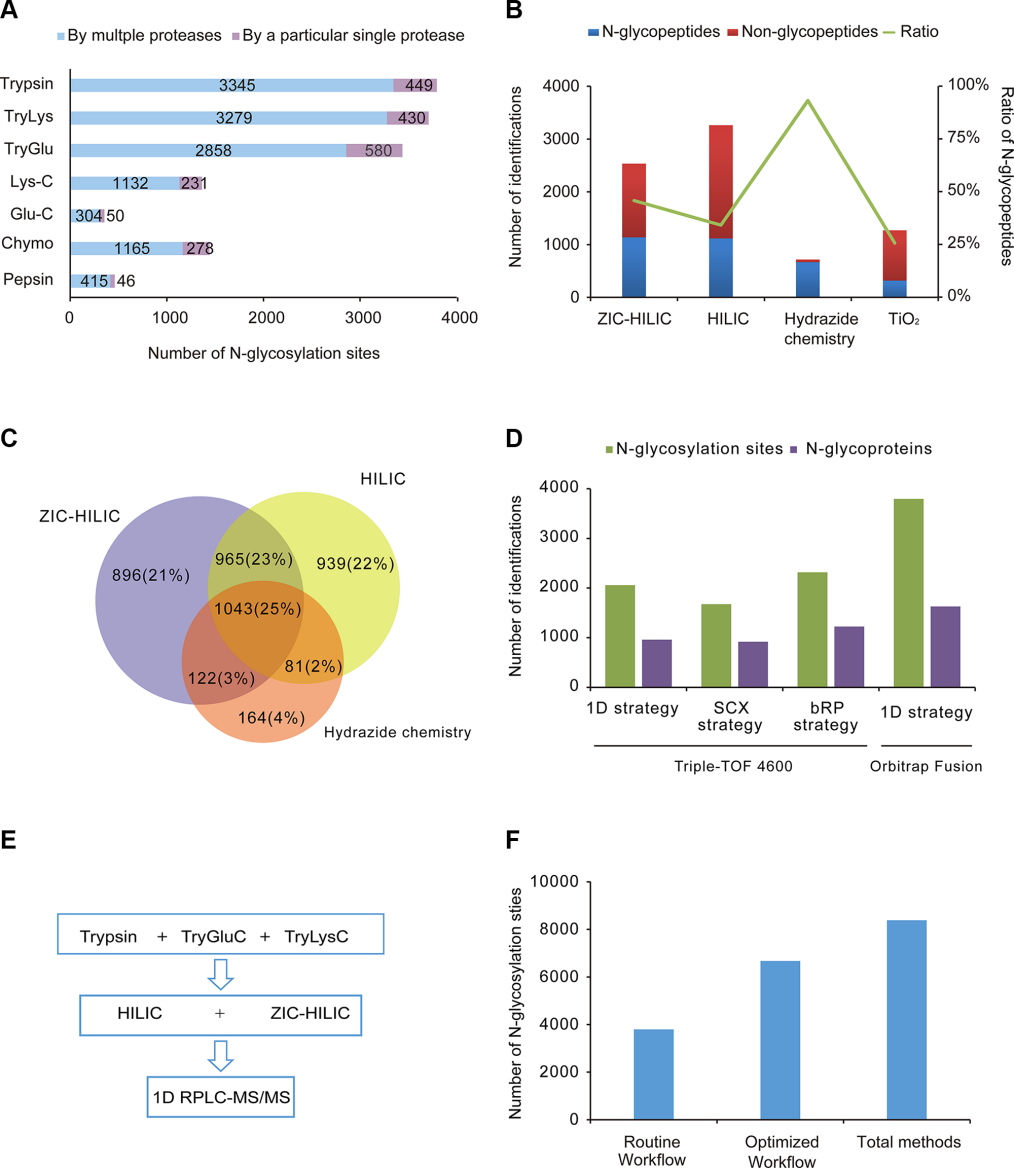
Evaluation of different methods and an optimal workflow (**A**) Comparison of seven sets of proteases. The number of N-glycosylation sites identified with trypsin, trypsin coupled with Lys-C, trypsin coupled with Glu-C, Lys-C, Glu-C, chymotrypsin and pepsin are shown. Purple: sites only detected with a particular protease; Blue: sites also detected with other proteases. (**B**) Comparison of the efficiencies and specificities of the four different enrichment techniques (ZIC-HILIC, Sepharose CL-4B, hydrazide chemistry and TiO2 chromatography). The average numbers of N-glycopeptides and non-N-glycopeptides are shown, as well as the average percentages of identified N-glycopeptides among the total identified peptides, from triplicate experiments. (**C**) Overlap of N-glycosylation sites identified by the three unbiased enrichment techniques (ZIC-HILIC, Sepharose CL-4B and hydrazide chemistry). (**D**) Comparison of different fractionation strategies. The number of N-glycosylation sites and N-glycoproteins identified following different fractionation strategies (1D strategy, SCX strategy and bRP strategy on Triple-TOF 4600; 1D strategy on Orbitrap Fusion) are shown. (**E**) The optimal workflow proposed in this study. (**F**) Comparison of the routine workflow, optimal workflow and combination of all methods.

Equal amounts of proteins from mouse brain were separately digested with the seven sets of proteases. After parallel enrichment by ZIC-HILIC, the N-glycopeptides were treated with PNGase F and analyzed by RPLC-MS/MS in triplicate. In the seven sets of experiments, 5790 N-glycosylation sites on 2451 N-glycoproteins were identified. As for the particular sets of proteases, each yielded an exclusive set of N-glycosylation sites, as well as sites overlapping with those produced by the other sets (Figure 2A). These exclusive sites made up a large portion (40.6%) of the identified N-glycosylation sites. The combined use of multiple proteases increased the number of identified N-glycosylation sites by 73.1% compared with the use of trypsin alone. Meanwhile, 59.4% of the N-glycosylation sites were identified by more than one type of enzyme group, which increased our identification confidence in these sites. Notably, the number of N-glycosylation sites detected with trypsin, TryLys and TryGlu was much larger than the number detected with other proteases (3400–3800 vs. 350–1400). The three protease sets including trypsin covered 88.3% of the N-glycosylation sites identified by the proteases, 34.8% more than trypsin alone. Considering the cumbersomeness of using seven sets of proteases for protein digestion, the combination of trypsin, TryGlu and TryLys would be effective and practical for large-scale N-glycoproteomics.

### Enhanced identification of N-glycosylation sites with complementary enrichment techniques

Glycopeptides digested with trypsin were enriched by four techniques in parallel and then analyzed by RPLC-MS/MS in triplicate. The enrichment efficiency (the number of identified N-glycopeptides) and the specificity (the ratio of the identified N-glycopeptides to the total identified peptides) of each of the four techniques was evaluated. As shown in Figure 2B, HILIC and ZIC-HILIC resulted in comparable numbers of N-glycopeptides (1118 and 1138, respectively); however, the enrichment specificity of ZIC-HILIC (46%) was higher than HILIC (34%). Hydrazide chemistry had the highest enrichment specificity (93%) of the four enrichment techniques, but identified a relatively low number of N-glycopeptides (667). As for TiO_2_ chromatography, which specifically attracted sialic acid-containing glycopeptides, this enrichment yielded 318 sialoglycopeptides with a specificity of 26%.

As TiO_2_ chromatography identified the lowest number of glycopeptides, the three unbiased enrichment techniques (HILIC, ZIC-HILIC and hydrazide chemistry) were selected for further investigation of complementarity (Figure 2C). We also performed bRP fractionation after using the three enrichment techniques, and combined the results from the non-fractionation and bRP fractionation strategies. Thus, in total, 3028, 3026 and 1410 N-glycosylation sites were identified by HILIC, ZIC-HILIC and hydrazide chemistry, respectively. Each enrichment technique contributed a substantial number of exclusive N-glycosylation sites. The overlap among the three enrichment techniques was 25%. Thus, the combination of different enrichment techniques increased the N-glycoproteome coverage by increasing the enrichment complementarity.

The limitation of N-glycoproteomic experiments that use PNGase F is the reliance on the presence of Asn deamidation as a diagnostic for N-linked glycan site assignment. Deamidation of Asn to Asp may occur during sample storage or processing [9]. Therefore, we performed an additional control to determine the false positive assignment of N-glycoslation sites [21]. In brief, enriched peptides were divided into two equal parts, one of which was treated with PNGase F and the other of which was not. The two parts were both analyzed by LC-MS/MS. Asn deamidation was set as a variable modification in database searches. Potential false positives were assigned to the samples without PNGase F only if the deamidated Asn was found to be part of the N-linked sequon (N-!P-[S|T|C]). In this way, many experimental false positives could be detected; The false-positive rates detected for HILIC, ZIC-HILIC, hydrazide chemistry and TiO_2_ chromatography were 3.9%, 1.7%, 0.7% and 7.1%, respectively (Supplementary Table S2). The potential false positives are described in Supplementary Table S3. The false-positive rates correlated closely with the enrichment specificities. As for ZIC-HILIC and hydrazide chemistry, which had very high specificities, few non-glycopeptides containing Asn residues were present in the samples, and even fewer contained the N-!P-[S|T|C] motif. Consequently, considering the efficiencies, specificities and false-positive rates of the four enrichment techniques, we suggest HILIC and ZIC-HILIC as the optimal enrichment techniques for large-scale N-glycosylation site mapping.

### The efficiency of fractionation strategy in N-glycoproteomics

Pre-fractionation is often added to the proteomic workflow so that proteins in complex samples can be mapped in-depth. Diverse fractionation strategies in proteomics were also applied to N-glycoproteomics. In this study, we mainly compared two kinds of pre-fractionation strategies (fractionation with SCX before ZIC-HILIC enrichment (the SCX strategy) and fractionation with bRP after ZIC-HILIC enrichment (the bRP strategy)) with non-fractionation method (the 1D strategy). Compared with the 1D strategy, the number of identified N-glycosylation sites increased by 12.6% with the bRP strategy (2316), while it decreased by 23% with the SCX strategy (1675) (Figure 2D). The bRP strategy did not greatly increase the number of identified N-glycosylation sites, suggesting that the 1D strategy is sufficient for the separation of the enriched N-glycopeptides. Furthermore, the 1D strategy was more efficient than the SCX strategy. When the 1D strategy was applied on a more sensitive mass spectrometer (Orbitrap fusion), the number of identified N-glycosylation sites increased significantly to 3794, 1.8-fold the number identified by the 1D strategy and 1.6-fold the number identified by the bRP strategy on the Triple-TOF 4600 system. Considering its simplicity, practicality and efficiency, the 1D strategy is more applicable than the other strategies for mapping the N-glycoproteome.

### Determination of the optimal workflow for in-depth N-glycoproteome mapping

By combining all the methods in this study, we identified a total of 13492 N-glycopeptides, corresponding to 8386 N-glycosylation sites on 3982 proteins in the mouse brain (Supplementary Table S4). After evaluating the performances of the proteases, enrichment techniques and fractionation strategies, we proposed a simple workflow for in-depth characterization of the N-glycoproteome (Figure 2E). Considering their efficiency and simplicity, we selected trypsin, TryLys and TryGlu for protein digestion, HILIC and ZIC-HILIC for the glycopeptide enrichment, and 1D-RPLC-MS/MS for N-glycopeptide detection. The optimized workflow was able to cover 80% of the initially identified N-glycosylation sites, detecting 6673 N-glycosylation sites on 3258 proteins (Figure 2F). Furthermore, the number of N-glycosylation sites was 76% greater with the proposed workflow than with the routine workflow (trypsin, ZIC-HILIC, and 1D-RPLC/MS/MS). We believe that this strategy will be a useful and general approach for comprehensive N-glycoproteome profiling in other complex biological samples.

### Characterization of N-glycoproteins in the mouse brain

**Figure 3:**
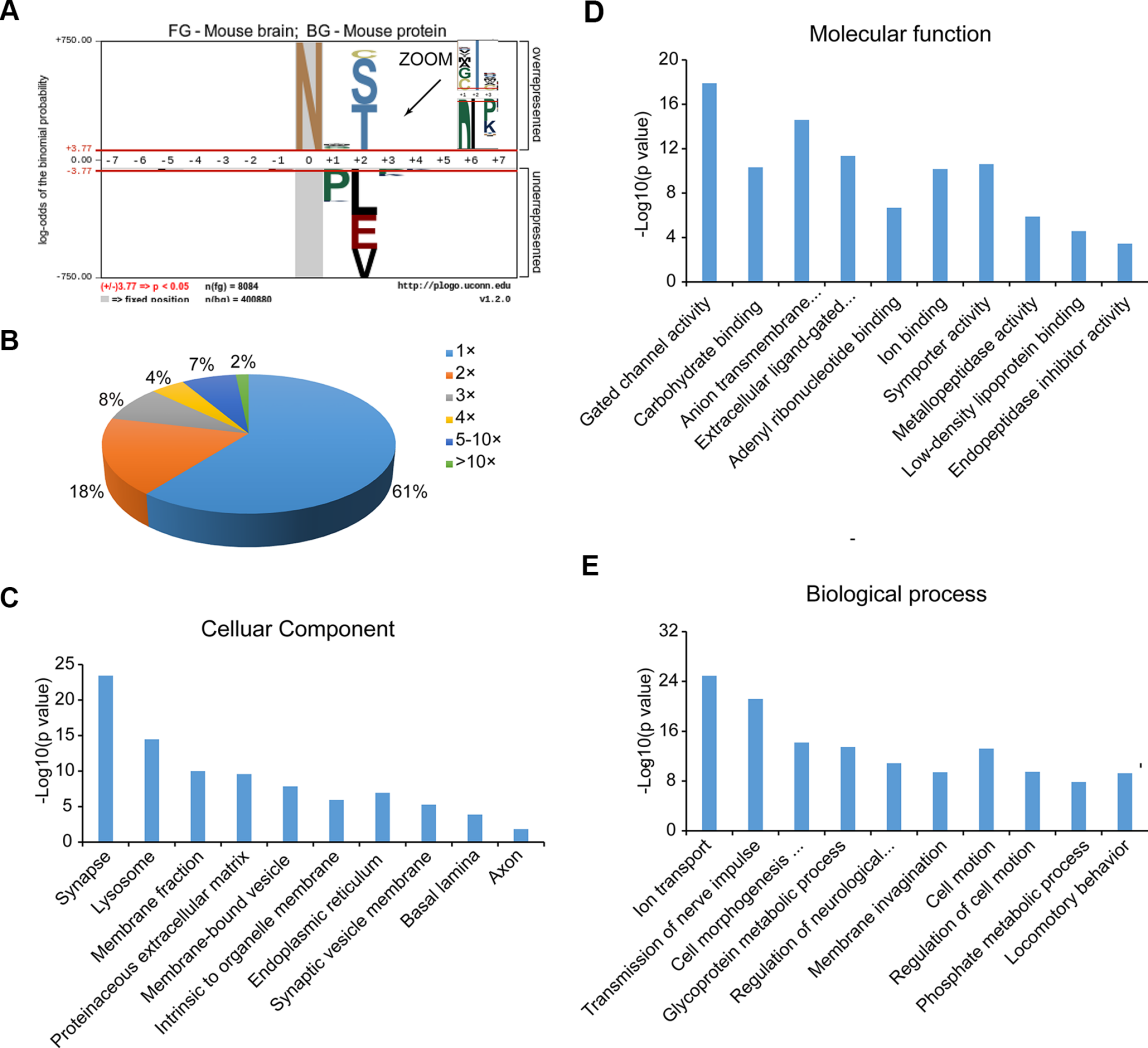
Characterization of N-glycoproteins (**A**) N-glycosylation consensus sequence as derived using pLogo. (**B**) Distribution of singly and multiply glycosylated proteins. (**C**–**E**) Cellular components (C), molecular functions (D) and biological processes (E) that are enriched in the N-glycoproteome compared to the entire mouse proteome according to Gene Ontology analysis.

The canonical N-linked glycosylation motif is N-!P-[S|T|C] (where !P is not proline). We reasoned that our large-scale dataset would be useful for testing the generality of this motif. We used a probabilistic approach, pLogo, to visualize the sequence motifs of the 8386 identified N-glycosylation sites, 8084 of which were valid for pLogo generation [22]. Position-specific amino acids around the glycosylated modification sites (seven amino acids on each side) were scaled relative to their statistical significance in the context of mouse proteomic backgrounds. As shown in Figure 3A, proline (P) was significantly underrepresented (below the x axis), not only at the + 1 position, but also at the + 3 position (zoom in Figure 3A), while serine (S), threonine (T) and cysteine (C) were significantly overrepresented at the + 2 position. Threonine (48%) and serine (46%) occurred more frequently than cysteine (6%). A preference for cysteine, glycine and alanine at the + 1 position was also obvious.

Of the 3982 N-glycoproteins, approximately 60% carried a single N-linked sugar chain (Figure 3B). The percentages of N-glycoproteins containing two or three N-glycosylation sites were 18% and 8%, respectively, and the average degree of N-glycosylation was 2.1. There were 342 proteins that contained more than five N-glycosylation sites, and 65 with at least 10 sites. Prolow-density lipoprotein receptor-related protein 1 (LRP1) and low-density lipoprotein receptor related protein 1B (LRP1B) were the most heavily N-glycosylated proteins, with 56 and 44 N-glycosylation sites, respectively.

To obtain an overview of the primary subcellular compartments, molecular functions and biological processes of N-linked glycoproteins, we used the DAVID tool (http://david.abcc.ncifcrf.gov/) to perform Gene Ontology function enrichment analysis on all the identified N-glycoproteins [23]. The top enriched clusters among the subcellular compartments were the membrane, extracellular region, endoplasmic reticulum, vesicles and lysosomes, which are involved in the processes of N-glycoprotein synthesis and transport. In addition, organelles specific to brain tissue were also significantly enriched, such as synapses, synaptic vesicle membranes, basal lamina, and axons (Figure 3C). Many molecular functions that are known to be performed by N-glycoproteins were enriched; as shown in Figure 3D, the top five clusters were gated channel activity, carbohydrate binding, anion transmembrane transporter activity, extracellular ligand-gated ion channel activity, and adenyl ribonucleotide binding. The major overrepresented biological processes included ion transport, nerve impulse transmission, cell morphogenesis involved in differentiation, glycoprotein metabolic processes, and neurological system regulation (Figure 3E). For detailed information on the top ten enriched clusters, see Supplementary Table S5.

### Analysis of pathways involving N-glycosylation

**Figure 4:**
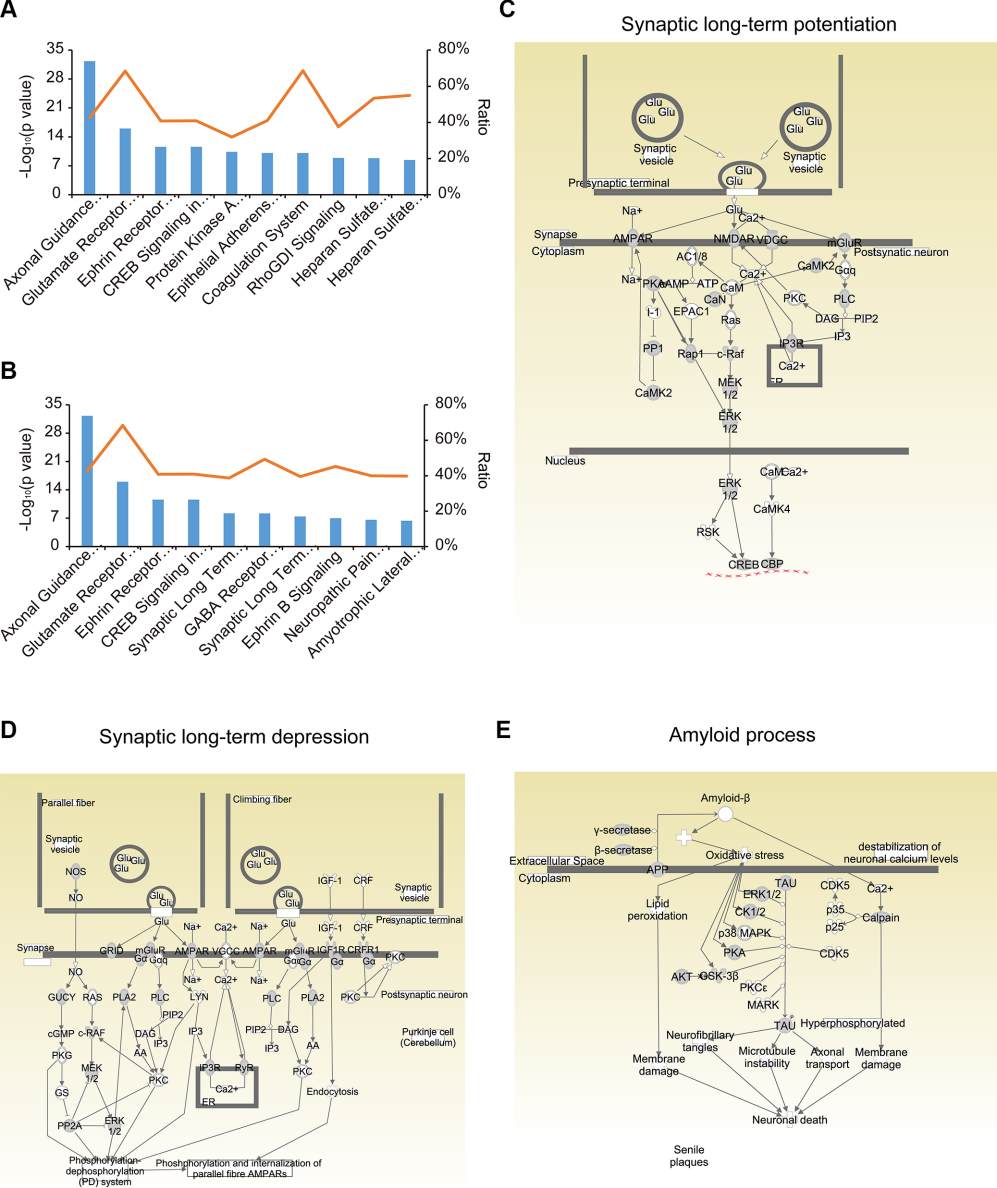
Pathway analysis of the N-glycoproteome using IPA (**A**) The top 15 canonical pathways in IPA. (**B**) The pathways involving neurotransmitters and the nervous system among the canonical pathways. (**C**, **D**) Signaling events in the synaptic long-term potentiation (C) and depression (D) pathways. (**E**) Signaling events in amyloid processing.

Ingenuity Pathway Analysis (IPA) (http://www.ingenuity.com) revealed that the identified N-glycoproteins were significantly associated with 188 canonical pathways (*p* < 0.05) (Supplementary Table S6). The top four pathways are of great importance in nervous system signaling: axonal guidance signaling, glutamate receptor signaling, ephrin receptor signaling, and CREB signaling in neurons (Figure 4A). In addition, there were 27 significant pathways involving neurotransmitters and the nervous system-for instance, synaptic long-term potentiation (LTP), GABA receptor signaling, synaptic long-term depression (LTD), and so on (Figure 4B and Supplementary Table S7). Other key biological pathways were also enriched, including protein kinase A signaling and epithelial adherent junction signaling.

It is widely believed that long-lasting, activity-dependent changes in synaptic strength, including LTP and LTD, could be the molecular and cellular basis of experience-dependent plasticities, such as learning and memory [24]. In this study, we found that proteins in both LTP and LTD pathways were extensively N-glycosylated; about 40% of the molecules (47/119) in LTP pathways and 39% (55/142) in LTD pathways were N-glycosylated (Figure 4C and 4D, Supplementary Tables S8 and S9). In the LTP pathway, the primary glutamate receptors (GluRs) on the postsynaptic membrane were N-glycosylated, including the α-amino-3-hydroxy-5-methylisoxazole-4-propionic acid receptor (AMPAR), the N-methyl-D-aspartate receptor (NMDAR), and the metabotropic glutamate receptor (mGluR). In the LTD pathway, receptors on the plasma membrane, including AMPAR, mGluR, glutamate receptor delta (GRID), insulin-like growth factor 1 receptor (IGF1R), and corticotropin releasing hormone receptor 1 (CRFR1) were N-glycosylated. These N-glycosylated proteins are crucial for LTP and LTD [25, 26], and inhibition or loss of N-glycosylation greatly hinders LTP [27, 28]. The underlying molecular relationship between N-glycosylation and plasticity has not been explored, but may reveal the causal link between these synaptic processes and memory, which has thus far been difficult to demonstrate [29]. In addition, 24 glutamate receptors were found to be N-glycosylated in our dataset, corresponding to 129 N-glycosylation sites (Supplementary Table S10). Such detailed N-glycosylation site information may be useful for understanding the role of these glutamate receptors in the central nervous system.

Amyloid processing is the major process that contributes to AD [30]. The two core pathological hallmarks of AD are amyloid plaques composed of amyloid β-peptide (Aβ) and neurofibrillary tangles composed of hyperphosphorylated microtubule-associated protein tau [31]. There is much evidence that alterations in glycosylation patterns influence the pathogenesis and progression of AD [32–35]. APP is processed into Aβ through β-secretase and γ-secretase, while the deposition of Aβ in the brain triggers neuronal dysfunction and death. N-glycosylation of APP and its secretases is an important component of the pathological changes of AD. For instance, inhibition of the complex N-glycosylation or sialylation of APP disrupts the sorting of axons and the secretion of APP, sAPPα, sAPPβ and Aβ [4]. Lack of bisecting GlcNAc on β-secretase reduced Aβ plaque formation and improved cognitive function in knockout mice for the biosynthetic enzyme of bisecting GlcNAc [36]. Despite all this evidence, the vast number of potential glycosylation sites has made the field of glycobiology difficult. In this study, 31% (16/51) of the identified proteins involved in amyloid processing were N-glycosylated (Figure 4E and Supplementary Table S11).

### Disease-related biomarkers and N-glycoproteins

Biomarker filter analysis by IPA revealed that 478 of the 3982 identified N-glycoproteins were related to human diseases, including neurological disease (371), cancer, and developmental disorders (Supplementary Table S12). Biomarkers for AD diagnosis including APP and BACE-1 [37] were among these N-glycosylated proteins. Studying the N-glycosylation of these proteins might improve their sensitivity and specificity as diagnostic biomarkers.

Besides these disease-related biomarkers, numerous crucial proteins involved in various diseases were also N-glycosylated, including 217 AD-related proteins (Supplementary Table S13), 195 schizophrenia-related proteins, and 150 epilepsy-related proteins. Altered N-glycosylation has already been demonstrated in the above-mentioned diseases [38, 39]. For instance, the N-glycosylation patterns of Reelin and acetylcholinesterase were found to be changed in AD, as were those of the GABAA receptor subunits in schizophrenia [33, 34]. However, the precise consequences of N-glycosylation in these diseases remain to be explored.

## DISCUSSION

Limited by the current analytical technologies, the N-glycoproteomics is far behind other PTMs, such as phosphoproteomics. However, the available methods in glycoproteomics are varied. It is urgent to set up highly efficient and particle methods for in-depth mapping N-glycoproteomes. In this study, we performed a systematic comparison of several critically important steps including enzymatic digestion, enrichment as well as fractionation in the global analysis of protein N-glycosylation. We also deployed the optimized procedure in the analysis of the N-glycosylated proteome in mouse brain.

Trypsin is the most widely used protease in bottom-up proteomics, but its cleavage activity may be blocked by the carbohydrates attached to glycoproteins, resulting in incomplete digestion of N-glycoproteins and long peptides which are not suitable for MS detection [16]. In addition, it is rarely possible to identify tryptic peptides with N-terminal glycosylated asparagine (Asn) residues, due to the poor deglycosylation capacity of Peptide-N-Glycosidase F (PNGase F) [40]. To improve the efficiency of N-glycosylation site identification, we tested several proteases for their applicability to the N-glycoproteomic study. Our results demonstrated that multiple proteases improved the identification of N-glycosylation sites compared to trypsin alone. The two sets of dual-protease treatment (TryLys and TryGlu) identified comparable glycopeptides with trypsin and much more than other single protease. The addition of another protease to trypsin, such as Lys-C or Glu-C, could allow large post-trypsin peptides to be digested for a second time. Based on the molecular weight distribution of the identified glycopeptides (Supplementary Figure S1), it was evident that TryGlu produced more glycopeptides within the range of molecular masses suitable for MS detection. Furthermore, the ratios of both one and multiple missed cleavages of glycopeptides by TryLys or TryGlu were both lower than that of trypsin alone (Supplementary Figure S2), which indicated the improved digesting efficiency by using a dual-protease method compared to trypsin alone. The combination of trypsin with the two sets of dual-protease method (TryGlu and TryLys) was effective and particle for high throughput identification of N-glycosylation sites.

The enrichment of N-glycopeptides from a complicated peptide mixture is quite important for the successful identification of glycoproteins and glycopeptides. We assessed the performance of four enrichment techniques with distinct mechanisms (Figure 1): HILIC (in which hydrogen bonds form between glycopeptides and the polar stationary phase), ZIC-HILIC (in which polar interactions and ionic interactions occur between glycopeptides and the stationary phase), hydrazide chemistry (in which covalent hydrazine bonds form between aldehydes on the oxidized glycan and hydrazide groups on the surface of the support medium) and TiO_2_ chromatography (in which multivalent complexes form between TiO_2_ and sialoglycopeptides) [15]. We compared the four methods in detail including the efficiency, specificity, complementarity as well as false positive rates. These information are useful for researchers to select enrichment methods for different aims. Considering the comprehensive influence, HILIC and ZIC-HILIC were more outstanding in large scale N-glycosylation site mapping.

Pre-fractionation is often added to the proteomic workflow so that proteins in complex samples can be mapped in-depth. Diverse fractionation methods were also used in N-glycoproteomics for in-depth mapping of N-glycosylation sites. In this study, we compared two fractionation strategies with non-fractionation method in N-glycoproteomics: fractionation with SCX before enrichment and fractionation with bRP after enrichment. Our results showed that pre-fractionation slightly increased the yield of N-glycosylation sites compared with one dimensional separation (the 1D strategy) but required more starting materials, more cumbersome procedures and longer measurement times. The efficiency of the 1D strategy was even better than the SCX strategy despite its ability of concentrating glycopeptides. This could be because that pre-fractionation before enrichment requires more steps, thus causing loss of samples. So the 1D strategy is simple and efficient for mapping the N-glycoproteome.

By comparing different methods in the three stages of N-glycoproteomic analysis, we determined their respective merits and weaknesses, which will be useful information for the design of future experiments. In total, 13492 N-glycopeptides containing 8386 N-glycosylation sites on 3982 proteins from the mouse brain were identified. Although the combination of multiple methods greatly improved the number of N-glycosylation sites identified, considerably more effort was required to generate a large-scale dataset. Therefore, we proposed a simple and highly efficient workflow consisting of three protease treatments (trypsin, TryLys and TryGlu), two enrichment techniques (HILIC and ZIC-HILIC) and 1D RPLC-MS/MS, which required only about one-third of the measurement time of the combination of all the methods in this study. This optimized workflow was able to cover 80% of the initially identified N-glycosylation sites, and facilitated the identification of over 6000 N-glycosylation sites on about 3000 N-glycoproteins from a single tissue. To the best of our knowledge, this is the largest N-glycoproteome dataset to date. Our dataset covered 49% of the mouse N-glycoproteins predicted in Swiss-Prot (released 2015_07), and contributed an additional 2208 experimentally identified N-glycosylation proteins. Notably, our dataset is only from one tissue, so many more N-glycoproteins remain to be explored in other tissues.

The in-depth mapping of the mouse brain N-glycoproteome revealed the widespread N-glycosylation of diverse brain proteins, including those involved in canonical pathways of brain physiology and neurological disease. Important biomarkers in various diseases were also found to be N-glycosylated. Investigating the N-glycosylation statuses of these important biomarkers under different disease conditions may improve their specificity and sensitivity as diagnostic markers. Little effort has been made to perform a large-scale characterization of N-glycoproteins in the nervous system. Our results have demonstrated the wide distribution of N-glycosylation in the brain proteome and may help to elucidate the biological functions of N-glycosylation in brain pathology and neurological disease.

## MATERIALS AND METHODS

### Protein extraction

Brain tissues from male C57BL/6 mouse, aged 3–12 months, were homogenized in lysis buffer (4% SDS, 0.1 M Tris/HCl, pH 7.6) by means of a high-throughput tissue grinding machine (ONEBIO, Shanghai, China) at 65 Hz for 60 s. The homogenates were sonicated and then clarified by centrifugation at 14000 rpm for 40 min. Protein concentrations were determined by the BCA method (Pierce, Rockford, IL).

### Protein digestion

Proteins were reduced in 10 mM DTT at 37°C for 60 min and then alkylated in the dark with 20 mM iodoacetamide at room temperature for 30 min. After the carbamidomethylation, six volumes of acetone were added to precipitate the proteins at − 20°C for at least 3 h. For the digestions with trypsin, Lys-C, chymotrypsin, trypsin coupled with Lys-C, and trypsin coupled with Glu-C, the precipitates were dissolved in a denaturing buffer (8 M urea in 50 mM NH_4_HCO_3_) and then diluted 10-fold with 50 mM NH_4_HCO_3_. For pepsin digestion, 1 M HCl was added to the above solution to a final concentration of 0.04 M. For Glu-C digestion, the precipitates were dissolved in another buffer (8 M urea in phosphate buffer) and then diluted 10-fold with phosphate buffer. The detailed digestion conditions of the seven sets of proteases are listed in Supplementary Table S1. The reactions were stopped by incubation at 95°C for 10 min. Finally, all digested samples were centrifuged at 14000 × g for 10 min, and the supernatants were desalted by means of Sep-Pak C 18 columns (Waters Corporation, Milford Massachusetts, USA) according to the manufacturer's instructions. The desalted samples were then dried by vacuum centrifugation and stored at − 20°C for further use.

### Glycopeptide enrichment

HILIC (Sepharose CL-4B for solid phase extraction of glycopeptides): Digested peptides (1 mg) were mixed with 100 μL Sepharose CL-4B in 500 μL of an organic solvent mixture containing butanol/ethanol/water (4:1:1 by volume). After being gently shaken for 60 min, the resins were washed thrice with the organic solvent. The resins were then incubated with an aqueous solvent of ethanol/H2O (1:1 by volume) for 30 min, and the solution phase was recovered.

ZIC-HILIC: Digested peptides (1 mg) dissolved in a loading buffer (80% CH_3_CN and 1% CF_3_COOH) were loaded onto a micro-column containing 30 mg ZIC-HILIC medium (5 μm, Merck Millipore, Darmstadt, Germany). After being washed with 0.8 mL loading buffer, the retained analysts were eluted with 1 mL 0.1% CF_3_COOH, followed by 20 μL 25 mM NH_4_HCO_3_ and 25 μL 50% CH_3_CN.

Hydrazide chemistry: Digested peptides (1 mg) were dissolved in 100 μL of coupling buffer (100 mM CH_3_COONa, 150 mM NaCl, pH 5.5). NaIO_4_ was introduced into the peptide solution at a final concentration of 10 mM, and the mixture was incubated in the dark at room temperature for 30 min with rotation. Then, Na_2_SO_3_ was added to a final concentration of 20 mM and incubated for 10 min. The coupling reaction was initiated by the introduction of 100 uL hydrazide resins (BioRad, USA) into the quenched peptide solution; then, extra coupling buffer was added to achieve a solid-to-liquid ratio of 1:5. The coupling reaction was performed at 37°C overnight with rotation. After the coupling reaction, the resins were thoroughly washed thrice with the following sequence of solutions: 1.5 M NaCl, 80% CH_3_CN, water, and 50 mM NH_4_HCO_3_. N-glycopeptides were released from the resins by the addition of PNGase F (500 units/μL, New England Biolabs, Ipswich, MA) at a concentration of 1 μL of PNGase F per mg of crude protein in 100 mM NH_4_HCO_3_ (pH 8.0) overnight at 37°C.

TiO_2_: Digested peptides were first treated with alkaline phosphatase (New England Biolabs, Ipswich, MA) at 30°C for 2 h. The peptide mixture was suspended in a loading buffer containing 1 M glycolic acid, 80% CH_3_CN, and 5% CF_3_COOH. The solution was then mixed with TiO_2_ beads (GL Sciences, USA) in batches (100 μg peptides: 6 mg TiO_2_ beads (5 μm)) and shaken gently at 25°C for 30 min. The beads were washed with loading buffer, followed by washing buffer 1 (80% CH_3_CN, 2% CF_3_COOH) and finally washing buffer 2 (20% CH_3_CN, 0.1% CF_3_COOH). Enriched peptides were eluted with a 0.5% (v/v) ammonia solution, pH 11.

### Deglycosylation of N-glycopeptides

The enriched glycopeptides were dissolved in 50 mM NH_4_HCO_3_ and deglycosylated with PNGase F at a concentration of 1 μL of PNGase F per mg of crude protein for 3 h at 37°C.

### Evaluation of false-positive rates

Enriched peptides obtained by HILIC, ZIC-HILIC, hydrazide chemistry or TiO_2_ chromatography were separated into equal aliquots. One aliquot was deglycosylated with PNGase F at 37°C for 3 h, while the other aliquot was incubated under the same conditions without PNGase F treatment. The two samples were then analyzed by LC-MS/MS in parallel.

### Fractionation with SCX chromatography before enrichment

Tryptic peptides were fractionated on a polySULFOETHYL A column (PolyLC 9.4 × 200 mm, 5 μm). The peptides were dissolved in SCX buffer A (7 mM KH_2_PO_4_, pH 2.65, 30% acetonitrile) before injection. Two minutes of isocratic buffer A were followed by a linear gradient from 0 to 25% of buffer B (7 mM KH_2_PO_4_, pH 2.65, 30% acetonitrile, 350 mM KCl) over 33 min and then several washing steps with 100% buffer B, 100% buffer C (H_2_O), and 100% buffer D (50 mM K_2_HPO_4_, 500 mM NaCl, pH 7.5). In total, 10 fractions (≈ 4-min intervals) were collected. The solvent was removed by vacuum concentration, and each fraction was desalted on a C18 Sep-Pak cartridge before enrichment.

### Fractionation with high-pH reversed-phase chromatography after enrichment

Enriched peptides were fractionated on a Waters UPLC with a C18 column (Waters BEH C18, 2.1 × 150 mm, 1.7 μm). The solvents consisted of 20 mM ammonium formate (pH 10) as mobile phase A and 100% CH_3_CN as mobile phase B. The flow rate was 600 μL/min with the following linear gradient: from 1 to 25% B in 16 min, from 25 to 45% B in 4 min, and from 45 to 90% B in 1 min. The collection and concatenation were accomplished in 1 min. Twenty fractions were collected during the LC separation, and pairs of fractions were combined as follows to generate 10 fractions: A (1, 11); B(2, 12); C(3, 13); … ; J(10, 20). Each fraction was then deglycosylated with PNGase F before MS analysis.

### Liquid chromatography-mass spectrometry (LC-MS)

N-glycopeptides were analyzed on a Triple-TOF^TM^ 4600 system (AB SCIEX, USA) or an Orbitrap Fusion Tribrid system (Thermo Fisher Scientific, USA). The Triple-TOF 4600 system was equipped with a nano-HPLC (Eksigent Technologies) with a reversed-phase analytical column (Eksigent, C18, 150 mm × 75 μm, 3 μm). The flow rate was 300 nL/min, with a gradient from 5 to 45% phase B (98% (v/v) acetonitrile with 0.1% (v/v) formic acid) in 95 min. An electrospray voltage of 2.5 kV versus the inlet of the mass spectrometer was used. The mass spectrometer was operated in information-dependent data acquisition mode to allow automatic switching between MS and MS/MS acquisition. MS spectra were acquired across the mass range of 350–1250 m/z with an accumulation time of 250 ms per spectrum. Tandem mass spectra were scanned from 100–1250 m/z in high-sensitivity mode with rolling collision energy. The 25 most-intense precursors in each cycle were selected for fragmentation, with a dynamic exclusion time of 25 s.

The Orbitrap Fusion was equipped with a Proxeon EASY-nLC II liquid chromatography pump (Thermo Fisher Scientific) involving a reversed-phase analytical column (Thermo Scientific, C18, 150 mm × 75 μm, 3 μm). Mobile phase buffer A was composed of water and 0.2% (v/v) formic acid, while mobile phase B was acetonitrile and 0.2% (v/v) formic acid. Samples were loaded onto the column for 3 min at 2 μL/min. The flow rate was 300 nL/min with the following linear gradient: from 2 to 20% B in 90 min, from 20 to 30% B in 15 min, from 30 to 45% B in 7 min, from 45 to 90% B in 2 min, followed by a 2-min wash at 90% B, then from 90 to 2% B in 1 min, and at last a 3-min re-equilibration at 2% B. Survey scans of peptide precursors from 350 to 1600 m/z were performed at 120-K resolution with a 3 × 10^5^ ion count target. Tandem MS was performed as follows: isolation at 1.6 Th with the quadrupole, HCD fragmentation with a normalized collision energy of 35%, and rapid-scan MS analysis in the ion trap. The MS^2^ ion count target was set to 10^4^ and the max injection time was 60 ms. Only those precursors with charge states of 2–5 were sampled for MS^2^. The dynamic exclusion duration was set to 30 s, with a 10-ppm tolerance around the selected precursor and its isotopes. Monoisotopic precursor selection was turned on. The instrument was run in top-speed mode with 3-s cycles, meaning that the instrument continuously performed MS^2^ events until either the list of non-excluded precursors was reduced to zero or 5 s had passed, whichever was shorter.

### Database searching

We searched for all tandem mass spectra in pFind studio 2.8 against the Swiss-Prot database (2015_03, mouse, 16711 entries), replacing the N in the sequon N-!P-[S|T|C] (where !P is ‘not proline’) with J. J was defined as having the same monoisotopic mass as asparagine, and variable modifications of 0.9840 were allowed only for J during database searching [41]. Static modification of carbamidomethyl (Cys) was set, along with dynamic modifications of deamidation (J), oxidation (Met), and acetylation (N-Terminal). For trypsin, Lys-C and Glu-C digestion, two missed cleavages were allowed. For chymotrypsin, pepsin, TryGlu and TryLys digestion, four missed cleavages were allowed. A precursor ion mass tolerance of 20 ppm and a fragment ion mass tolerance of 0.1 Da were set for the data from the Triple-TOF 4600, while the respective tolerances were set at 5 ppm and 0.25 Da for the data from the Orbitrap Fusion. A false discovery rate of 1% was estimated and applied to all data at the peptide-spectrum match level.

### Bioinformatics analyses

pLogo software was used to visualize the N-glycosylation sequence motifs surrounding the N-glycosites (seven amino acids on each side) [22]. Gene ontology enrichment analysis was performed with the DAVID Bioinformatics Resource (http://david.abcc.ncifcrf.gov/) [23]. In this manner, we determined which subcellular localizations, molecular functions and biological processes were enriched for the N-glycosylated proteins from our dataset in comparison to the entire mouse proteome. Ingenuity Systems Pathway Analysis software (Ingenuity Systems) was used to perform the biomarker filter analysis of the identified N-glycoproteins and the related pathway analyses.

## SUPPLEMENTARY MATERIALS FIGURES AND TABLES

























## References

[1] Zaia J (2008). Mass spectrometry and the emerging field of glycomics. Chem Biol.

[2] Ohtsubo K, Marth JD (2006). Glycosylation in cellular mechanisms of health and disease. Cell.

[3] Hwang H, Zhang J, Chung KA, Leverenz JB, Zabetian CP, Peskind ER, Jankovic J, Su Z, Hancock AM, Pan C, Montine TJ, Pan S, Nutt J (2010). Glycoproteomics in neurodegenerative diseases. Mass Spectrom Rev.

[4] Schedin-Weiss S, Winblad B, Tjernberg LO (2014). The role of protein glycosylation in Alzheimer disease. FEBS J.

[5] Liao J, Zhang R, Qian H, Cao L, Zhang Y, Xu W, Li J, Wu M, Yin Z (2012). Serum profiling based on fucosylated glycoproteins for differentiating between chronic hepatitis B and hepatocellular carcinoma. Biochem Biophys Res Commun.

[6] Hudak JE, Bertozzi CR (2014). Glycotherapy: new advances inspire a reemergence of glycans in medicine. Chem Biol.

[7] Zielinska DF, Gnad F, Wisniewski JR, Mann M (2010). Precision mapping of an *in vivo* N-glycoproteome reveals rigid topological and sequence constraints. Cell.

[8] Zielinska DF, Gnad F, Schropp K, Wisniewski JR, Mann M (2012). Mapping N-glycosylation sites across seven evolutionarily distant species reveals a divergent substrate proteome despite a common core machinery. Mol Cell.

[9] Parker BL, Palmisano G, Edwards AV, White MY, Engholm-Keller K, Lee A, Scott NE, Kolarich D, Hambly BD, Packer NH, Larsen MR, Cordwell SJ (2011). Quantitative N-linked glycoproteomics of myocardial ischemia and reperfusion injury reveals early remodeling in the extracellular environment. Mol Cell Proteomics.

[10] Kaji H, Shikanai T, Sasaki-Sawa A, Wen H, Fujita M, Suzuki Y, Sugahara D, Sawaki H, Yamauchi Y, Shinkawa T, Taoka M, Takahashi N, Isobe T (2012). Large-scale identification of N-glycosylated proteins of mouse tissues and construction of a glycoprotein database, GlycoProtDB. J Proteome Res.

[11] Chen W, Smeekens JM, Wu R (2014). A Universal Chemical Enrichment Method for Mapping the Yeast N-glycoproteome by MS. Mol Cell Proteomics.

[12] Zhu J, Sun Z, Cheng K, Chen R, Ye M, Xu B, Sun D, Wang L, Liu J, Wang F, Zou H (2014). Comprehensive mapping of protein N-glycosylation in human liver by combining hydrophilic interaction chromatography and hydrazide chemistry. J Proteome Res.

[13] Apweiler R, Hermjakob H, Sharon N (1999). On the frequency of protein glycosylation, as deduced from analysis of the SWISS-PROT database. Biochim Biophys Acta.

[14] Zhang Y, Zhang C, Jiang H, Yang P, Lu H (2015). Fishing the PTM proteome with chemical approaches using functional solid phases. Chem Soc Rev.

[15] Alley WR, Mann BF, Novotny MV (2013). High-sensitivity analytical approaches for the structural characterization of glycoproteins. Chem Rev.

[16] Chen R, Jiang X, Sun D, Han G, Wang F, Ye M, Wang L, Zou H (2009). Glycoproteomics analysis of human liver tissue by combination of multiple enzyme digestion and hydrazide chemistry. J Proteome Res.

[17] Zhao X, Ma C, Han H, Jiang J, Tian F, Wang J, Ying W, Qian X (2013). Comparison and optimization of strategies for a more profound profiling of the sialylated N-glycoproteomics in human plasma using metal oxide enrichment. Anal Bioanal Chem.

[18] Ma C, Zhao X, Han H, Tong W, Zhang Q, Qin P, Chang C, Peng B, Ying W, Qian X (2013). N-linked glycoproteome profiling of human serum using tandem enrichment and multiple fraction concatenation. Electrophoresis.

[19] Lewandrowski U, Zahedi RP, Moebius J, Walter U, Sickmann A (2007). Enhanced N-glycosylation site analysis of sialoglycopeptides by strong cation exchange prefractionation applied to platelet plasma membranes. Mol Cell Proteomics.

[20] Cao W, Cao J, Huang J, Zhang L, Yao J, Xu H, Yang P (2012). Enhanced N-glycosylation site exploitation of sialoglycopeptides by peptide IPG-IEF assisted TiO_2_ chromatography. Glycoconj J.

[21] Palmisano G, Melo-Braga MN, Engholm-Keller K, Parker BL, Larsen MR (2012). Chemical deamidation: a common pitfall in large-scale N-linked glycoproteomic mass spectrometry-based analyses. J Proteome Res.

[22] O'shea JP, Chou MF, Quader SA, Ryan JK, Church GM, Schwartz D (2013). pLogo: a probabilistic approach to visualizing sequence motifs. Nat Methods.

[23] Huang da W, Sherman BT, Lempicki RA (2009). Systematic and integrative analysis of large gene lists using DAVID bioinformatics resources. Nat Protoc.

[24] Malenka RC, Bear MF (2004). LTP and LTD: an embarrassment of riches. Neuron.

[25] Sweatt JD (1999). Toward a molecular explanation for long-term potentiation. Learn Mem.

[26] Jiang J, Suppiramaniam V, Wooten MW (2006). Posttranslational modifications and receptor-associated proteins in AMPA receptor trafficking and synaptic plasticity. Neurosignals.

[27] Gu W, Fukuda T, Isaji T, Hang Q, Lee HH, Sakai S, Morise J, Mitoma J, Higashi H, Taniguchi N, Yawo H, Oka S, Gu J (2015). Loss of alpha1,6-fucosyltransferase decreases hippocampal long term potentiation: implictions for core fucosylation in the regulation of AMPA receptor heteromerization and cellular signaling. J Biol Chem.

[28] Matthies HJ, Kretlow J, Matthies H, Smalla KH, Staak S, Krug M (1999). Glycosylation of proteins during a critical time window is necessary for the maintenance of long-term potentiation in the hippocampal CA1 region. Neuroscience.

[29] Nabavi S, Fox R, Proulx CD, Lin JY, Tsien RY, Malinow R (2014). Engineering a memory with LTD and LTP. Nature.

[30] Goedert M, Spillantini MG (2006). A century of Alzheimer's Disease. Science.

[31] Querfurth HW, LaFerla FM (2010). Mechanisms Of Disease Alzheimer's Disease. New Engl J Med.

[32] Butterfield DA, Owen JB (2011). Lectin-affinity chromatography brain glycoproteomics and Alzheimer disease: insights into protein alterations consistent with the pathology and progression of this dementing disorder. Proteomics Clin Appl.

[33] Botella-Lopez A, Burgaya F, Gavin R, Garcia-Ayllon MS, Gomez-Tortosa E, Pena-Casanova J, Urena JM, Del Rio JA, Blesa R, Soriano E, Saez-Valero J (2006). Reelin expression and glycosylation patterns are altered in Alzheimer's disease. Proc Natl Acad Sci.

[34] Sáez-Valero J, Sberna G, McLean CA, Small DH (1999). Molecular Isoform Distribution and Glycosylation of Acetylcholinesterase Are Altered in Brain and Cerebrospinal Fluid of Patients with Alzheimer's Disease. J Neurochem.

[35] Palmigiano A, Barone R, Sturiale L, Sanfilippo C, Bua RO, Romeo DA, Messina A, Capuana ML, Maci T, Le Pira F, Zappia M, Garozzo D (2016). CSF N-glycoproteomics for early diagnosis in Alzheimer's disease. J Proteomics.

[36] Kizuka Y, Kitazume S, Fujinawa R, Saito T, Iwata N, Saido TC, Nakano M, Yamaguchi Y, Hashimoto Y, Staufenbiel M, Hatsuta H, Murayama S, Manya H (2015). An aberrant sugar modification of BACE1 blocks its lysosomal targeting in Alzheimer's disease. EMBO Mol Med.

[37] Liu Y, Qing H, Deng Y (2014). Biomarkers in Alzheimer's disease analysis by mass spectrometry-based proteomics. Int J Mol Sci.

[38] Mueller TM, Haroutunian V, Meador-Woodruff JH (2014). N-Glycosylation of GABAA receptor subunits is altered in Schizophrenia. Neuropsychopharmacol.

[39] Freeze HH, Eklund EA, Ng BG, Patterson MC (2015). Neurological aspects of human glycosylation disorders. Annu Rev Neurosci.

[40] Weng Y, Sui Z, Jiang H, Shan Y, Chen L, Zhang S, Zhang L, Zhang Y (2015). Releasing N-glycan from peptide N-terminus by N-terminal succinylation assisted enzymatic deglycosylation. Sci Rep.

[41] Atwood JA, Sahoo SS, Alvarez-Manilla G, Weatherly DB, Kolli K, Orlando R, York WS (2005). Simple modification of a protein database for mass spectral identification of N-linked glycopeptides. Rapid Commun Mass Spectrom.

