# Supplementary material for: In-depth mapping of the mouse brain N-glycoproteome reveals widespread N-glycosylation of diverse brain proteins
Source: Oncotarget. 2016 May 31;7(25):38796–809. doi: 10.18632/oncotarget.9737 (PMC5122430; doi:10.18632/oncotarget.9737)
Supplement: Supplementary file 1 [file oncotarget-07-38796-s001.pdf]

In-depth mapping of the mouse brain N-glycoproteome reveals widespread N-glycosylation of diverse brain proteins

Supplementary Materials

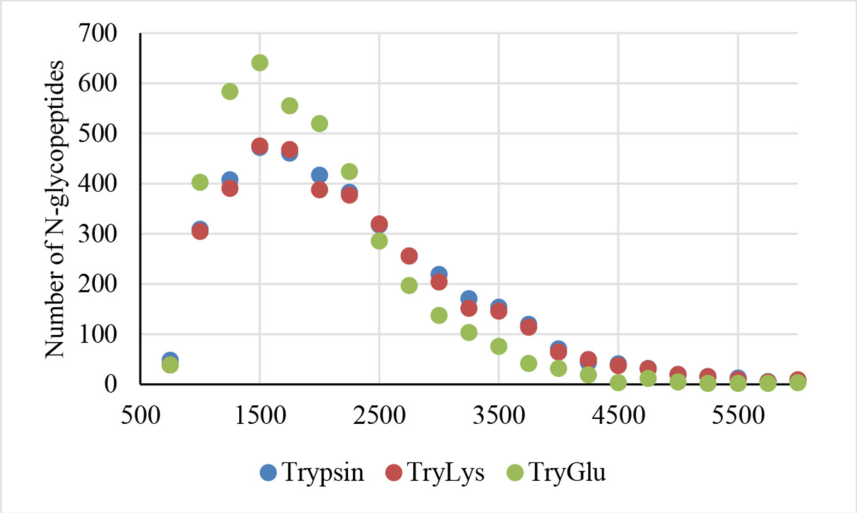

Supplementary Figure S1: Molecular weight distribution of identified N-glycopeptides from trypsin, TryLys and TryGlu (Y-axis shows the number of N-glycopeptides in 250 unit of mass).

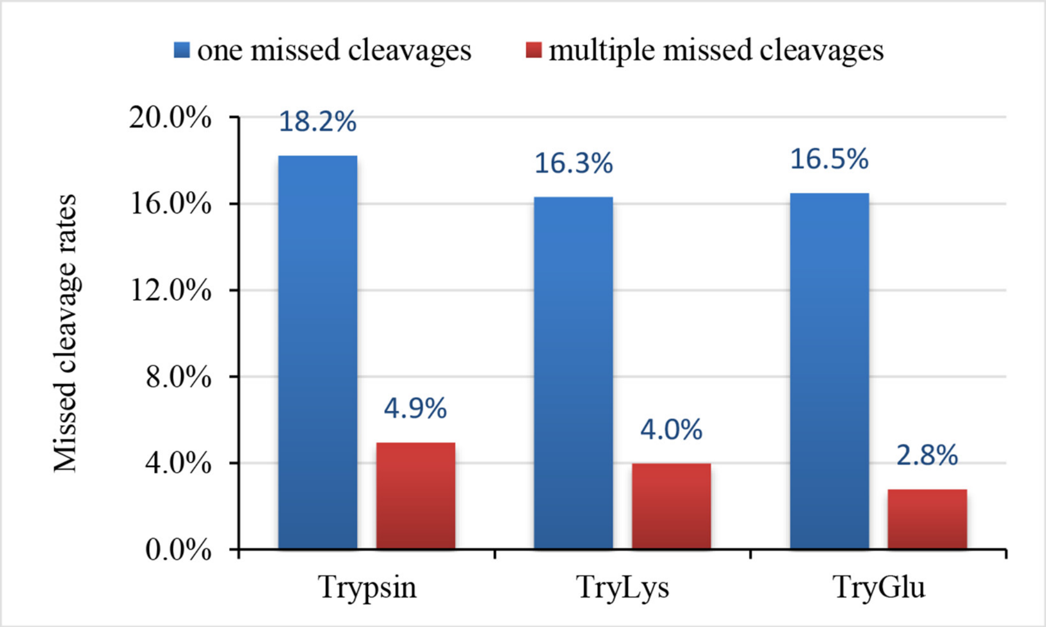

Supplementary Figure S2: The missed cleavage rates from trypsin, TryLys and TryGlu.

Supplementary Table S1: Digestion conditions used

Proteases	Supplier	Protein to enzyme ratio	Buffer	Digestion time, h	Digestion temp, °C	Cleavage sites
Trypsin	Promega	1:50	50 mM $(NH)_4HCO_3$	12	37	K, R
Trypsin coupled with Lys-C	Promega/ Wako	1:50/1:100	50 mM $(NH)_4HCO_3$	12	37	K, R
Trypin coupled with Glu-C	Promega/ Promega	1:50/1:100	50 mM $(NH)_4HCO_3$	12	37	K, R, D, E
Lys-C	Wako	1:50	50 mM $(NH)_4HCO_3$	12	37	K
Glu-C	Promega	1:50	phosphate buffer	12	37	D, E
Chymotrypsin	Promega	1:50	50 mM $(NH)_4HCO_3$ 10 mM $CaCl_2$	12	25	F, L, W, Y
Pepsin	Promega	1:50	0.04 M HCl	12	37	F, L, Y, W

Supplementary Table S2: The false positive rates detected for ZIC-HILIC, HILIC, hydrazide chemistry and TiO2

Enrichment techniques	Number of N-glycopeptides with PNGase F treatment	Number of N-glycopeptides without PNGase F treatment	Rates
ZIC-HILIC	57	3353	1.7%
HILIC	107	2697	4%
Hydrazide chemistry	6	774	0.7%
TiO_2	31	433	7.1%

Supplementary Table S3: Potential false postive N-linked glycosylation sites identified by direct analysis of enriched glycopeptides (ZIC-HILIC, Sepharose CL-4B, hydrazide chemistry and TiO2 preparations) by LC-ESI-MS/MS without PNGase F treatment. See Supplementary_Table_S3

Supplementary Table S4: N-glycopeptides identified from all methods in this study. The yellow marker represents the optimized workflow. See Supplementary_Table_S4

Supplementary Table S5: Gene Ontology enrichment analysis of identified N-glycoproteins in mouse brain. See Supplementary_Table_S5

Supplementary Table S6: All canonical pathways that the identified N-glycoproteins were significantly associated with. See Supplementary_Table_S6

Supplementary Table S7: Canonical pathways that the identified N-glycoproteins were associated with in nervous system signaling. See Supplementary_Table_S7

Supplementary Table S8: N-glycosylated proteins in synaptic long-term potentiation. See Supplementary_Table_S8

Supplementary Table S9: N-glycosylated proteins in synaptic long-term depression. See Supplementary_Table_S9

Supplementary Table S10: Glutamate receptors (GluRs) identified in this study. See Supplementary_ Table_S10

Supplementary Table S11: N-glycosylated proteins in amyloid processing. See Supplementary_ Table_S11

Supplementary Table S12: Diseased-related biomarkers by IPA filter. See Supplementary_Table_ S12

Supplementary Table S13: N-glycoproteins related to Alzheimer's disease. See Supplementary_ Table_S13
