# Supplementary material for: In-depth mapping of the mouse brain N-glycoproteome reveals widespread N-glycosylation of diverse brain proteins
Source: Oncotarget. 2016 May 31;7(25):38796–809. doi: 10.18632/oncotarget.9737 (PMC5122430; doi:10.18632/oncotarget.9737)
Supplement: Supplementary file 2 [file oncotarget-07-38796-s002.docx]

| Supplementary Table S3: Potential false postive N-linked glycosylation sites identified by direct analysis of enriched glycopeptides (ZIC-HILIC, Sepharose CL-4B, hydrazide chemistry and TiO2 preparations) by LC-ESI-MS/MS without PNGase F treatment | | | | | | | |
| --- | --- | --- | --- | --- | --- | --- | --- |
| **Sequence** | **Spectrum counts** | **Score** | **Protein** | **Unique** | **Pre:Next** | **Start position** | **Glycosites** |
| ACVIHGTDLKDFTSEQID EILQJHTEIVFAR | 2 | 4.44E-10 | sp\|Q6PIC6\|AT1A3_MOUSE | Yes | K:T | 651 | 674 |
| AEDJSCHLAPVSIPEPHTATV SPPTDEAAGEAGLTDESPAG NLPGSSFSHSALSGDR | 1 | 2.78E-14 | sp\|Q9QYR6\|MAP1A_MOUSE | Yes | K:K | 1229 | 1233 |
| AEDSSDRPLEGHSYQJSSGD NSQNSNIDQSFWETFGSAEPPK | 3 | 1.96E-16 | sp\|Q9EPJ9\|ARFG1_MOUSE | Yes | K:A | 297 | 313 |
| AFAMIIDKLEEDISSSMTJST AASRPPVTLR | 2 | 7.55E-10 | sp\|Q61990\|PCBP2_MOUSE | Yes | K:L | 70 | 89 |
| AVCMLSJTTAIAEAWAR | 2 | 8.90E-08 | sp\|P05213\|TBA1B_MOUSE | No | R:L | 373 | 380 |
| AYHEQLSVAEITJACFEPANQ MVK | 13 | 5.78E-13 | sp\|P05213\|TBA1B_MOUSE | No | K:C | 280 | 293 |
| CFHJMSDVTWSASVFFPLEIF GFLLPMGIMGFCSYR | 1 | 2.39E-07 | sp\|Q3UJF0\|GPR55_MOUSE | Yes | K:S | 166 | 170 |
| DDDIAALVVDJGSGMCK | 1 | 6.37E-11 | sp\|P60710\|ACTB_MOUSE | Yes | M:A | 1 | 12 |
| DKEJGSVSTSETPPPPPPNEIS PPHAVEEEEDDDWGEDTTEE AQR | 4 | 4.10E-10 | sp\|P59325\|IF5_MOUSE | Yes | K:R | 166 | 170 |
| EASDIILTDDJFSSIVK | 1 | 3.91E-08 | sp\|Q9R0K7\|AT2B2_MOUSE | Yes | K:A | 801 | 812 |
| ECISIHVGQAGVQIGJACWEL YCLEHGIQPDGQMPSDK | 1 | 3.24E-08 | sp\|P05213\|TBA1B_MOUSE | No | R:T | 2 | 18 |
| ECISVHVGQAGVQMGJACW ELYCLEHGIQPDGQMPSDK | 1 | 1.92E-07 | sp\|P68368\|TBA4A_MOUSE | Yes | R:T | 2 | 18 |
| EELQAJGSAPAADKEEPASGS AATPAAAEKDEAAAATEPGA GAADK | 5 | 1.80E-12 | sp\|P26645\|MARCS_MOUSE | Yes | K:E | 55 | 61 |
| EQHQHSDLDSJQTHSAGTM TSSSSTTNIDDLK | 1 | 1.46E-08 | sp\|A2AGT5\|CKAP5_MOUSE | Yes | R:K | 1989 | 2000 |
| ESNSJQCVYAAHNSVVIDECK | 3 | 4.07E-08 | sp\|P43006\|EAA2_MOUSE | Yes | R:V | 528 | 533 |
| HPJGTQETILLJHTFJETQIEWFR | 7 | 8.23E-11 | sp\|Q99KI0\|ACON_MOUSE | Yes | K:A | 743 | 746 |
| HTGPGILSMANAGPNTJGSQF FICTAK | 4 | 4.90E-12 | sp\|P17742\|PPIA_MOUSE | Yes | K:T | 91 | 108 |
| HTSSSGAAEGASCSERPAGSL ACPSPJCSPLPETPR | 2 | 2.61E-12 | sp\|Q3UNH4\|GRIN1_MOUSE | Yes | R:A | 28 | 55 |
| ILDLGITGPEGHVLSRPEEVE AEAVJR | 1 | 1.02E-07 | sp\|O08553\|DPYL2_MOUSE | Yes | R:S | 211 | 237 |
| IVSJASCTTNCLAPLAK | 1 | 1.99E-06 | sp\|P16858\|G3P_MOUSE | Yes | K:V | 143 | 147 |
| JESCSVJYTTDFIYQLYSEEGK | 1 | 4.65E-07 | sp\|Q9WUA3\|PFKAP_MOUSE | Yes | R:G | 636 | 637 |
| JHSGGGGSGSGGSSGGGGGS GGSGASSGGSSSHGSGPSSCS SGPSSSR | 1 | 4.25E-18 | sp\|Q0KL02\|TRIO_MOUSE | Yes | R:S | 2288 | 2289 |
| JITFFSTNCVEGTAR | 1 | 2.41E-08 | sp\|Q6PIC6\|AT1A3_MOUSE | Yes | R:G | 230 | 231 |
| KAADAHVDAHYYEQNEQPTGTCA ACITGGJR | 1 | 5.33E-11 | sp\|P55264\|ADK_MOUSE | Yes | R:S | 117 | 147 |
| KAJSTQEPSQQPASSGASPLSASEGP ESPGSSRPSVAGLR | 1 | 1.86E-09 | sp\|Q8CI51\|PDLI5_MOUSE | Yes | K:S | 304 | 307 |
| KFVEVMSEYJATQSDYR | 1 | 4.90E-08 | sp\|O35526\|STX1A_MOUSE | Yes | R:E | 125 | 135 |
| KSPLHPIPASPTSPQSGLDGSJSTLSG SASSGVSSLSESNFGHSSEAPPR | 1 | 2.58E-09 | sp\|Q8CIQ7\|DOCK3_MOUSE | Yes | R:T | 1857 | 1879 |
| LAAAYNLLQHGITJLCVIGGDGSLT GANIFR | 1 | 5.89E-09 | sp\|P12382\|PFKAL_MOUSE | Yes | R:N | 98 | 112 |
| LJSSSSSSJSSNEREDFDSTSSS STPPQPR | 1 | 2.34E-13 | sp\|A2A432\|CUL4B_MOUSE | Yes | K:D | 115 | 117 |
| LVJGCALNFFR | 1 | 1.20E-09 | sp\|Q9DCJ5\|NDUA8_MOUSE | Yes | K:Q | 61 | 64 |
| LWGDHGQEALESAHVCLIJAT ATGTEILK | 1 | 7.53E-08 | sp\|Q8VBW6\|ULA1_MOUSE | Yes | R:N | 18 | 37 |
| MAATFIGJSTAIQELFK | 1 | 1.06E-06 | sp\|Q9D6F9\|TBB4A_MOUSE | Yes | K:R | 362 | 370 |
| MAGNEYVGFSJATFQSER | 1 | 9.45E-09 | sp\|D3Z7P3\|GLSK_MOUSE | Yes | K:E | 369 | 380 |
| MTVAHMWFDNQIHEADTTEJ QSGVSFDK | 1 | 1.44E-09 | sp\|Q8VDN2\|AT1A1_MOUSE | Yes | R:T | 385 | 405 |
| NDLJHSTQFVQGLALCTLGC MGSSEMCR | 1 | 1.82E-13 | sp\|P22892\|AP1G1_MOUSE | Yes | K:D | 108 | 112 |
| NPGSTNHIPFHEDSPCGSQJSSEHS VIKPSPGDSSGJLSR | 3 | 2.48E-10 | sp\|Q8CGF6\|WDR47_MOUSE | Yes | R:S | 545 | 565 |
| NPHVQQAAQNAAMGAAQGAMNQP QTQYSATPJYTYSNEM | 2 | 7.24E-12 | sp\|Q9JKD3\|SCAM5_MOUSE | Yes | K:- | 196 | 228 |
| PAKPQLAQKPSQDVPPPITAAAGG PPHPQLJK | 1 | 7.38E-09 | sp\|O88935\|SYN1_MOUSE | Yes | R:S | 631 | 662 |
| PRYQMTVLHIHEDPEALSSPVGGTGSN JESSPSNIDWEIETDNSDSPAGGDMK | 1 | 3.55E-07 | sp\|Q52KR3\|PRUN2_MOUSE | Yes | K:P | 2430 | 2458 |
| QISYJCSDLDQSJVTEETPEGEEHPVADT ENKENEVEEVKEEGPK | 1 | 1.54E-10 | sp\|Q9CY58\|PAIRB_MOUSE | Yes | K:E | 240 | 245 |
| RPEJETVLHQFCCPAADTEQKPACSDLA SQSDGSCAQAGGGMEDSVVAAVAAGR | 1 | 1.19E-12 | sp\|Q3TES0\|IQEC3_MOUSE | Yes | R:P | 177 | 181 |
| RPLVLQLVJSTTEYAEFLHCK | 1 | 4.97E-11 | sp\|P39053\|DYN1_MOUSE | Yes | R:G | 66 | 75 |
| SAPAQPPAEGTEGAAPGGGPPGP PPJMTSNRR | 1 | 4.05E-07 | sp\|Q62442\|VAMP1_MOUSE | Yes | M:L | 1 | 27 |
| SPAPDAPTDTASPPJVSPSSSSPASPATGH TRPSSLHGLAAK | 1 | 1.11E-12 | sp\|Q3U214\|MAST3_MOUSE | Yes | R:L | 1139 | 1154 |
| SSCSSCSQSGSADGGSTJGCNHER | 1 | 6.45E-12 | sp\|Q8BPQ7\|SGSM1_MOUSE | Yes | K:A | 453 | 471 |
| SSSPSEHLSPQQDGVDTSCSTGSP GVATAJR | 1 | 4.19E-08 | sp\|Q91X56\|S1PR5_MOUSE | Yes | R:S | 361 | 391 |
| STLMNTLFJTTFETEEASHHEECVR | 1 | 1.35E-11 | sp\|Q8CHH9\|SEPT8_MOUSE | Yes | K:L | 57 | 66 |
| TASEMVLADDJFSTIVAAVEEGR | 1 | 2.59E-11 | sp\|O55143\|AT2A2_MOUSE | No | K:A | 727 | 738 |
| TFHETLJCCGSNALTTLTTTILR | 3 | 3.28E-08 | sp\|P35762\|CD81_MOUSE | Yes | K:N | 148 | 155 |
| TJDSTEQJVSDGTPMPDSYPTTPSSTDA PTSESK | 1 | 7.37E-10 | sp\|Q7TMY8\|HUWE1_MOUSE | Yes | K:E | 2725 | 2727 |
| TNRPLPPPPSQRPAEQPPVVGQVQEAPS IGLJNSHK | 1 | 1.70E-09 | sp\|Q811P8\|RHG32_MOUSE | Yes | R:V | 1305 | 1337 |
| VAKPTSMYINDGYAPPDITJSSSQSVDN HVSPSSCLGQTPTSPAR | 1 | 4.75E-08 | sp\|Q811D0\|DLG1_MOUSE | Yes | K:Y | 401 | 421 |
| VFGSQJLTTVK | 1 | 9.59E-08 | sp\|Q9JKR6\|HYOU1_MOUSE | Yes | R:L | 509 | 515 |
| VFVGYJSTGAELR | 1 | 1.77E-07 | sp\|P46096\|SYT1_MOUSE | Yes | K:H | 375 | 381 |
| VLVAPPSEEAJTTK | 1 | 2.10E-10 | sp\|P43006\|EAA2_MOUSE | Yes | K:A | 194 | 205 |
| ADPVLLNJHSNLKPAPTVPAA PSSPDATSEPK | 1 | 9.19E-08 | sp\|Q9JKC6\|CEND_MOUSE | Yes | K:G | 66 | 74 |
| AFQFVETHGEVCPAJWTPESP TIKPSPTASK | 1 | 7.14E-10 | sp\|P20108\|PRDX3_MOUSE | Yes | K:E | 218 | 233 |
| AHQLVMEGYJWCHDR | 1 | 2.43E-10 | sp\|P62715\|PP2AB_MOUSE | No | R:N | 239 | 249 |
| AIAHYEQSADYYKGEESJSSAJK | 3 | 1.19E-11 | sp\|P28663\|SNAB_MOUSE | No | K:C | 140 | 158 |
| AKDKESLDPSNLMPPPJQTPS PDQPFTLSTSR | 1 | 2.15E-08 | sp\|P53702\|CCHL_MOUSE | Yes | R:E | 76 | 93 |
| APEPNJHTEYASIETGK | 2 | 5.15E-13 | sp\|P97797\|SHPS1_MOUSE | Yes | R:V | 454 | 460 |
| APISLSQDPSPPLJGSTTSCGPDR | 1 | 4.84E-09 | sp\|Q9QYR6\|MAP1A_MOUSE | Yes | R:R | 2038 | 2052 |
| AQEHQEPAESALKGETGA LHAJTSGSPSVR | 1 | 2.32E-08 | sp\|Q8BMS4\|COQ3_MOUSE | Yes | R:E | 335 | 357 |
| AQTTNSJSSSSSDVSTHS | 1 | 4.81E-11 | sp\|Q80TV8\|CLAP1_MOUSE | Yes | R:- | 1517 | 1524 |
| AREEJGSQLYTTJMCAGQYEELPHK | 2 | 1.12E-08 | sp\|Q8C419\|GP158_MOUSE | Yes | R:V | 1092 | 1097 |
| ARPSQLPEQSSSAQQJGSVSD ISPVQAAK | 2 | 1.94E-11 | sp\|P28740\|KIF2A_MOUSE | Yes | R:K | 116 | 132 |
| ASJNTASIAQAR | 2 | 1.57E-08 | sp\|P63213\|GBG2_MOUSE | Yes | M:K | 1 | 4 |
| AVISMLJETMNEAPEETK | 1 | 4.80E-08 | sp\|P43006\|EAA2_MOUSE | Yes | K:I | 208 | 215 |
| CJVSSEGSEHSLEGQK | 1 | 7.19E-10 | sp\|B9EKR1\|PRPTZ_MOUSE | Yes | K:F | 132 | 134 |
| CSAJCCEDTQASMQQVHQCIER | 1 | 7.73E-10 | sp\|Q9CR98\|F136A_MOUSE | Yes | R:C | 34 | 38 |
| DAMVGJYTCEVTELSR | 1 | 1.30E-08 | sp\|Q61735\|CD47_MOUSE | Yes | R:E | 103 | 109 |
| DJFTLIPEGTJGTEER | 2 | 3.95E-15 | sp\|O08553\|DPYL2_MOUSE | Yes | K:M | 345 | 347 |
| DQHJGSLTDPSSVHEK | 1 | 2.03E-07 | sp\|Q99KU0\|VMP1_MOUSE | Yes | K:K | 17 | 21 |
| DSPACCSTNLHSEJHSDSSDSGNYDAPV GGDSLLGDCELSR | 1 | 4.38E-13 | sp\|B1AVZ0\|UPP_MOUSE | Yes | R:Q | 64 | 78 |
| EEEIAALVIDJGSGMCK | 1 | 2.57E-15 | sp\|P63260\|ACTG_MOUSE | Yes | M:A | 1 | 12 |
| ESSESTJTTIEDEDTK | 2 | 3.75E-09 | sp\|P11798\|KCC2A_MOUSE | Yes | K:V | 328 | 335 |
| ESTDESEVDKTHCLJNSVSSGTYSDYSP SQASSASSNTR | 1 | 1.82E-07 | sp\|Q80TE7\|LRRC7_MOUSE | Yes | K:M | 664 | 679 |
| EYVJSTSEESHDEDEIRPVQQQDLHR | 2 | 1.77E-08 | sp\|Q9D5T0\|ATAD1_MOUSE | Yes | R:A | 312 | 316 |
| FQGNANDPDQNGJNTQSPPSR | 2 | 5.57E-11 | sp\|B9EJA2\|CTTB2_MOUSE | Yes | R:D | 448 | 461 |
| GAQEJGTGQATSR | 1 | 1.41E-07 | sp\|Q64314\|CD34_MOUSE | Yes | R:N | 354 | 359 |
| GDDASEEGQJGSSPK | 1 | 1.42E-07 | sp\|Q9QYC0\|ADDA_MOUSE | Yes | R:S | 452 | 462 |
| GGGGJSSSSGSGSGSGSGSPSTGSS GSSSSPGAR | 2 | 3.90E-09 | sp\|Q8BSY0\|ASPH_MOUSE | Yes | K:R | 8 | 13 |
| GSSJGCTAAQPR | 1 | 1.24E-10 | sp\|Q80TK0\|K1107_MOUSE | Yes | R:T | 440 | 444 |
| GVGIISEGJETVEDIAAR | 2 | 6.07E-14 | sp\|Q6PIC6\|AT1A3_MOUSE | No | K:L | 619 | 628 |
| HEYQANGPEDLJR | 1 | 1.59E-10 | sp\|Q9R0P5\|DEST_MOUSE | Yes | K:T | 132 | 144 |
| HHAAYVNNLJATEEK | 1 | 2.87E-08 | sp\|P09671\|SODM_MOUSE | Yes | K:Y | 53 | 63 |
| HPEKEHQEGVPNSDEGYWDSTTPGP EEESISJSSSSK | 1 | 2.50E-08 | sp\|Q8CCJ4\|AMER2_MOUSE | Yes | K:K | 508 | 540 |
| HSJHSTENSPIER | 1 | 9.08E-12 | sp\|Q8R0S4\|CACB4_MOUSE | Yes | R:R | 438 | 441 |
| IDASKNEEDEGHSJSSPR | 2 | 3.71E-19 | sp\|Q60668\|HNRPD_MOUSE | Yes | K:H | 67 | 81 |
| ILQEYQVQYTPQGDSDDGKGJQTVHK | 2 | 1.94E-09 | sp\|D3Z7P3\|GLSK_MOUSE | Yes | K:N | 642 | 663 |
| IPAVPJGTAAHGEAEPHGGHSGPELQR | 1 | 4.20E-07 | sp\|Q5DTL9\|S4A10_MOUSE | Yes | K:T | 451 | 457 |
| JQTAEKEEFEHQQK | 1 | 9.11E-10 | sp\|P63017\|HSP7C_MOUSE | Yes | K:E | 583 | 584 |
| KADGVKPQTJSTK | 2 | 2.33E-12 | sp\|P28652\|KCC2B_MOUSE | Yes | K:J | 347 | 357 |
| KAEGATLSJATGAVESTSQAGDR | 4 | 5.48E-25 | sp\|Q61644\|PACN1_MOUSE | Yes | K:G | 318 | 327 |
| KDCSJGCSAPFAGER | 1 | 8.48E-14 | sp\|P24288\|BCAT1_MOUSE | Yes | M:G | 1 | 6 |
| KFEHQLLYJACQLDNPDEQAAQIR | 1 | 7.81E-12 | sp\|Q80TJ1\|CAPS1_MOUSE | Yes | K:R | 282 | 291 |
| KHJMCGDTEEEK | 1 | 1.20E-07 | sp\|P48774\|GSTM5_MOUSE | Yes | R:I | 85 | 88 |
| KKPTPIQLNPAPDGSAVJGTSSAE TNLEALQK | 4 | 4.26E-12 | sp\|P31938\|MP2K1_MOUSE | Yes | K:K | 3 | 21 |
| KLEHQLLYJACQLDNADEQAAQIR | 1 | 7.14E-11 | sp\|Q8BYR5\|CAPS2_MOUSE | Yes | K:R | 253 | 262 |
| KMJDSJSSGAGGPVK | 2 | 1.47E-12 | sp\|P61028\|RAB8B_MOUSE | Yes | R:I | 175 | 178 |
| KQEEFDIANJGSSQANK | 1 | 8.10E-12 | sp\|Q64514\|TPP2_MOUSE | Yes | R:L | 151 | 161 |
| KSPSEGTDEKAJGTHPK | 1 | 5.63E-09 | sp\|Q2PFD7\|PSD3_MOUSE | Yes | K:T | 735 | 747 |
| KTEVKPSSJGSASSASK | 1 | 5.19E-08 | sp\|Q9DAV9\|TM38B_MOUSE | Yes | K:R | 258 | 267 |
| KVVVCDJGTGFVK | 1 | 1.43E-11 | sp\|P61161\|ARP2_MOUSE | Yes | R:C | 6 | 13 |
| LLLIGJSSVGK | 1 | 2.83E-07 | sp\|P35276\|RAB3D_MOUSE | Yes | K:T | 24 | 30 |
| LVTMLJASGSTHFTRMRR | 1 | 9.73E-07 | sp\|P53349\|M3K1_MOUSE | Yes | K:R | 832 | 838 |
| MASILPASJRSMRPDK | 1 | 1.70E-08 | sp\|Q8CGR7\|UPP2_MOUSE | Yes | -:N | 0 | 9 |
| MDPJCSCSTGGSCTCTSSCACK | 1 | 8.88E-10 | sp\|P02802\|MT1_MOUSE | Yes | -:N | 0 | 4 |
| MEMVFGLDVKEVDPIJHCYALFIK LGLTYDGMR | 1 | 9.14E-07 | sp\|Q9CWV4\|MAGBG_MOUSE | Yes | R:N | 176 | 192 |
| MEQMAJMTLMKETITTVEKEMK | 1 | 1.14E-07 | sp\|Q6NY15\|TSG10_MOUSE | Yes | R:S | 158 | 164 |
| MGSSHAINQNVJQSLCQEDDYED DKPTJYSER | 1 | 4.19E-07 | sp\|Q61315\|APC_MOUSE | Yes | R:Y | 1112 | 1124 |
| MITVVYGPDLVJISHLNLVAFQEEVAKE WTNEVFSLATNLLAQJMSR | 1 | 1.70E-07 | sp\|Q9Z1B3\|PLCB1_MOUSE | Yes | R:D | 101 | 113 |
| MMJGSHYSYSESR | 2 | 5.32E-11 | sp\|Q99LX0\|PARK7_MOUSE | Yes | K:V | 132 | 135 |
| NJGSJJSSVTSFSTPPQDSSQR | 3 | 2.27E-11 | sp\|Q8CGF6\|WDR47_MOUSE | Yes | K:L | 509 | 511 |
| NKQELDINJITTYKK | 1 | 2.45E-08 | sp\|P12658\|CALB1_MOUSE | Yes | K:N | 221 | 230 |
| NNMQSGVJNTKK | 1 | 7.84E-08 | sp\|Q9CXZ1\|NDUS4_MOUSE | Yes | R:W | 84 | 92 |
| NQQITHAJNTVSSFK | 1 | 2.54E-07 | sp\|Q61699\|HS105_MOUSE | Yes | K:R | 53 | 61 |
| NQRJESSHGGEFGCTMEELR | 1 | 6.21E-10 | sp\|Q9R0K7\|AT2B2_MOUSE | Yes | K:S | 12 | 16 |
| NRPETSLJQSJVTTEDMYTJGSPA PGSPAHAK | 6 | 1.15E-13 | sp\|P70290\|EM55_MOUSE | Yes | R:G | 30 | 38 |
| NTEJGSAPSTAPTDQNR | 2 | 1.28E-13 | sp\|Q91YL2\|RN126_MOUSE | Yes | R:Q | 45 | 49 |
| NTNDAJSCQIIIPQNQVNR | 4 | 4.17E-14 | sp\|P50396\|GDIA_MOUSE | No | K:K | 309 | 315 |
| NVIIWGJHSSTQYPDVNHAK | 1 | 5.22E-11 | sp\|P14152\|MDHC_MOUSE | Yes | K:V | 179 | 186 |
| RGJJTTSLLSQSVAK | 1 | 2.38E-07 | sp\|Q9DB77\|QCR2_MOUSE | Yes | K:G | 300 | 303 |
| RJLSADQWR | 1 | 1.45E-09 | sp\|P28660\|NCKP1_MOUSE | Yes | R:N | 216 | 218 |
| RPEAVQJTSVDASEDQKDEK | 2 | 7.08E-11 | sp\|Q922S4\|PDE2A_MOUSE | Yes | R:G | 176 | 183 |
| RTJSTGGSSGNSVGGGSGK | 1 | 1.36E-14 | sp\|Q99MI1\|RB6I2_MOUSE | Yes | R:T | 33 | 36 |
| SATAATVPPAAPAGEGGPPAP PPJLTSNRR | 1 | 4.29E-08 | sp\|P63044\|VAMP2_MOUSE | Yes | M:L | 1 | 25 |
| SEAGAEGGTVQJGCQDEPK | 2 | 5.02E-09 | sp\|P51830\|ADCY9_MOUSE | Yes | K:T | 640 | 652 |
| SKDHAEDKLQNJDSAGK | 1 | 3.75E-11 | sp\|Q8VE97\|SRSF4_MOUSE | Yes | K:A | 269 | 281 |
| SPASTSSVJGTPGSQLSTPR | 1 | 2.54E-09 | sp\|Q9JLM8\|DCLK1_MOUSE | Yes | K:S | 306 | 315 |
| SPQSJGTSGVEQICVDVR | 1 | 4.68E-22 | sp\|P35803\|GPM6B_MOUSE | Yes | K:Q | 212 | 217 |
| SSEVETSEGVDESEKNSMAPEKEJHTK | 1 | 8.04E-09 | sp\|O70252\|HMOX2_MOUSE | Yes | M:M | 1 | 25 |
| TDSTSSJSTGPSGR | 1 | 1.09E-07 | sp\|Q3UNH4\|GRIN1_MOUSE | Yes | K:A | 342 | 349 |
| TEJSTSAPAAKPK | 2 | 2.95E-08 | sp\|P10922\|H10_MOUSE | Yes | M:R | 1 | 4 |
| TNJVSEHEDTDKYR | 1 | 2.63E-09 | sp\|Q9JIF7\|COPB_MOUSE | Yes | K:Q | 366 | 369 |
| TPTPLMAJDSTETSEAGEEEEDH EGDSENKER | 2 | 6.43E-19 | sp\|Q80TB8\|VAT1L_MOUSE | Yes | K:M | 380 | 388 |
| TVPNDATQTKEJESKA | 1 | 4.54E-07 | sp\|P13595\|NCAM1_MOUSE | Yes | K:- | 1099 | 1111 |
| TVTNAVVTVPAYFJDSQR | 1 | 4.09E-11 | sp\|P63017\|HSP7C_MOUSE | Yes | K:Q | 137 | 151 |
| VAEIPFJSTNK | 1 | 1.10E-08 | sp\|Q6PIC6\|AT1A3_MOUSE | No | K:Y | 466 | 473 |
| VDJSSLTGESEPQTR | 3 | 1.58E-18 | sp\|Q64436\|ATP4A_MOUSE | No | K:S | 221 | 224 |
| VFJGSGKPIDR | 1 | 4.61E-07 | sp\|P62814\|VATB2_MOUSE | Yes | R:G | 130 | 133 |
| VINAAHSFYJGTTTLPISDEDRTPR | 1 | 3.07E-08 | sp\|Q3THK7\|GUAA_MOUSE | Yes | K:K | 295 | 305 |
| VLTLAJFTTKDEGDYFCELQVS GANPMSSJK | 1 | 8.34E-11 | sp\|P01831\|THY1_MOUSE | Yes | K:S | 88 | 94 |
| VNDGVCDCCDGTDEYNSGTVCEJTCR | 1 | 2.82E-08 | sp\|O08795\|GLU2B_MOUSE | Yes | R:E | 91 | 114 |
| YCAAPTEPVIHJGSQGTGTJGSEISDSD YQAEYPDEYHGEYPDDYPR | 1 | 7.55E-08 | sp\|Q9DAW9\|CNN3_MOUSE | Yes | K:E | 271 | 283 |
| JLSIQGVRWWLQWLGIJFSLFVVLFFLT TPSIIMSTMDKFJVTK | 1 | 5.14E-02 | sp\|Q91YT8\|CSCL1_MOUSE | Yes | K:P | 408 | 409 |
| NCEDMDECRTPJMCPNGMCINE DGSFKCICK | 1 | 1.26E-02 | sp\|Q61554\|FBN1_MOUSE | Yes | K:P | 568 | 580 |
| SLGQNPTEAELQGMVNEIDKDGJGT VDFPEFLTMMSR | 2 | 2.03E-04 | sp\|Q9D6P8\|CALL3_MOUSE | Yes | R:K | 38 | 61 |
| VGVTGSMCDKCQDGHYGFGKT GCLPCQCNJR | 1 | 1.16E-03 | sp\|Q8BH27\|MEGF9_MOUSE | Yes | K:S | 273 | 303 |
| VTPRATMYTFLPDJFSPAK | 1 | 1.29E-03 | sp\|Q69ZQ1\|K1161_MOUSE | Yes | K:P | 27 | 41 |
| AGCEMLQRHQGJITAEVMMGILR | 13 | 2.27E-04 | sp\|Q8VCA8\|SCRN2_MOUSE | Yes | R:D | 247 | 259 |
| DEGDYFCELQVSGANPMSSJK | 4 | 3.25E-23 | sp\|P01831\|THY1_MOUSE | Yes | K:S | 98 | 118 |
| DGEAFEMJGTEDGR | 2 | 1.18E-13 | sp\|P70232\|NCHL1_MOUSE | Yes | K:I | 554 | 562 |
| EEJATIATYPEFGVLDLK | 1 | 4.66E-05 | sp\|P97370\|AT1B3_MOUSE | Yes | K:Y | 194 | 197 |
| EJGTTCLMAEFAAK | 1 | 1.71E-09 | sp\|Q9D387\|LAMP5_MOUSE | Yes | R:F | 51 | 53 |
| FKLDWLGJCSGLNDDSYGYR | 3 | 4.10E-07 | sp\|P14094\|AT1B1_MOUSE | Yes | R:E | 150 | 158 |
| FLIPJASQPESK | 1 | 6.75E-06 | sp\|P63101\|1433Z_MOUSE | Yes | K:V | 103 | 108 |
| FQLLJFSSSELK | 2 | 1.30E-14 | sp\|Q8R5M8\|CADM1_MOUSE | Yes | R:V | 99 | 104 |
| GDTHTQILEGLQFJLTQTSEADIHK | 31 | 9.79E-17 | sp\|P07758\|A1AT1_MOUSE | No | K:S | 87 | 101 |
| GETASLLCJISVR | 2 | 3.30E-10 | sp\|Q8R366\|IGSF8_MOUSE | Yes | R:G | 452 | 461 |
| GKTFMQDYJECDMKEDDCAPGTCR | 1 | 4.17E-03 | sp\|Q5DID3\|UROL1_MOUSE | Yes | R:N | 894 | 903 |
| JGTVAVVEPECEK | 1 | 2.20E-08 | sp\|Q8BJI1\|S6A17_MOUSE | Yes | R:S | 185 | 186 |
| JTTLVEGAJLCLDLR | 2 | 1.31E-13 | sp\|P35802\|GPM6A_MOUSE | Yes | R:Q | 163 | 164 |
| JVSYJISSTVR | 1 | 1.67E-05 | sp\|P97797\|SHPS1_MOUSE | Yes | K:V | 204 | 205 |
| LAWEGJETVTTR | 13 | 2.31E-18 | sp\|Q91V14\|S12A5_MOUSE | Yes | K:L | 327 | 333 |
| LLAJSSMLGEGQVLR | 1 | 1.17E-09 | sp\|Q4V9Z5\|SE6L2_MOUSE | Yes | R:S | 243 | 247 |
| LLJATCDEYFTR | 1 | 2.45E-14 | sp\|Q91V14\|S12A5_MOUSE | Yes | R:N | 348 | 351 |
| LSHLPVASSASTVLSTJGTG AMJFTVGAPASLNGQCEWL PRLQNGMVPSQYNPLR | 1 | 6.20E-03 | sp\|Q01705\|NOTC1_MOUSE | Yes | R:P | 2262 | 2279 |
| NTDGTYJYTSLFLVJSSAHR | 2 | 2.17E-08 | sp\|P97797\|SHPS1_MOUSE | Yes | K:E | 305 | 312 |
| NVETJJSTVLIEGK | 2 | 4.29E-10 | sp\|Q9Z218\|DPP6_MOUSE | Yes | R:K | 107 | 112 |
| PGVTAJSSLNGRHSRMGSEQVLMR | 1 | 6.97E-03 | sp\|Q3TTA7\|CBLB_MOUSE | Yes | K:K | 615 | 621 |
| QELJDSLQVAER | 2 | 3.78E-17 | sp\|Q06890\|CLUS_MOUSE | Yes | R:L | 323 | 327 |
| SEMMYGJETELK | 2 | 1.40E-05 | sp\|B9EKR1\|PRPTZ_MOUSE | Yes | K:M | 1051 | 1058 |
| VINFYAGAJQSMJVTCVGK | 5 | 3.41E-09 | sp\|P14231\|AT1B2_MOUSE | Yes | R:R | 184 | 193 |
| VPIQSJETLLGAVINJVSEAMETLTR | 2 | 6.65E-18 | sp\|P56564\|EAA1_MOUSE | Yes | K:I | 200 | 206 |
| VSLTJVSISDEGR | 2 | 8.07E-20 | sp\|Q8R5M8\|CADM1_MOUSE | Yes | K:Y | 111 | 116 |
| YLQPLLAVQFTJLTVDTEIR | 56 | 5.77E-22 | sp\|P14094\|AT1B1_MOUSE | Yes | K:V | 254 | 266 |
| YVALGDLVILITFGPLAVMFAYAVQV GSLAIFPLIYAIPLALSTEAILHSJNTR | 1 | 8.23E-03 | sp\|Q9DC60\|UBIA1_MOUSE | Yes | K:D | 179 | 230 |

MS/MS without PNGase F treatment. False positives were only assigned if the deamidated Asn was found as part of an N-linked sequon N-!P-[S|T|C] (where !P is not proline).
