# Supplementary material for: In-depth mapping of the mouse brain N-glycoproteome reveals widespread N-glycosylation of diverse brain proteins
Source: Oncotarget. 2016 May 31;7(25):38796–809. doi: 10.18632/oncotarget.9737 (PMC5122430; doi:10.18632/oncotarget.9737)
Supplement: Supplementary file 4 [file oncotarget-07-38796-s004.docx]

| Supplementary Table S5: Gene Ontology enrichment analysis of identified N-glycoproteins in mouse brain | | | | | | | |
| --- | --- | --- | --- | --- | --- | --- | --- |
| **Cellular compartments** | | | | | | | |
| Annotation Cluster 1 | Enrichment Score: 15.02 | Count | P_Value | Fold Change | Bonferroni | Benjamini | FDR |
| GOTERM_CC_FAT | synapse | 142 | 3.70E-24 | 2.30E + 00 | 2.10E-21 | 1.10E-21 | 5.40E-21 |
| GOTERM_CC_FAT | synapse part | 98 | 2.90E-18 | 2.30E+00 | 1.60E-15 | 3.30E-16 | 4.20E-15 |
| GOTERM_CC_FAT | postsynaptic membrane | 60 | 4.40E-12 | 2.40E+00 | 2.50E-09 | 2.10E-10 | 6.50E-09 |
| GOTERM_CC_FAT | cell junction | 143 | 1.80E-08 | 1.50E+00 | 1.00E-05 | 4.10E-07 | 2.60E-05 |
| Annotation Cluster 2 | Enrichment Score: 13.74 | Count | P_Value | Fold Change | Bonferroni | Benjamini | FDR |
| GOTERM_CC_FAT | lysosome | 82 | 3.50E-15 | 2.30E+00 | 2.00E-12 | 2.50E-13 | 5.10E-12 |
| GOTERM_CC_FAT | lytic vacuole | 82 | 5.10E-15 | 2.30E+00 | 2.90E-12 | 3.30E-13 | 7.50E-12 |
| GOTERM_CC_FAT | vacuole | 86 | 3.50E-13 | 2.10E+00 | 2.00E-10 | 2.00E-11 | 5.10E-10 |
| Annotation Cluster 3 | Enrichment Score: 9.76 | Count | P_Value | Fold Change | Bonferroni | Benjamini | FDR |
| GOTERM_CC_FAT | membrane fraction | 161 | 1.00E-10 | 1.60E+00 | 5.70E-08 | 3.80E-09 | 1.50E-07 |
| GOTERM_CC_FAT | cell fraction | 181 | 2.10E-10 | 1.50E+00 | 1.20E-07 | 6.60E-09 | 3.00E-07 |
| GOTERM_CC_FAT | insoluble fraction | 164 | 2.60E-10 | 1.60E+00 | 1.50E-07 | 7.80E-09 | 3.80E-07 |
| Annotation Cluster 4 | Enrichment Score: 9.37 | Count | P_Value | Fold Change | Bonferroni | Benjamini | FDR |
| GOTERM_CC_FAT | proteinaceous extracellular matrix | 105 | 2.70E-10 | 1.80E+00 | 1.60E-07 | 7.80E-09 | 4.00E-07 |
| GOTERM_CC_FAT | extracellular matrix | 108 | 3.10E-10 | 1.80E+00 | 1.80E-07 | 8.60E-09 | 4.60E-07 |
| GOTERM_CC_FAT | extracellular region part | 221 | 9.10E-10 | 1.50E+00 | 5.20E-07 | 2.40E-08 | 1.30E-06 |
| Annotation Cluster 5 | Enrichment Score: 7.68 | Count | P_Value | Fold Change | Bonferroni | Benjamini | FDR |
| GOTERM_CC_FAT | membrane-bounded vesicle | 131 | 1.50E-08 | 1.60E+00 | 8.50E-06 | 3.70E-07 | 2.20E-05 |
| GOTERM_CC_FAT | vesicle | 155 | 1.70E-08 | 1.50E+00 | 9.70E-06 | 4.00E-07 | 2.50E-05 |
| GOTERM_CC_FAT | cytoplasmic membrane-bounded vesicle | 129 | 2.10E-08 | 1.60E+00 | 1.20E-05 | 4.60E-07 | 3.00E-05 |
| GOTERM_CC_FAT | cytoplasmic vesicle | 151 | 3.70E-08 | 1.50E+00 | 2.10E-05 | 7.60E-07 | 5.40E-05 |
| Annotation Cluster 6 | Enrichment Score: 4.48 | Count | P_Value | Fold Change | Bonferroni | Benjamini | FDR |
| GOTERM_CC_FAT | intrinsic to organelle membrane | 45 | 1.20E-06 | 2.00E+00 | 6.90E-04 | 1.70E-05 | 1.80E-03 |
| GOTERM_CC_FAT | integral to Golgi membrane | 18 | 2.50E-05 | 2.90E+00 | 1.50E-02 | 2.90E-04 | 3.70E-02 |
| GOTERM_CC_FAT | intrinsic to Golgi membrane | 18 | 2.50E-05 | 2.90E+00 | 1.50E-02 | 2.90E-04 | 3.70E-02 |
| GOTERM_CC_FAT | integral to organelle membrane | 35 | 3.80E-05 | 2.00E+00 | 2.20E-02 | 4.20E-04 | 5.60E-02 |
| GOTERM_CC_FAT | Golgi membrane | 43 | 1.40E-03 | 1.60E+00 | 5.50E-01 | 1.30E-02 | 2.00E+00 |
| Annotation Cluster 7 | Enrichment Score: 3.36 | Count | P_Value | Fold Change | Bonferroni | Benjamini | FDR |
| GOTERM_CC_FAT | endoplasmic reticulum part | 80 | 1.20E-07 | 1.80E+00 | 6.90E-05 | 2.10E-06 | 1.80E-04 |
| GOTERM_CC_FAT | nuclear envelope-endoplasmic reticulum network | 46 | 6.00E-03 | 1.50E+00 | 9.70E-01 | 5.00E-02 | 8.50E+00 |
| GOTERM_CC_FAT | endoplasmic reticulum membrane | 44 | 6.40E-03 | 1.50E+00 | 9.70E-01 | 5.20E-02 | 9.00E+00 |
| GOTERM_CC_FAT | intrinsic to endoplasmic reticulum membrane | 19 | 8.20E-03 | 1.90E+00 | 9.90E-01 | 6.40E-02 | 1.10E+01 |
| Annotation Cluster 8 | Enrichment Score: 3.01 | Count | P_Value | Fold Change | Bonferroni | Benjamini | FDR |
| GOTERM_CC_FAT | synaptic vesicle membrane | 14 | 5.30E-06 | 3.70E+00 | 3.00E-03 | 6.60E-05 | 7.70E-03 |
| GOTERM_CC_FAT | synaptic vesicle | 30 | 1.20E-05 | 2.20E+00 | 7.00E-03 | 1.40E-04 | 1.80E-02 |
| GOTERM_CC_FAT | clathrin-coated vesicle | 38 | 1.00E-04 | 1.90E+00 | 5.80E-02 | 1.10E-03 | 1.50E-01 |
| GOTERM_CC_FAT | coated vesicle | 43 | 1.30E-04 | 1.80E+00 | 7.40E-02 | 1.40E-03 | 2.00E-01 |
| GOTERM_CC_FAT | clathrin coated vesicle membrane | 15 | 6.00E-03 | 2.10E+00 | 9.70E-01 | 5.00E-02 | 8.40E+00 |
| GOTERM_CC_FAT | cytoplasmic vesicle part | 29 | 1.40E-02 | 1.60E+00 | 1.00E+00 | 1.10E-01 | 1.90E+01 |
| GOTERM_CC_FAT | vesicle membrane | 29 | 1.90E-02 | 1.50E+00 | 1.00E+00 | 1.30E-01 | 2.40E+01 |
| GOTERM_CC_FAT | coated vesicle membrane | 18 | 2.20E-02 | 1.70E+00 | 1.00E+00 | 1.50E-01 | 2.80E+01 |
| GOTERM_CC_FAT | cytoplasmic vesicle membrane | 26 | 2.70E-02 | 1.50E+00 | 1.00E+00 | 1.70E-01 | 3.30E+01 |
| Annotation Cluster 9 | Enrichment Score: 2.67 | Count | P_Value | Fold Change | Bonferroni | Benjamini | FDR |
| GOTERM_CC_FAT | basal lamina | 10 | 1.40E-04 | 3.90E+00 | 8.00E-02 | 1.50E-03 | 2.10E-01 |
| GOTERM_CC_FAT | laminin complex | 6 | 4.30E-03 | 4.40E+00 | 9.20E-01 | 3.80E-02 | 6.20E+00 |
| GOTERM_CC_FAT | laminin-1 complex | 5 | 1.60E-02 | 4.20E+00 | 1.00E+00 | 1.20E-01 | 2.10E+01 |
| Annotation Cluster 10 | Enrichment Score: 1.61 | Count | P_Value | Fold Change | Bonferroni | Benjamini | FDR |
| GOTERM_CC_FAT | axon part | 11 | 1.50E-02 | 2.20E+00 | 1.00E+00 | 1.20E-01 | 2.00E+01 |
| GOTERM_CC_FAT | axolemma | 5 | 1.60E-02 | 4.20E+00 | 1.00E+00 | 1.20E-01 | 2.10E+01 |
| GOTERM_CC_FAT | neuron projection membrane | 5 | 3.10E-02 | 3.60E+00 | 1.00E+00 | 1.90E-01 | 3.70E+01 |
| GOTERM_CC_FAT | leading edge membrane | 6 | 4.70E-02 | 2.80E+00 | 1.00E+00 | 2.70E-01 | 5.10E+01 |
| **Molecular function** |  |  |  |  |  |  |  |
| Annotation Cluster 1 | Enrichment Score: 9.91 | Count | P_Value | Fold Change | Bonferroni | Benjamini | FDR |
| GOTERM_MF_FAT | gated channel activity | 106 | 1.30E-18 | 2.30E+00 | 1.90E-15 | 6.40E-16 | 2.20E-15 |
| GOTERM_MF_FAT | ion channel activity | 121 | 9.30E-18 | 2.20E+00 | 1.30E-14 | 3.40E-15 | 1.50E-14 |
| GOTERM_MF_FAT | substrate specific channel activity | 123 | 1.80E-17 | 2.10E+00 | 2.60E-14 | 5.20E-15 | 3.00E-14 |
| GOTERM_MF_FAT | passive transmembrane transporter activity | 124 | 2.20E-17 | 2.10E+00 | 3.10E-14 | 5.20E-15 | 3.60E-14 |
| GOTERM_MF_FAT | channel activity | 124 | 2.20E-17 | 2.10E+00 | 3.10E-14 | 5.20E-15 | 3.60E-14 |
| GOTERM_MF_FAT | metal ion transmembrane transporter activity | 88 | 1.40E-09 | 1.90E+00 | 2.10E-06 | 1.00E-07 | 2.40E-06 |
| GOTERM_MF_FAT | voltage-gated channel activity | 62 | 2.10E-09 | 2.20E+00 | 3.00E-06 | 1.40E-07 | 3.40E-06 |
| GOTERM_MF_FAT | voltage-gated ion channel activity | 62 | 2.10E-09 | 2.20E+00 | 3.00E-06 | 1.40E-07 | 3.40E-06 |
| GOTERM_MF_FAT | cation channel activity | 75 | 3.10E-08 | 1.90E+00 | 4.40E-05 | 1.70E-06 | 5.10E-05 |
| GOTERM_MF_FAT | potassium ion binding | 44 | 5.50E-08 | 2.30E+00 | 7.90E-05 | 2.80E-06 | 9.00E-05 |
| GOTERM_MF_FAT | calcium channel activity | 30 | 1.70E-07 | 2.70E+00 | 2.40E-04 | 8.00E-06 | 2.80E-04 |
| GOTERM_MF_FAT | voltage-gated cation channel activity | 36 | 7.90E-04 | 1.80E+00 | 6.80E-01 | 1.40E-02 | 1.30E+00 |
| GOTERM_MF_FAT | potassium channel activity | 31 | 2.10E-02 | 1.50E+00 | 1.00E+00 | 2.10E-01 | 2.90E+01 |
| GOTERM_MF_FAT | voltage-gated potassium channel activity | 23 | 6.60E-02 | 1.40E+00 | 1.00E+00 | 4.70E-01 | 6.80E+01 |
| Annotation Cluster 2 | Enrichment Score: 8.92 | Count | P_Value | Fold Change | Bonferroni | Benjamini | FDR |
| GOTERM_MF_FAT | carbohydrate binding | 98 | 4.80E-11 | 1.90E+00 | 6.90E-08 | 5.70E-09 | 7.90E-08 |
| GOTERM_MF_FAT | polysaccharide binding | 52 | 8.00E-11 | 2.50E+00 | 1.20E-07 | 8.20E-09 | 1.30E-07 |
| GOTERM_MF_FAT | pattern binding | 52 | 8.00E-11 | 2.50E+00 | 1.20E-07 | 8.20E-09 | 1.30E-07 |
| GOTERM_MF_FAT | glycosaminoglycan binding | 47 | 4.10E-10 | 2.60E+00 | 5.90E-07 | 3.30E-08 | 6.70E-07 |
| GOTERM_MF_FAT | heparin binding | 30 | 2.00E-05 | 2.20E+00 | 2.80E-02 | 5.50E-04 | 3.30E-02 |
| Annotation Cluster 3 | Enrichment Score: 7.68 | Count | P_Value | Fold Change | Bonferroni | Benjamini | FDR |
| GOTERM_MF_FAT | anion transmembrane transporter activity | 60 | 2.60E-15 | 2.90E+00 | 3.70E-12 | 5.30E-13 | 4.20E-12 |
| GOTERM_MF_FAT | anion channel activity | 35 | 1.30E-10 | 3.20E+00 | 1.90E-07 | 1.20E-08 | 2.10E-07 |
| GOTERM_MF_FAT | chloride ion binding | 34 | 4.00E-10 | 3.10E+00 | 5.80E-07 | 3.40E-08 | 6.70E-07 |
| GOTERM_MF_FAT | anion binding | 34 | 1.00E-09 | 3.00E+00 | 1.50E-06 | 8.00E-08 | 1.70E-06 |
| GOTERM_MF_FAT | chloride channel activity | 31 | 7.10E-09 | 3.00E+00 | 1.00E-05 | 4.40E-07 | 1.20E-05 |
| GOTERM_MF_FAT | GABA-A receptor activity | 13 | 2.00E-06 | 4.50E+00 | 2.90E-03 | 7.40E-05 | 3.40E-03 |
| GOTERM_MF_FAT | GABA receptor activity | 17 | 2.80E-06 | 3.50E+00 | 4.00E-03 | 9.90E-05 | 4.60E-03 |
| GOTERM_MF_FAT | neurotransmitter receptor activity | 28 | 3.60E-04 | 2.00E+00 | 4.10E-01 | 7.30E-03 | 6.00E-01 |
| GOTERM_MF_FAT | neurotransmitter binding | 28 | 3.60E-04 | 2.00E+00 | 4.10E-01 | 7.30E-03 | 6.00E-01 |
| Annotation Cluster 4 | Enrichment Score: 7.47 | Count | P_Value | Fold Change | Bonferroni | Benjamini | FDR |
| GOTERM_MF_FAT | extracellular ligand-gated ion channel activity | 36 | 4.50E-12 | 3.40E+00 | 6.50E-09 | 8.20E-10 | 7.50E-09 |
| GOTERM_MF_FAT | glutamate receptor activity | 26 | 7.80E-12 | 4.30E+00 | 1.10E-08 | 1.10E-09 | 1.30E-08 |
| GOTERM_MF_FAT | ionotropic glutamate receptor activity | 15 | 1.20E-08 | 5.20E+00 | 1.70E-05 | 7.10E-07 | 1.90E-05 |
| GOTERM_MF_FAT | extracellular-glutamate-gated ion channel activity | 15 | 1.20E-08 | 5.20E+00 | 1.70E-05 | 7.10E-07 | 1.90E-05 |
| GOTERM_MF_FAT | ligand-gated ion channel activity | 41 | 5.20E-08 | 2.40E+00 | 7.50E-05 | 2.80E-06 | 8.60E-05 |
| GOTERM_MF_FAT | ligand-gated channel activity | 41 | 5.20E-08 | 2.40E+00 | 7.50E-05 | 2.80E-06 | 8.60E-05 |
| GOTERM_MF_FAT | neurotransmitter receptor activity | 28 | 3.60E-04 | 2.00E+00 | 4.10E-01 | 7.30E-03 | 6.00E-01 |
| GOTERM_MF_FAT | neurotransmitter binding | 28 | 3.60E-04 | 2.00E+00 | 4.10E-01 | 7.30E-03 | 6.00E-01 |
| Annotation Cluster 5 | Enrichment Score: 5.78 | Count | P_Value | Fold Change | Bonferroni | Benjamini | FDR |
| GOTERM_MF_FAT | adenyl ribonucleotide binding | 305 | 2.10E-07 | 1.30E+00 | 3.00E-04 | 9.60E-06 | 3.40E-04 |
| GOTERM_MF_FAT | adenyl nucleotide binding | 318 | 2.50E-07 | 1.30E+00 | 3.60E-04 | 1.10E-05 | 4.10E-04 |
| GOTERM_MF_FAT | ATP binding | 301 | 2.90E-07 | 1.30E+00 | 4.20E-04 | 1.30E-05 | 4.80E-04 |
| GOTERM_MF_FAT | nucleoside binding | 321 | 3.60E-07 | 1.30E+00 | 5.20E-04 | 1.50E-05 | 6.00E-04 |
| GOTERM_MF_FAT | purine nucleoside binding | 319 | 3.90E-07 | 1.30E+00 | 5.70E-04 | 1.60E-05 | 6.50E-04 |
| GOTERM_MF_FAT | protein kinase activity | 138 | 1.20E-06 | 1.50E+00 | 1.70E-03 | 4.50E-05 | 2.00E-03 |
| GOTERM_MF_FAT | ribonucleotide binding | 352 | 1.30E-05 | 1.20E+00 | 1.80E-02 | 3.80E-04 | 2.10E-02 |
| GOTERM_MF_FAT | purine ribonucleotide binding | 352 | 1.30E-05 | 1.20E+00 | 1.80E-02 | 3.80E-04 | 2.10E-02 |
| GOTERM_MF_FAT | purine nucleotide binding | 365 | 1.30E-05 | 1.20E+00 | 1.90E-02 | 4.00E-04 | 2.20E-02 |
| GOTERM_MF_FAT | nucleotide binding | 416 | 3.20E-05 | 1.20E+00 | 4.50E-02 | 8.60E-04 | 5.30E-02 |
| Annotation Cluster 6 | Enrichment Score: 4.85 | Count | P_Value | Fold Change | Bonferroni | Benjamini | FDR |
| GOTERM_MF_FAT | ion binding | 759 | 6.90E-11 | 1.20E+00 | 9.90E-08 | 7.60E-09 | 1.10E-07 |
| GOTERM_MF_FAT | cation binding | 732 | 2.20E-08 | 1.20E+00 | 3.10E-05 | 1.30E-06 | 3.60E-05 |
| GOTERM_MF_FAT | metal ion binding | 716 | 3.70E-07 | 1.20E+00 | 5.30E-04 | 1.50E-05 | 6.10E-04 |
| GOTERM_MF_FAT | transition metal ion binding | 374 | 1.00E+00 | 8.90E-01 | 1.00E+00 | 1.00E+00 | 1.00E+02 |
| GOTERM_MF_FAT | zinc ion binding | 291 | 1.00E+00 | 8.60E-01 | 1.00E+00 | 1.00E+00 | 1.00E+02 |
| Annotation Cluster 7 | Enrichment Score: 4.38 | Count | P_Value | Fold Change | Bonferroni | Benjamini | FDR |
| GOTERM_MF_FAT | symporter activity | 54 | 2.40E-11 | 2.50E+00 | 3.50E-08 | 3.20E-09 | 4.00E-08 |
| GOTERM_MF_FAT | solute:cation symporter activity | 32 | 7.10E-08 | 2.70E+00 | 1.00E-04 | 3.50E-06 | 1.20E-04 |
| GOTERM_MF_FAT | neurotransmitter transporter activity | 14 | 5.40E-06 | 4.00E+00 | 7.80E-03 | 1.90E-04 | 8.90E-03 |
| GOTERM_MF_FAT | neurotransmitter:sodium symporter activity | 13 | 1.00E-05 | 4.00E+00 | 1.40E-02 | 3.20E-04 | 1.70E-02 |
| GOTERM_MF_FAT | amine transmembrane transporter activity | 25 | 2.00E-05 | 2.50E+00 | 2.80E-02 | 5.60E-04 | 3.30E-02 |
| GOTERM_MF_FAT | sodium:amino acid symporter activity | 7 | 1.00E-04 | 6.20E+00 | 1.40E-01 | 2.60E-03 | 1.70E-01 |
| GOTERM_MF_FAT | solute:sodium symporter activity | 19 | 1.40E-04 | 2.60E+00 | 1.80E-01 | 3.20E-03 | 2.30E-01 |
| GOTERM_MF_FAT | amino acid transmembrane transporter activity | 20 | 1.50E-04 | 2.50E+00 | 1.90E-01 | 3.30E-03 | 2.40E-01 |
| GOTERM_MF_FAT | L-amino acid transmembrane transporter activity | 14 | 2.40E-04 | 3.00E+00 | 3.00E-01 | 5.10E-03 | 4.00E-01 |
| GOTERM_MF_FAT | organic acid:sodium symporter activity | 11 | 1.10E-03 | 3.10E+00 | 8.00E-01 | 1.90E-02 | 1.80E+00 |
| GOTERM_MF_FAT | cation:amino acid symporter activity | 7 | 2.00E-03 | 4.40E+00 | 9.40E-01 | 3.20E-02 | 3.20E+00 |
| GOTERM_MF_FAT | gamma-aminobutyric acid:sodium symporter activity | 4 | 1.50E-02 | 6.20E+00 | 1.00E+00 | 1.60E-01 | 2.20E+01 |
| GOTERM_MF_FAT | L-gamma-aminobutyric acid transmembrane transporter activity | 4 | 3.20E-02 | 5.00E+00 | 1.00E+00 | 2.90E-01 | 4.20E+01 |
| Annotation Cluster 8 | Enrichment Score: 3.88 | Count | P_Value | Fold Change | Bonferroni | Benjamini | FDR |
| GOTERM_MF_FAT | metallopeptidase activity | 57 | 1.30E-06 | 1.90E+00 | 1.80E-03 | 4.70E-05 | 2.10E-03 |
| GOTERM_MF_FAT | peptidase activity, acting on L-amino acid peptides | 132 | 1.10E-04 | 1.40E+00 | 1.50E-01 | 2.70E-03 | 1.80E-01 |
| GOTERM_MF_FAT | peptidase activity | 134 | 3.40E-04 | 1.30E+00 | 3.90E-01 | 6.90E-03 | 5.60E-01 |
| GOTERM_MF_FAT | endopeptidase activity | 88 | 6.50E-03 | 1.30E+00 | 1.00E+00 | 8.50E-02 | 1.00E+01 |
| Annotation Cluster 9 | Enrichment Score: 3.86 | Count | P_Value | Fold Change | Bonferroni | Benjamini | FDR |
| GOTERM_MF_FAT | low-density lipoprotein binding | 10 | 2.70E-05 | 4.80E+00 | 3.80E-02 | 7.30E-04 | 4.50E-02 |
| GOTERM_MF_FAT | low-density lipoprotein receptor activity | 8 | 7.30E-05 | 5.50E+00 | 1.00E-01 | 1.80E-03 | 1.20E-01 |
| GOTERM_MF_FAT | lipoprotein receptor activity | 9 | 2.60E-04 | 4.30E+00 | 3.10E-01 | 5.50E-03 | 4.30E-01 |
| GOTERM_MF_FAT | lipoprotein binding | 11 | 7.10E-04 | 3.30E+00 | 6.40E-01 | 1.30E-02 | 1.20E+00 |
| Annotation Cluster 10 | Enrichment Score: 2.69 | Count | P_Value | Fold Change | Bonferroni | Benjamini | FDR |
| GOTERM_MF_FAT | endopeptidase inhibitor activity | 44 | 3.70E-04 | 1.70E+00 | 4.10E-01 | 7.40E-03 | 6.10E-01 |
| GOTERM_MF_FAT | peptidase inhibitor activity | 45 | 1.40E-03 | 1.60E+00 | 8.70E-01 | 2.40E-02 | 2.30E+00 |
| GOTERM_MF_FAT | serine-type endopeptidase inhibitor activity | 32 | 2.00E-03 | 1.70E+00 | 9.40E-01 | 3.20E-02 | 3.20E+00 |
| GOTERM_MF_FAT | enzyme inhibitor activity | 53 | 1.60E-02 | 1.40E+00 | 1.00E+00 | 1.80E-01 | 2.40E+01 |
| **Biological processes** |  |  |  |  |  |  |  |
| Annotation Cluster 1 | Enrichment Score: 16.02 | Count | P_Value | Fold Change | Bonferroni | Benjamini | FDR |
| GOTERM_BP_FAT | ion transport | 228 | 1.30E-25 | 1.90E+00 | 5.40E-22 | 1.80E-22 | 2.50E-22 |
| GOTERM_BP_FAT | metal ion transport | 149 | 3.70E-19 | 2.00E+00 | 1.50E-15 | 3.00E-16 | 7.00E-16 |
| GOTERM_BP_FAT | cation transport | 164 | 2.80E-18 | 1.90E+00 | 1.10E-14 | 1.60E-15 | 5.10E-15 |
| GOTERM_BP_FAT | di-, tri-valent inorganic cation transport | 64 | 4.80E-12 | 2.40E+00 | 1.90E-08 | 9.20E-10 | 8.90E-09 |
| GOTERM_BP_FAT | monovalent inorganic cation transport | 90 | 1.30E-08 | 1.80E+00 | 5.20E-05 | 9.60E-07 | 2.40E-05 |
| Annotation Cluster 2 | Enrichment Score: 13.45 | Count | P_Value | Fold Change | Bonferroni | Benjamini | FDR |
| GOTERM_BP_FAT | transmission of nerve impulse | 99 | 6.70E-22 | 2.60E+00 | 2.70E-18 | 6.80E-19 | 1.20E-18 |
| GOTERM_BP_FAT | synaptic transmission | 80 | 1.20E-18 | 2.70E+00 | 4.80E-15 | 8.00E-16 | 2.20E-15 |
| GOTERM_BP_FAT | cell-cell signaling | 104 | 1.90E-15 | 2.20E+00 | 7.70E-12 | 9.60E-13 | 3.50E-12 |
| GOTERM_BP_FAT | neurological system process | 183 | 1.00E+00 | 6.60E-01 | 1.00E+00 | 1.00E+00 | 1.00E+02 |
| Annotation Cluster 3 | Enrichment Score: 11.05 | Count | P_Value | Fold Change | Bonferroni | Benjamini | FDR |
| GOTERM_BP_FAT | cell morphogenesis involved in differentiation | 83 | 6.30E-15 | 2.40E+00 | 2.50E-11 | 2.80E-12 | 1.20E-11 |
| GOTERM_BP_FAT | cell morphogenesis involved in neuron differentiation | 74 | 2.20E-14 | 2.50E+00 | 8.70E-11 | 8.70E-12 | 4.00E-11 |
| GOTERM_BP_FAT | neuron projection morphogenesis | 70 | 4.10E-13 | 2.40E+00 | 1.70E-09 | 1.20E-10 | 7.70E-10 |
| GOTERM_BP_FAT | axonogenesis | 66 | 7.90E-13 | 2.40E+00 | 3.20E-09 | 2.00E-10 | 1.50E-09 |
| GOTERM_BP_FAT | cellular component morphogenesis | 112 | 1.20E-12 | 1.90E+00 | 5.00E-09 | 2.60E-10 | 2.30E-09 |
| GOTERM_BP_FAT | cell morphogenesis | 102 | 1.40E-12 | 2.00E+00 | 5.70E-09 | 2.90E-10 | 2.60E-09 |
| GOTERM_BP_FAT | cell projection organization | 103 | 4.90E-12 | 2.00E+00 | 2.00E-08 | 9.00E-10 | 9.10E-09 |
| GOTERM_BP_FAT | cell projection morphogenesis | 74 | 9.50E-12 | 2.20E+00 | 3.80E-08 | 1.50E-09 | 1.80E-08 |
| GOTERM_BP_FAT | neuron projection development | 78 | 9.60E-12 | 2.20E+00 | 3.90E-08 | 1.50E-09 | 1.80E-08 |
| GOTERM_BP_FAT | cell part morphogenesis | 75 | 4.60E-11 | 2.10E+00 | 1.90E-07 | 6.00E-09 | 8.60E-08 |
| GOTERM_BP_FAT | neuron development | 92 | 3.20E-10 | 1.90E+00 | 1.30E-06 | 3.50E-08 | 5.90E-07 |
| GOTERM_BP_FAT | neuron differentiation | 111 | 1.20E-08 | 1.70E+00 | 5.00E-05 | 9.40E-07 | 2.30E-05 |
| GOTERM_BP_FAT | axon guidance | 38 | 3.40E-07 | 2.30E+00 | 1.40E-03 | 2.20E-05 | 6.40E-04 |
| Annotation Cluster 4 | Enrichment Score: 10.24 | Count | P_Value | Fold Change | Bonferroni | Benjamini | FDR |
| GOTERM_BP_FAT | glycoprotein metabolic process | 64 | 3.50E-14 | 2.60E+00 | 1.40E-10 | 1.30E-11 | 6.50E-11 |
| GOTERM_BP_FAT | glycoprotein biosynthetic process | 53 | 1.10E-12 | 2.70E+00 | 4.30E-09 | 2.40E-10 | 2.00E-09 |
| GOTERM_BP_FAT | biopolymer glycosylation | 40 | 2.60E-09 | 2.70E+00 | 1.10E-05 | 2.30E-07 | 4.80E-06 |
| GOTERM_BP_FAT | protein amino acid glycosylation | 40 | 2.60E-09 | 2.70E+00 | 1.10E-05 | 2.30E-07 | 4.80E-06 |
| GOTERM_BP_FAT | glycosylation | 40 | 2.60E-09 | 2.70E+00 | 1.10E-05 | 2.30E-07 | 4.80E-06 |
| Annotation Cluster 5 | Enrichment Score: 9.6 | Count | P_Value | Fold Change | Bonferroni | Benjamini | FDR |
| GOTERM_BP_FAT | regulation of neurological system process | 50 | 1.40E-11 | 2.70E+00 | 5.90E-08 | 2.20E-09 | 2.70E-08 |
| GOTERM_BP_FAT | regulation of system process | 73 | 2.10E-11 | 2.20E+00 | 8.60E-08 | 3.10E-09 | 3.90E-08 |
| GOTERM_BP_FAT | regulation of transmission of nerve impulse | 47 | 8.70E-11 | 2.70E+00 | 3.50E-07 | 1.10E-08 | 1.60E-07 |
| GOTERM_BP_FAT | regulation of synaptic transmission | 45 | 8.80E-11 | 2.70E+00 | 3.60E-07 | 1.00E-08 | 1.60E-07 |
| GOTERM_BP_FAT | regulation of synaptic plasticity | 24 | 4.20E-07 | 3.00E+00 | 1.70E-03 | 2.60E-05 | 7.90E-04 |
| Annotation Cluster 6 | Enrichment Score: 9.05 | Count | P_Value | Fold Change | Bonferroni | Benjamini | FDR |
| GOTERM_BP_FAT | membrane invagination | 67 | 3.90E-10 | 2.20E+00 | 1.60E-06 | 4.00E-08 | 7.20E-07 |
| GOTERM_BP_FAT | endocytosis | 67 | 3.90E-10 | 2.20E+00 | 1.60E-06 | 4.00E-08 | 7.20E-07 |
| GOTERM_BP_FAT | membrane organization | 86 | 1.10E-09 | 1.90E+00 | 4.30E-06 | 1.00E-07 | 2.00E-06 |
| GOTERM_BP_FAT | vesicle-mediated transport | 127 | 3.90E-09 | 1.60E+00 | 1.60E-05 | 3.30E-07 | 7.30E-06 |
| Annotation Cluster 7 | Enrichment Score: 8.94 | Count | P_Value | Fold Change | Bonferroni | Benjamini | FDR |
| GOTERM_BP_FAT | cell motion | 119 | 6.50E-14 | 2.00E+00 | 2.60E-10 | 2.20E-11 | 1.20E-10 |
| GOTERM_BP_FAT | cell migration | 76 | 1.00E-08 | 1.90E+00 | 4.10E-05 | 8.00E-07 | 1.90E-05 |
| GOTERM_BP_FAT | localization of cell | 84 | 5.10E-08 | 1.80E+00 | 2.10E-04 | 3.50E-06 | 9.40E-05 |
| GOTERM_BP_FAT | cell motility | 84 | 5.10E-08 | 1.80E+00 | 2.10E-04 | 3.50E-06 | 9.40E-05 |
| Annotation Cluster 8 | Enrichment Score: 8.11 | Count | P_Value | Fold Change | Bonferroni | Benjamini | FDR |
| GOTERM_BP_FAT | regulation of cell motion | 46 | 3.30E-10 | 2.60E+00 | 1.40E-06 | 3.60E-08 | 6.20E-07 |
| GOTERM_BP_FAT | regulation of cell migration | 40 | 3.80E-09 | 2.60E+00 | 1.50E-05 | 3.30E-07 | 7.00E-06 |
| GOTERM_BP_FAT | regulation of locomotion | 41 | 3.80E-07 | 2.30E+00 | 1.50E-03 | 2.30E-05 | 7.00E-04 |
| Annotation Cluster 9 | Enrichment Score: 6.86 | Count | P_Value | Fold Change | Bonferroni | Benjamini | FDR |
| GOTERM_BP_FAT | phosphate metabolic process | 206 | 1.40E-08 | 1.40E+00 | 5.90E-05 | 1.10E-06 | 2.70E-05 |
| GOTERM_BP_FAT | phosphorus metabolic process | 206 | 1.40E-08 | 1.40E+00 | 5.90E-05 | 1.10E-06 | 2.70E-05 |
| GOTERM_BP_FAT | phosphorylation | 169 | 6.90E-07 | 1.40E+00 | 2.80E-03 | 3.90E-05 | 1.30E-03 |
| GOTERM_BP_FAT | protein amino acid phosphorylation | 151 | 2.50E-06 | 1.40E+00 | 1.00E-02 | 1.30E-04 | 4.70E-03 |
| Annotation Cluster 10 | Enrichment Score: 6.55 | Count | P_Value | Fold Change | Bonferroni | Benjamini | FDR |
| GOTERM_BP_FAT | locomotory behavior | 79 | 5.50E-10 | 2.00E+00 | 2.20E-06 | 5.60E-08 | 1.00E-06 |
| GOTERM_BP_FAT | adult behavior | 37 | 2.40E-07 | 2.40E+00 | 9.70E-04 | 1.60E-05 | 4.50E-04 |
| GOTERM_BP_FAT | adult locomotory behavior | 27 | 1.90E-06 | 2.60E+00 | 7.70E-03 | 1.00E-04 | 3.50E-03 |
| GOTERM_BP_FAT | adult walking behavior | 15 | 2.60E-05 | 3.40E+00 | 9.90E-02 | 1.00E-03 | 4.80E-02 |

GO enrichment analysis was performed using DAVID (http://david.abcc.ncifcrf.gov/). The *p* value was set at 0.05.
