# Supplementary material for: In-depth mapping of the mouse brain N-glycoproteome reveals widespread N-glycosylation of diverse brain proteins
Source: Oncotarget. 2016 May 31;7(25):38796–809. doi: 10.18632/oncotarget.9737 (PMC5122430; doi:10.18632/oncotarget.9737)
Supplement: Supplementary file 5 [file oncotarget-07-38796-s005.docx]

| Supplementary Table S6: All canonical pathways that the identified N-glycoproteins were significantly associated with | | | |
| --- | --- | --- | --- |
| **Canonical pathways** | **–log(*p*-value)** | **Ratio** | **Molecules** |
| Axonal Guidance Signaling | 32.30 | 42.60% | SLIT3,DPYSL2,KLC1,RAF1,TUBA1B,ADAMTS8,ECEL1,Adam26a/Adam26b,ARPC5,SEMA4F,NTN1,ADAMTS2,GNB1,VEGFA,NCK2,GNB4,PAK1,ITGA3,EPHB1,ECE2,ABLIM3,Adam24,PLCB1,PLXNB2,SRGAP2,PLXNB3,MYL3,ITGA4,EFNA2,ACTR2,SEMA5A,PTCH1,TUBB2A,ITGA5,L1CAM,PLCL2,MET,MYL9,SRGAP3,ARPC1A,Bmp8b,RTN4,PDGFD,GNAL,NRP1,RAP1B,ADAM17,LRRC4C,PLXNA3,UNC5A,PDIA3,BDNF,ARHGEF7,SEMA6A,EPHA4,CRK,PLXNA2,EFNB2,WNT7A,MYSM1,EFNA5,DCC,TUBB4A,PSMD14,LNPEP,ROBO2,GNB1L,PPP3CA,UNC5C,MYL12A,BMP1,GNG4,ITGB1,TUBB1,PLXNA1,SEMA3G,EPHB4,ADAM2,NRP2,TUBB4B,MYLPF,ITGA2,PIK3C2G,EPHA3,SLIT2,MYL1,EFNA1,SEMA3A,SEMA4D,PAK3,MAG,NTRK3,ADAM10,EPHA5,SEMA4G,BMP7,SEMA3C,SEMA7A,NTN3,PLCB2,WNT3,EPHB2,FZD3,UNC5B,GNB2L1,SEMA6B,ADAM11,TUBB,LIMK1,ROCK2,SEMA6D,PLCE1,SEMA3D,MAPK3,UNC5D,ADAM23,WNT4,EFNB3,GSK3B,RTN4R,FZD2,ADAMTS4,TUBB3,GNG2,FZD9,GNAZ,HHIP,DPYSL5,GNG3,PDGFB,BMP10,HERC2,ADAM12,GNAO1,PLCB3,FZD10,ENPEP,ADAM22,ADAMTS7,SLIT1,ROBO1,ADAM21,SEMA4C,PRKAG1,EPHB6,NTNG1,PPP3CB,GLIS1,EFNB1,MKNK1,SMO,PLXNB1,AKT3,SEMA4A,SHANK2,MAP2K1,SEMA3F,ACE,SEMA3E,GNAS,PLXNC1,ARHGEF12,ARPC5L,ADAM15,C9orf3,GNAI1,TUBA4A,PRKAR2A,EFNA3,PLXND1,GIT1,ROCK1,GNAI2,EPHA10,NTRK2,FZD4,TUBB6,WNT10A,LINGO1,EPHB3,GLI1,SEMA4B,ADAM9,WNT11,WNT5A |
| Glutamate Receptor Signaling | 16.00 | 68.40% | GRM2,GRIN2A,SLC1A4,HOMER2,SLC17A6,GRM3,GRIA1,GRIN2D,GRID2,GRID1,SLC17A2,SLC1A3,GRIA4,GNB1,GRIK5,GRIN2C,HOMER1,DLG4,GRIK2,GRIK1,GRIN1,GRIN2B,SLC1A6,GRM1,GRM8,GLS,GRIK3,GNG2,GRIA2,GRIP1,SLC1A1,GRM4,GRM5,GRM7,GRIK4,SLC17A7,SLC1A2,GLUL,GRIA3 |
| Ephrin Receptor Signaling | 11.60 | 40.80% | RAF1,GRIN2A,EPHB2,ARPC5,GNB2L1,LIMK1,NCK2,VEGFA,ROCK2,GNB1,GNB4,EPHB1,ITGA3,PAK1,GRIN2C,MAPK3,ATF4,EFNB3,ITGA4,EFNA2,ACTR2,GNG2,CREBBP,ITGA5,GNG3,STAT3,GNAZ,CREB5,PDGFB,ATF2,ARPC1A,GNAO1,PDGFD,GNAL,RAP1B,PTPN13,GRIN2D,EPHA4,CRK,JAK2,EP300,EPHB6,EFNB2,EFNA5,EFNB1,AKT3,GNB1L,MAP2K1,ITGB1,GNG4,GRIN2B,GRIN1,EPHB4,GNAS,ANGPT1,ARPC5L,ITGA2,GNAI1,PIK3C2G,EFNA3,EPHA3,EFNA1,GNAI2,ROCK1,EPHA10,ABI1,PAK3,ADAM10,EPHA5,EPHB3,MAP4K4 |
| CREB Signaling in Neurons | 11.60 | 40.90% | RAF1,PLCB2,GRIN2A,POLR2D,GRM3,ADCY4,GNB2L1,GRIA4,GNB1,GNB4,GRIK5,PLCE1,CAMK2A,CAMK2D,GRIN2C,MAPK3,ATF4,PLCB1,GRIK1,POLR2I,ITPR2,GRM1,GRM8,CREBBP,GNG2,GRIA2,GNG3,ITPR1,GRM4,GNAZ,PLCL2,CREB5,ATF2,GRM7,ADCY9,ITPR3,GNAO1,PLCB3,GNAL,CAMK2G,GRM2,PDIA3,GRID1,GRID2,GRIN2D,GRIA1,POLR2B,PRKAG1,EP300,POLR2A,AKT3,GRIK2,GNB1L,MAP2K1,CAMK2B,GNG4,GRIN2B,GRIN1,GNAS,GRIK3,ADCY3,ADCY6,PRKAR2A,GNAI1,PIK3C2G,GRM5,GNAI2,GRIK4,ELK1,GRIA3 |
| Protein Kinase A Signaling | 10.40 | 31.90% | Dusp21,RAF1,MYH10,DUSP8,ADCY4,PTPN5,NTN1,GNB1,PTPRC,GNB4,PHKB,CAMK2A,GYS1,PTPRO,PLCB1,ATF4,MYL3,PDE2A,PTPRG,YWHAG,ADD2,PPP1R1B,CREBBP,PTCH1,YWHAZ,PLCL2,ITPR1,CREB5,ATF2,MYL9,ITPR3,PTPRA,CAMK2G,PDE6D,RAP1B,HIST1H1C,PTPN2,PDIA3,PTPN13,DUSP6,AKAP7,PTPN12,EP300,MPPE1,PTPN4,PTPRJ,DCC,RYR1,CTNNB1,GNB1L,PTPRT,PPP3CA,MYL12A,CAMK2B,MTMR3,GNG4,PTPRK,MYLPF,ADCY6,MYL1,ADD3,FLNC,ADD1,PTGS2,SFN,PTPN22,AKAP1,ENPP6,PLCB2,GNB2L1,PPP1R3A,YWHAQ,ROCK2,CAMK2D,PLCE1,RHO,PPP1R7,TGFB1,MAPK3,RYR3,GSK3B,CDC25A,YWHAE,ITPR2,YWHAB,GNG2,GNG3,TTN,PTPRM,ADCY9,PYGM,PTPRS,PLCB3,H1F0,SIRPA,PPP1CB,MYLK,PRKAG1,PTPRF,PPP3CB,PPP1R12A,SMO,TGFB2,SMAD4,PTPRZ1,CHUK,PTPRN,MAP2K1,CDC25C,GNAS,MAP3K1,RYR2,ADCY3,PRKAR2A,GNAI1,PYGB,GNAI2,ROCK1,Ptprd,PTPRU,AKAP4,ELK1,PTPRR |
| Epithelial Adherens Junction Signaling | 10.10 | 41.10% | TUBA1B,MYH10,MYH9,TGFBR3,ARPC5,MLLT4,TUBB,MYH7B,CLIP1,CTNNA2,MYL3,ACTR2,TUBB3,DLL1,NOTCH3,MYH14,FGFR1,TUBB2A,PTPRM,APC,MYL9,MET,EPN2,CDH2,ARPC1A,MYH3,ACTN4,CLINT1,ACTG1,NOTCH1,PVRL2,RAP1B,PVRL3,BMPR2,CRK,ACVR2B,IQGAP1,YES1,NOTCH2,HGF,TGFB2,AKT3,PVRL1,TUBB4A,VCL,CTNNB1,ACVR1C,MYH1,EGFR,TUBB1,TUBB4B,ARPC5L,ACTB,TUBA4A,ACVR1,MYL1,MAGI1,TUBB6,ACVR2A,CTNND1 |
| Coagulation System | 10.10 | 68.60% | KNG1,F12,PROC,PLAUR,VWF,THBD,F3,F2,FGG,SERPIND1,PLG,F11,KLKB1,SERPINC1,F9,F8,F5,SERPINA1,FGB,FGA,TFPI,A2M,F13B,PLAT |
| RhoGDI Signaling | 8.92 | 37.60% | ARPC5,GNB2L1,PIKFYVE,ARHGEF1,LIMK1,ROCK2,GNB1,GNB4,PAK1,ITGA3,RHOG,DLC1,CDH13,MYL3,ITGA4,ACTR2,GNG2,CREBBP,ITGA5,GRIP1,GNAZ,GNG3,MYL9,CDH2,CDH12,ARPC1A,CDH5,CDH20,GNAO1,CDH8,ACTG1,ARHGEF10,ESR2,GNAL,PIP4K2C,GDI1,ARHGEF7,CDH11,EP300,ARHGEF19,CDH7,PPP1R12A,GNB1L,MYL12A,GNG4,ITGB1,ARHGAP6,CDH4,ARHGEF12,GNAS,ARPC5L,MYLPF,ACTB,ITGA2,CDH6,GNAI1,CDH15,MYL1,GNAI2,ROCK1,DGKZ,CDH9,PAK3,CDH10,CD44 |
| Heparan Sulfate Biosynthesis | 8.84 | 53.40% | NDST3,B3GALT6,CHST7,HS2ST1,EXT1,CHST15,PRDX6,CHST2,HS6ST1,EXT2,UST,CHST3,HS6ST2,CHST11,CHST10,HS3ST1,HS6ST3,GLCE,CHST12,Sult1d1,SULT2A1,CHST1,SULT1E1,B3GAT1,EXTL3,B3GAT3,EXTL2,NDST1,SULT1B1,HS3ST5,EXTL1 |
| Heparan Sulfate Biosynthesis (Late Stages) | 8.40 | 54.90% | NDST3,CHST7,HS2ST1,EXT1,CHST15,PRDX6,CHST2,HS6ST1,EXT2,UST,CHST3,HS6ST2,CHST11,HS3ST1,HS6ST3,CHST10,GLCE,CHST12,Sult1d1,SULT2A1,CHST1,SULT1E1,EXTL3,EXTL2,NDST1,HS3ST5,EXTL1,SULT1B1 |
| Synaptic Long Term Depression | 8.21 | 38.70% | RAF1,PLCB2,GRM3,PLA2R1,PLA2G7,GRIA4,PLCE1,LCAT,PPM1J,MAPK3,RYR3,PPM1L,PLCB1,PLA2G4F,Gucy2g,GUCY1B3,ITPR2,GRM1,GRM8,GUCY2D,GRIA2,GRM4,PLCL2,GNAZ,ITPR1,GRM7,PLA2G6,PPP2CB,PPP2R1A,PPP2R4,NPR1,ITPR3,GNAO1,PLCB3,NPR2,GNAL,GRM2,PPP2R2A,PDIA3,GRID1,GRID2,GRIA1,PRDX6,PLB1,IGF1R,RYR1,NOS2,MAP2K1,GNAS,RYR2,GNAI1,CRHR1,GRM5,GNAI2,GRIA3 |
| GABA Receptor Signaling | 8.18 | 49.30% | GABRA5,UBQLN1,GABRA4,ADCY4,GPHN,SLC6A13,GABRB2,NSF,GABRG3,GABRB3,GABRG1,GPR37,GABRA6,GABRB1,GABRA1,GABRD,GABRA2,GABRA3,ALDH5A1,AP2M1,GNAS,ADCY3,ADCY6,GABBR1,KCNH2,DNM1,ADCY9,GABBR2,SLC6A11,GABRG2,GAD1,SLC6A1,SLC6A12 |
| Wnt/β-catenin Signaling | 8.08 | 36.70% | CSNK2A1,WNT3,FZD3,TGFBR3,CSNK1A1,BCL9,SOX13,TGFB1,PPM1J,PPM1L,WNT4,GSK3B,FZD2,TP53,SFRP4,GJA1,CREBBP,CSNK1D,FZD9,APC,PPP2CB,PPP2R1A,CDH2,CDH12,CDH5,PPP2R4,GNAO1,SFRP1,FZD10,FRZB,PPP2R2A,LRP6,MARK2,BMPR2,ACVR2B,KREMEN1,EP300,APPL1,WNT7A,DKK3,TGFB2,SMO,AKT3,CTNNB1,ACVR1C,LRP5,HDAC1,DVL1,ACVR1,DKKL1,FZD4,WNT10A,SOX6,TLE4,CD44,NR5A2,BTRC,PIN1,LRP1,WNT11,ACVR2A,WNT5A |
| Calcium Signaling | 7.51 | 35.40% | MYH10,GRIN2A,MYH9,CAMK1,CAMK1D,ATP2B1,MYH7B,GRIA4,ATP2A2,TRPC3,SLC8B1,CAMK2D,CAMK2A,GRIN2C,MAPK3,RYR3,ATF4,CASQ2,MYL3,GRIK1,HDAC4,HDAC2,ITPR2,MYH14,CREBBP,GRIA2,TPM3,ITPR1,TRPC6,CREB5,ATF2,HDAC5,MYL9,ITPR3,MYH3,CAMK2G,RAP1B,GRIN2D,GRIA1,PRKAG1,EP300,PPP3CB,RYR1,ASPH,PPP3CA,MYH1,CAMK2B,Trpc2,CALR,GRIN2B,GRIN1,TP63,CHRNA4,RYR2,TRDN,HDAC1,PRKAR2A,MYL1,TRPC7,ATP2B2,CHRNB2,CAMKK1,GRIA3 |
| Synaptic Long Term Potentiation | 7.42 | 39.50% | RAP1B,RAF1,GRM2,PLCB2,GRIN2A,PDIA3,GRM3,GRIA1,GRIN2D,PPP1CB,PPP1R3A,GRIA4,PRKAG1,EP300,CAMK2A,CAMK2D,PLCE1,PPP1R12A,PPP3CB,GRIN2C,PPP1R7,MAPK3,ATF4,PLCB1,MAP2K1,PPP3CA,CAMK2B,GRIN2B,GRIN1,ITPR2,GRM1,GRM8,CREBBP,PRKAR2A,GRIA2,CACNA1C,GRM4,PLCL2,ITPR1,CREB5,ATF2,GRM5,GRM7,ITPR3,PLCB3,GRIA3,CAMK2G |
| G-Protein Coupled Receptor Signaling | 7.28 | 32.00% | ENPP6,RAF1,PLCB2,HTR2B,GRM3,ADCY4,IKBKB,HRH1,CAMK2A,CAMK2D,MAPK3,PLCB1,ATF4,ADORA2B,ADRA1B,PDE2A,HRH2,GRM1,GRM8,CNR1,CREBBP,GRM4,STAT3,DRD2,CREB5,ATF2,FPR1,CHRM5,S1PR3,GRM7,ADCY9,ADRA2A,NPR3,GNAO1,PLCB3,PTGER2,GNAL,CAMK2G,PDE6D,GRM2,RGS18,HTR4,MC3R,DUSP6,CHRM4,AVPR1A,HRH3,PRKAG1,EP300,MPPE1,OPRL1,SYNGAP1,DRD1,AKT3,CHUK,ADORA1,MAP2K1,CAMK2B,ADRB2,GNAS,Agtr1b,NPY1R,ADCY3,GNAI1,PRKAR2A,ADCY6,PIK3C2G,FPR2,DRD5,GABBR1,CRHR1,GRM5,GNAI2,GABBR2,P2RY13,LPAR1,CCR4,GLP1R,CALCR,S1PR1,HTR1F,ADRA1A |
| Role of Macrophages, Fibroblasts and Endothelial Cells in Rheumatoid Arthritis | 7.06 | 30.70% | MAP2K4,RAF1,PLCB2,WNT3,FZD3,CSNK1A1,TLR8,FCGR1A,ROCK2,VEGFA,IKBKB,PLCE1,CAMK2D,CAMK2A,IL1RL2,TGFB1,MAPK3,WNT4,PLCB1,ATF4,LTBR,TRAF5,GSK3B,FZD2,IL1RAP,ADAMTS4,SFRP4,CREBBP,IL6R,FZD9,STAT3,PLCL2,TLR9,CREB5,APC,PDGFB,ATF2,C5,TLR2,GNAO1,PLCB3,SFRP1,IL1RAPL1,Ighg2b,PDGFD,CAMK2G,IL6ST,FZD10,FN1,ICAM1,FRZB,PDIA3,IL1RL1,LRP6,IGHG1,JAK2,IL17RA,EP300,ROR2,WNT7A,PPP3CB,DKK3,TLR7,SMO,AKT3,CHUK,TLR3,TNFRSF1B,NOS2,CTNNB1,MAP2K1,PPP3CA,CAMK2B,VCAM1,TNFSF11,LRP5,IL1RAPL2,DVL1,DAAM1,PIK3C2G,DKKL1,ROCK1,FZD4,WNT10A,CSF1,Tlr13,IRAK4,WNT11,LRP1,WNT5A,RYK |
| Ephrin B Signaling | 7.03 | 45.20% | EPHB2,GNB2L1,HNRNPK,LIMK1,GNB1,EPHB6,ROCK2,NCK2,EFNB2,GNB4,EPHB1,PAK1,MAPK3,EFNB1,ITSN2,EFNB3,CTNNB1,GNB1L,GNG4,EPHB4,GNAS,GNG2,GNAI1,GNAZ,GNG3,GNAI2,ROCK1,ABI1,GNAO1,CAP1,EPHB3,VAV1,GNAL |
| Dermatan Sulfate Biosynthesis | 6.81 | 48.30% | NDST3,B3GALT6,CHST7,CHPF,HS2ST1,CHST15,DSE,CHST2,HS6ST1,UST,CHST3,HS6ST2,CHST11,CHST10,HS3ST1,HS6ST3,CHSY3,CSGALNACT1,CHST12,Sult1d1,SULT2A1,CHST1,SULT1E1,B3GAT1,B3GAT3,NDST1,HS3ST5,SULT1B1 |
| Chondroitin Sulfate Biosynthesis | 6.78 | 49.10% | NDST3,B3GALT6,CHST7,CHPF,HS2ST1,CHST15,CHST2,HS6ST1,UST,CHST3,CHST11,HS6ST2,HS3ST1,HS6ST3,CHST10,CHSY3,CSGALNACT1,CHST12,Sult1d1,SULT2A1,CHST1,SULT1E1,B3GAT1,B3GAT3,NDST1,HS3ST5,SULT1B1 |
| Intrinsic Prothrombin Activation Pathway | 6.71 | 62.10% | KNG1,F12,KLK3,PROC,THBD,F2,FGG,F11,KLKB1,F9,SERPINC1,F8,F5,FGB,FGA,COL18A1,F13B,COL3A1 |
| Cellular Effects of Sildenafil (Viagra) | 6.63 | 37.20% | MYH10,PLCB2,MYH9,CACNG4,PDIA3,ADCY4,PPP1CB,MYLK,MYH7B,PRKAG1,CACNG2,CACNA1E,PLCE1,PPP1R12A,GPR37,CACNG7,PLCB1,CACNG8,GUCY1B3,MYL3,MYL12A,MYH1,PDE2A,GNAS,CACNA1D,ITPR2,GUCY2D,MYLPF,MYH14,ACTB,ADCY3,PRKAR2A,ADCY6,CACNA1C,PLCL2,ITPR1,KCNH2,MYL1,CACNA1A,CACNG3,MYL9,ADCY9,CACNG5,ITPR3,MYH3,PLCB3,ACTG1,SLC4A10 |
| cAMP-mediated signaling | 6.62 | 32.40% | ENPP6,RAF1,CAMK1,CAMK1D,GRM3,ADCY4,CAMK2A,CAMK2D,MAPK3,ATF4,ADORA2B,HRH2,PDE2A,GRM8,CNR1,CREBBP,GRM4,STAT3,CREB5,DRD2,CHRM5,FPR1,ATF2,S1PR3,GRM7,ADCY9,NPR3,ADRA2A,GNAO1,PTGER2,GNAL,PDE6D,CAMK2G,GRM2,RGS18,HTR4,MC3R,DUSP6,CHRM4,HRH3,AKAP7,OPRL1,MPPE1,EP300,DRD1,PPP3CB,ADORA1,MAP2K1,PPP3CA,CAMK2B,ADRB2,GNAS,NPY1R,ADCY3,ADCY6,PRKAR2A,GNAI1,FPR2,DRD5,GABBR1,CRHR1,GNAI2,GABBR2,P2RY13,AKAP4,LPAR1,CCR4,GLP1R,S1PR1,HTR1F,AKAP1 |
| Neuropathic Pain Signaling In Dorsal Horn Neurons | 6.59 | 40.00% | GRIN2A,GRM2,PLCB2,CAMK1,CAMK1D,PDIA3,BDNF,GRM3,GRIA1,GRIN2D,GRIA4,PRKAG1,CAMK2A,CAMK2D,PLCE1,GPR37,GRIN2C,MAPK3,PLCB1,CAMK2B,GRIN1,GRIN2B,ITPR2,GRM8,GRM1,PRKAR2A,PIK3C2G,GRIA2,GRM4,PLCL2,ITPR1,KCNH2,GRM5,GRM7,NTRK2,ITPR3,PLCB3,ELK1,GRIA3,CAMK2G |
| Chondroitin Sulfate Biosynthesis (Late Stages) | 6.50 | 51.10% | NDST3,CHSY3,CHST7,CHPF,HS2ST1,CHST12,CSGALNACT1,Sult1d1,CHST15,SULT2A1,CHST2,CHST1,HS6ST1,SULT1E1,UST,CHST3,CHST11,HS6ST2,HS3ST1,HS6ST3,CHST10,NDST1,SULT1B1,HS3ST5 |
| Signaling by Rho Family GTPases | 6.40 | 31.60% | MAP2K4,RAF1,MAP3K11,ARPC5,GNB2L1,PIKFYVE,ARHGEF1,SLC9A1,CLIP1,LIMK1,GNB1,STMN1,ROCK2,GNB4,ITGA3,PAK1,RHOG,MAPK3,CDH13,MYL3,ITGA4,ACTR2,SEPT7,GNG2,ITGA5,GNG3,GNAZ,MYL9,CDH2,CDH12,ARPC1A,CDH5,CYFIP1,CDH20,GNAO1,CDH8,MAPK10,ACTG1,ARHGEF10,GNAL,PIP4K2C,ARHGEF7,MYLK,SEPT11,IQGAP1,CDH11,ARHGEF19,CDH7,PPP1R12A,GNB1L,MAP2K1,MYL12A,ITGB1,GNG4,SEPT5,CDH4,ARHGEF12,GNAS,ARPC5L,MYLPF,ACTB,ITGA2,CDH6,GNAI1,PIK3C2G,VIM,CDH15,MYL1,GNAI2,ROCK1,CDH9,PAK3,CDH10,ELK1 |
| Amyotrophic Lateral Sclerosis Signaling | 6.38 | 39.80% | GRIN2A,Naip1 (includes others),CAPN11,GRIA1,GRIN2D,GRID2,GRID1,GRIA4,VEGFA,PAK1,GRIK5,CACNA1E,HECW1,GRIN2C,CASP1,AKT3,GRIK2,PPP3CA,GRIK1,TP53,GRIN1,GRIN2B,CACNA1D,GRIK3,PIK3C2G,GRIA2,CACNA1C,SOD1,CACNA1A,CCS,BCL2L1,CAPNS1,GRIK4,CAPN1,CAT,SLC1A2,GLUL,CAPN3,GRIA3 |
| Breast Cancer Regulation by Stathmin1 | 6.25 | 33.00% | TUBA1B,RAF1,PLCB2,CAMK1,CAMK1D,ADCY4,GNB2L1,ARHGEF1,PPP1R3A,TUBB,LIMK1,GNB1,ROCK2,STMN1,GNB4,PAK1,CAMK2D,CAMK2A,PPP1R7,MAPK3,PPM1J,PPM1L,PLCB1,TP53,TUBB3,ITPR2,TUBB2A,GNG2,GNG3,ITPR1,PPP2CB,ADCY9,PPP2R1A,PPP2R4,ITPR3,PLCB3,ARHGEF10,CAMK2G,PPP2R2A,ARHGEF7,PPP1CB,PRKAG1,ARHGEF19,PPP1R12A,TUBB4A,GNB1L,MAP2K1,CAMK2B,GNG4,TUBB1,ARHGEF12,GNAS,TUBB4B,ADCY3,PIK3C2G,ADCY6,PRKAR2A,TUBA4A,GNAI1,GNAI2,ROCK1,TUBB6,CDKN1B |
| Remodeling of Epithelial Adherens Junctions | 6.17 | 44.10% | TUBA1B,RALA,ARPC5,TUBB,IQGAP1,CLIP1,CTNNA2,HGF,TUBB4A,VCL,CTNNB1,ACTR2,TUBB1,TUBB3,ARPC5L,TUBB4B,ACTB,TUBB2A,TUBA4A,DNM3,APC,MET,DNM1,ARPC1A,TUBB6,ACTN4,DNM1L,ACTG1,MAPRE3,CTNND1 |
| Extrinsic Prothrombin Activation Pathway | 5.96 | 75.00% | F12,SERPINC1,F5,PROC,FGB,TFPI,THBD,FGA,F3,F13B,FGG,F2 |
| Gαi Signaling | 5.93 | 36.70% | RAF1,GRM2,RALA,GRM3,ADCY4,CHRM4,GNB2L1,HRH3,PRKAG1,OPRL1,GNB1,GNB4,MAPK3,GNB1L,ADORA1,GNG4,GNAS,NPY1R,GRM8,CNR1,ADCY3,GNG2,ADCY6,PRKAR2A,FPR2,GNAI1,GABBR1,STAT3,GRM4,GNG3,DRD2,FPR1,GRM7,GNAI2,S1PR3,GABBR2,P2RY13,ADCY9,LPAR1,NPR3,ADRA2A,CCR4,S1PR1,HTR1F |
| Integrin Signaling | 5.92 | 31.90% | MAP2K4,RAF1,MAP3K11,ARPC5,PIKFYVE,TLN1,ITGB3,NCK2,TSPAN3,PAK1,ITGA3,RHOG,ITGA9,ITGA11,MAPK3,ITGAV,ITGB4,GSK3B,TSPAN4,ITGB5,ITGA4,ACTR2,ITGA6,TSPAN2,ITGA5,TTN,PDGFB,MYL9,TLN2,ITGAM,ARPC1A,ARF3,CAPN1,ITGA1,ACTN4,TSPAN6,ACTG1,ITGA7,CAPN3,RAP1B,ITGA2B,RALA,CAPN11,TSPAN7,ARHGEF7,PPP1CB,CRK,MYLK,PPP1R12A,AKT3,VCL,MAP2K1,MYL12A,ITGB1,ARPC5L,ACTB,ITGA2,PIK3C2G,ITGAL,GIT1,ROCK1,ITGB2,CAPNS1,PAK3,NEDD9,ITGAX |
| Huntington’s Disease Signaling | 5.81 | 31.00% | MAP2K4,PLCB2,POLR2D,REST,GNB2L1,NAPG,GNB1,GNB4,NSF,CTSD,MAPK3,ATF4,DLG4,PLCB1,POLR2I,TP53,HDAC4,HDAC2,GRM1,CLTC,HSPA9,GNG2,CREBBP,DNM3,GNG3,ITPR1,CREB5,STX1A,RPH3A,GPAA1,ATF2,HDAC5,HSPA8,BCL2L1,DNAJC5,CACNA1B,CAPN1,PENK,PLCB3,CAPN3,SDHB,CAPN11,BDNF,PACSIN1,POLR2B,HSPA1L,EP300,MTOR,POLR2A,SP1,CASP1,IGF1R,AKT3,GOSR1,GNB1L,NAPB,EGFR,GNG4,GRIN2B,GLS,HDAC1,PIK3C2G,HSPA2,SNAP25,SIN3A,GRM5,DNM1,PSME1,CAPNS1,DNM1L,GOSR2 |
| CDK5 Signaling | 5.77 | 38.40% | RAF1,PPP2R2A,BDNF,ADCY4,PPP1CB,PPP1R3A,PRKAG1,LAMC1,ITGA3,DRD1,PPP1R12A,PPP1R7,MAPK3,PPM1J,PPM1L,LAMA1,LAMB1,MAP2K1,ITGB1,LAMA5,GNAS,PPP1R1B,ADCY3,ITGA2,PRKAR2A,ITGA6,ADCY6,DRD5,CACNA1A,PPP2CB,ADCY9,PPP2R1A,NTRK2,PPP2R4,MAPT,CAPN1,MAPK10,GNAL |
| Dopamine-DARPP32 Feedback in cAMP Signaling | 5.70 | 33.50% | PLCB2,GRIN2A,ADCY4,CSNK1A1,PPP1R3A,ATP2A2,PLCE1,CACNA1E,GRIN2C,PPP1R7,PPM1J,PPM1L,ATF4,PLCB1,GUCY1B3,KCNJ8,ITPR2,PPP1R1B,CREBBP,CSNK1D,CACNA1C,ITPR1,PLCL2,CREB5,DRD2,CACNA1A,ATF2,PPP2CB,ADCY9,PPP2R1A,PPP2R4,ITPR3,PLCB3,PPP2R2A,PDIA3,GRIN2D,PPP1CB,PRKAG1,EP300,PPP1R12A,DRD1,PPP3CB,PPP3CA,GRIN2B,GRIN1,GNAS,CACNA1D,ADCY3,ADCY6,PRKAR2A,GNAI1,DRD5,GNAI2,CAMKK1 |
| Caveolar-mediated Endocytosis Signaling | 5.69 | 42.30% | ITGA2B,HLA-A,COPA,ITGB3,ITGA3,ITGA9,ITGA11,ITGAV,ITGB4,ITGB5,EGFR,ITGA4,ITGB1,ACTB,ITGA2,CD48,ITGA6,ITGA5,COPG2,COPB1,ITGAL,ITGB2,ALB,ITGAM,FLNC,ITGA1,INSR,ITGA7,ACTG1,ITGAX |
| Dermatan Sulfate Biosynthesis (Late Stages) | 5.60 | 48.90% | NDST3,CHST7,HS2ST1,CHST12,Sult1d1,CHST15,SULT2A1,DSE,CHST2,HS6ST1,CHST1,SULT1E1,UST,CHST3,CHST11,HS6ST2,HS3ST1,HS6ST3,CHST10,NDST1,SULT1B1,HS3ST5 |
| Gap Junction Signaling | 5.52 | 33.50% | TUBA1B,RAF1,DBN1,PLCB2,HTR2B,ADCY4,CSNK1A1,TUBB,PLCE1,MAPK3,PLCB1,Gucy2g,GUCY1B3,TUBB3,GUCY2D,ITPR2,TUBB2A,CSNK1D,ITPR1,PLCL2,DRD2,ADCY9,NPR1,ITPR3,PLCB3,ACTG1,NPR2,PDIA3,PRKAG1,DRD1,PPP3CB,SP1,AKT3,TUBB4A,CTNNB1,MAP2K1,PPP3CA,EGFR,TUBB1,GNAS,TUBB4B,ACTB,ADCY3,TUBA4A,GNAI1,PRKAR2A,ADCY6,PIK3C2G,GNAI2,NOV,TUBB6,LPAR1 |
| phagosome maturation | 5.51 | 35.80% | TUBA1B,VPS39,PRDX1,HLA-A,ATP6V1C1,PIKFYVE,NAPG,ATP6V0D2,TUBB,ATP6AP1,PRDX6,NSF,Dync1i2,LAMP1,TUBB4A,GOSR1,ATP6V1G2,NAPB,M6PR,TUBB1,ATP6V0E2,CALR,TUBB3,DCTN4,TUBB4B,VPS41,TUBB2A,TUBA4A,ATP6V1A,GPAA1,SNAP25,ATP6V0A1,LAMP2,MPO,TUBB6,DYNC1LI2,ATP6V0A2,VPS37B,GOSR2,ATP6V0E1,ATP6V1B2,HLA-DRB5,EEA1 |
| Gαs Signaling | 5.46 | 36.70% | HTR4,MC3R,ADCY4,GNB2L1,PRKAG1,EP300,GNB1,GNB4,DRD1,MAPK3,RYR3,ATF4,RYR1,ADORA2B,GNB1L,MAP2K1,ADRB2,GNG4,HRH2,GNAS,ADD2,CNR1,RYR2,GNG2,CREBBP,ADCY3,ADCY6,PRKAR2A,DRD5,GNG3,CRHR1,CREB5,CHRM5,ATF2,ADCY9,ADD3,GLP1R,ADD1,PTGER2,ELK1 |
| Acute Phase Response Signaling | 5.34 | 32.50% | MAP2K4,RAF1,ITIH3,SERPINA3,CP,FGG,F2,IKBKB,SOD2,ITIH4,APCS,MAPK3,FGB,OSMR,IL1RAP,TTR,C3,C1S,IL6R,STAT3,HMOX2,C5,KLKB1,FGA,IL6ST,SERPING1,FN1,APOH,AMBP,C9,JAK2,HNRNPK,SERPIND1,C1R,C4A/C4B,MTOR,F8,CFB,AKT3,SERPINA1,CHUK,TNFRSF1B,MAP2K1,AGT,HPX,AHSG,MAP3K1,VWF,SOCS4,PLG,ALB,HP,TF,ELK1,A2M |
| Complement System | 5.32 | 51.40% | CFD,SERPING1,C3,C1S,C9,C1QA,C5,C4A/C4B,C1R,ITGB2,ITGAM,CFI,CFB,C8B,CFH,C1QBP,C3AR1,C8G,ITGAX |
| Sertoli Cell-Sertoli Cell Junction Signaling | 5.27 | 32.00% | MAP2K4,TUBA1B,MAP3K15,RAF1,MAP3K11,TGFBR3,JAM2,MLLT4,TUBB,MPP6,CTNNA2,ITGA3,MAPK3,Map3k7,GSK3B,GUCY1B3,ITGA4,DLG1,TUBB3,TUBB2A,ITGA5,ATF2,EPN2,CLDN12,JAM3,MAPK10,ACTN4,SPTAN1,CLINT1,ACTG1,PVRL2,SPTBN1,PVRL3,SPTA1,PRKAG1,AKT3,PVRL1,TUBB4A,VCL,CTNNB1,NOS2,MAP2K1,RAB8B,ITGB1,TUBB1,TJP1,TUBB4B,ACTB,MAP3K1,ITGA2,PRKAR2A,TUBA4A,F11R,TUBB6,ELK1,A2M,CLDN22 |
| Human Embryonic Stem Cell Pluripotency | 5.26 | 34.30% | FZD10,KLK3,WNT3,BDNF,FZD3,BMPR2,NOG,FGFR3,WNT7A,TGFB1,SMO,TGFB2,PDGFRA,AKT3,WNT4,SMAD4,GSK3B,CTNNB1,FZD2,BMP1,PDGFRB,S1PR2,GNAS,S1PR5,FGFR1,DVL1,ACVR1,PIK3C2G,FGFR2,FZD9,BMP10,PDGFB,APC,S1PR3,NTRK2,FZD4,FOXO1,WNT10A,Bmp8b,NTRK3,S1PR1,BMP7,FGFRL1,PDGFD,WNT11,WNT5A |
| HIPPO signaling | 5.09 | 38.40% | SAV1,PPP2R2A,FAT4,DLG2,PPP1CB,PPP1R3A,YWHAQ,TEAD1,PPP1R12A,PPP1R7,PPM1J,PPM1L,DLG4,SMAD4,AMOT,LATS1,DLG1,YWHAG,YWHAE,YWHAB,CSNK1D,YWHAZ,STK3,DLG3,ITCH,PPP2CB,PPP2R1A,PPP2R4,CD44,BTRC,TEAD3,INADL,SFN |
| Gα12/13 Signaling | 5.01 | 35.00% | MAP2K4,RAF1,LPAR4,F2RL2,ARHGEF1,CDH11,F2,ROCK2,IKBKB,CDH7,MAPK3,AKT3,CHUK,CTNNB1,CDH13,MAP2K1,MYL3,MYL12A,CDH4,MYLPF,MAP3K1,PIK3C2G,CDH6,CDH15,LPAR3,MYL1,ROCK1,BTK,MYL9,CDH2,CDH9,CDH12,LPAR1,CDH5,CDH20,CDH10,MAPK10,LPAR5,CDH8,VAV1,ELK1 |
| LXR/RXR Activation | 5.00 | 34.70% | KNG1,APOB,APOH,IL1RL1,VTN,CD36,AMBP,C9,ABCA1,C4A/C4B,PON1,LCAT,IL1RL2,ITIH4,FASN,SERPINA1,TLR3,NOS2,GC,TNFRSF1B,IL1RAP,PON3,AGT,HPX,TTR,C3,IL1RAPL2,AHSG,PCYOX1,A1BG,ALB,LDLR,TF,CD14,ACACA,IL1RAPL1,PLTP,PTGS2,FGA,HMGCR,CLU,APOD |
| Colorectal Cancer Metastasis Signaling | 4.99 | 29.70% | MAP2K4,WNT3,MSH3,FZD3,ADCY4,MMP16,GNB2L1,TLR8,VEGFA,GNB1,GNB4,RHOG,TGFB1,MAPK3,WNT4,GSK3B,FZD2,TP53,GNG2,IL6R,TYK2,FZD9,IFNGR1,GNG3,STAT3,TLR9,APC,TLR2,BCL2L1,ADCY9,MAPK10,PTGER2,IL6ST,FZD10,MMP15,LRP6,JAK2,PRKAG1,APPL1,WNT7A,TGFB2,SMO,TLR7,DCC,AKT3,SMAD4,TLR3,GNB1L,MMP12,NOS2,CTNNB1,MAP2K1,MMP19,EGFR,GNG4,LRP5,GNAS,DVL1,ADCY3,ADCY6,PRKAR2A,PIK3C2G,FZD4,WNT10A,MSH6,Tlr13,PTGS2,WNT11,LRP1,WNT5A |
| Agrin Interactions at Neuromuscular Junction | 4.95 | 40.60% | MAP2K4,NRG2,ARHGEF7,ITGB3,LAMC1,ITGA3,PAK1,MAPK3,ERBB4,LAMB1,DAG1,EGFR,ITGA4,ITGB1,ACTB,DVL1,ITGA2,LAMA2,ITGA6,ITGA5,ERBB3,ITGAL,ITGB2,PAK3,MAPK10,ITGA1,AGRN,ACTG1 |
| STAT3 Pathway | 4.89 | 39.70% | MAP2K4,RAF1,MAP3K11,PTPN2,TGFBR3,BMPR2,JAK2,FGFR3,MAPK3,IGF1R,PDGFRA,MAP2K1,EGFR,CDC25A,PDGFRB,FLT1,FGFR1,TYK2,FGFR2,SOCS4,STAT3,TNFRSF11A,IGF2R,NTRK2,NTRK3,MAPK10,INSR,FGFRL1,KDR |
| Circadian Rhythm Signaling | 4.84 | 51.50% | GRIN2B,AVP,GRIN1,GRIN2A,GRIN2D,CREBBP,CSNK1D,VIP,CREB5,ATF2,EP300,ADCYAP1R1,NR1D1,GRIN2C,ATF4,CRY1,PER2 |
| Corticotropin Releasing Hormone Signaling | 4.83 | 35.10% | RAP1B,RAF1,CRHR2,BDNF,ADCY4,ARPC5,PRKAG1,EP300,VEGFA,MAPK3,SMO,ATF4,NOS2,MAP2K1,Gucy2g,GUCY1B3,GNAS,GUCY2D,ITPR2,CNR1,CREBBP,ADCY3,PTCH1,ADCY6,PRKAR2A,GNAI1,ITPR1,CRHR1,CREB5,ATF2,GNAI2,ADCY9,NPR1,GNAO1,ITPR3,PTGS2,ELK1,GLI1,NPR2 |
| PTEN Signaling | 4.52 | 33.90% | RAF1,CSNK2A1,TGFBR3,BMPR2,FGFR3,IKBKB,ITGA3,MAPK3,FOXO3,PDGFRA,IGF1R,AKT3,GSK3B,CHUK,MAP2K1,PDGFRB,ITGA4,EGFR,ITGB1,FLT1,FGFR1,ITGA2,PREX2,FGFR2,ITGA5,TNFRSF11A,IGF2R,INPP5D,BCL2L1,NTRK2,MAGI1,FOXO6,SYNJ1,FOXO1,NTRK3,FGFRL1,INSR,CDKN1B,KDR,MAGI3 |
| Cardiac Hypertrophy Signaling | 4.43 | 29.10% | MAP2K4,MAP3K15,RAF1,PLCB2,MAP3K11,ADCY4,GNB2L1,ROCK2,GNB1,GNB4,RHOG,PLCE1,CACNA1E,TGFB1,MAPK3,PLCB1,Map3k7,GSK3B,ADRA1B,MYL3,GNG2,IL6R,CREBBP,CACNA1C,GNG3,GNAZ,PLCL2,CACNA1A,ATF2,MYL9,ADCY9,ADRA2A,GNAO1,MAPK10,PLCB3,GNAL,PDIA3,ATF6,PRKAG1,EP300,MTOR,PPP3CB,TGFB2,IGF1R,GNB1L,MAP2K1,PPP3CA,ADRB2,MYL12A,GNG4,CACNA1D,GNAS,MYLPF,MAP3K1,ADCY3,ADCY6,PRKAR2A,GNAI1,PIK3C2G,MYL1,GNAI2,ROCK1,ELK1,ADRA1A,GATA4 |
| PI3K/AKT Signaling | 4.42 | 33.30% | RAF1,PPP2R2A,JAK2,YWHAQ,IKBKB,HSP90B1,MTOR,ITGA3,GYS1,HSP90AB1,PPM1J,MAPK3,FOXO3,TSC2,PPM1L,AKT3,GSK3B,CHUK,CTNNB1,MAP2K1,ITGA4,TP53,ITGB1,YWHAG,YWHAE,YWHAB,ITGA2,TYK2,YWHAZ,ITGA5,INPP5D,BCL2L1,PPP2CB,PPP2R1A,SYNJ1,FOXO1,PPP2R4,HSP90AA1,PTGS2,CDKN1B,SFN |
| Thrombin Signaling | 4.34 | 30.00% | RAF1,PLCB2,CAMK1,CAMK1D,ADCY4,GNB2L1,ARHGEF1,F2,ROCK2,GNB1,GNB4,IKBKB,RHOG,PLCE1,CAMK2D,CAMK2A,MAPK3,PLCB1,MYL3,ITPR2,GNG2,ITPR1,PLCL2,GNAZ,GNG3,MYL9,ADCY9,ITPR3,GNAO1,PLCB3,ARHGEF10,GNAL,CAMK2G,F2RL2,PDIA3,PPP1CB,MYLK,PPP1R12A,AKT3,GNB1L,MAP2K1,MYL12A,EGFR,CAMK2B,GNG4,ARHGEF12,GNAS,MYLPF,ADCY3,PIK3C2G,ADCY6,GNAI1,MYL1,GNAI2,ROCK1,ELK1,GATA4 |
| PPARα/RXRα Activation | 4.29 | 30.30% | MAP2K4,RAF1,PLCB2,CD36,ADCY4,TGFBR3,CPT1B,ABCA1,NR2F1,IKBKB,PLCE1,IL1RL2,TGFB1,MAPK3,FASN,PLCB1,IL1RAP,ITGB5,Cyp2c40 (includes others),MED1,CREBBP,PLCL2,ADCY9,ACADL,HSP90AA1,PLCB3,IL1RAPL1,INSR,PDIA3,IL1RL1,BMPR2,ACVR2B,JAK2,PRKAG1,EP300,HSP90B1,HSP90AB1,TGFB2,SMAD4,CHUK,GOT2,STAT5B,MAP2K1,ACVR1C,GNAS,IL1RAPL2,ADCY3,ADCY6,PRKAR2A,ACVR1,CKAP5,TGS1,MAP4K4,ACVR2A |
| Paxillin Signaling | 4.26 | 34.70% | MAP2K4,ITGA2B,ARHGEF7,TLN1,CRK,PTPN12,ITGB3,NCK2,ITGA3,PAK1,ITGA9,ITGA11,ITGAV,VCL,ITGB4,ITGB5,ITGA4,ITGB1,ACTB,ITGA2,PIK3C2G,ITGA6,ITGA5,ITGAL,GIT2,ITGB2,ITGAM,TLN2,PAK3,MAPK10,ITGA1,ACTN4,ITGA7,ACTG1,ITGAX |
| Role of Osteoblasts, Osteoclasts and Chondrocytes in Rheumatoid Arthritis | 4.13 | 28.80% | MAP2K4,Naip1 (includes others),WNT3,FZD3,CSNK1A1,ITGB3,IKBKB,ITGA3,IL1RL2,TGFB1,MAPK3,WNT4,TRAF5,GSK3B,IL1RAP,FZD2,ADAMTS4,SFRP4,ITGA5,FZD9,APC,BMP10,Bmp8b,MAPK10,SFRP1,IL1RAPL1,FZD10,ADAM17,FRZB,IL1RL1,LRP6,BMPR2,WNT7A,PPP3CB,DKK3,SMO,AKT3,SMAD4,CHUK,CTNNB1,TNFRSF1B,ALPL,PPP3CA,BMP1,ITGB1,LRP5,TNFSF11,IL1RAPL2,ITGA2,DVL1,PIK3C2G,DKKL1,TNFRSF11A,CSF1R,FZD4,WNT10A,FOXO1,CSF1,CALCR,BMP7,LRP1,WNT11,WNT5A |
| Factors Promoting Cardiogenesis in Vertebrates | 3.98 | 34.80% | FZD10,WNT3,FZD3,TGFBR3,LRP6,BMPR2,ACVR2B,NOG,TGFB1,SMO,TGFB2,SMAD4,GSK3B,CTNNB1,FZD2,ACVR1C,BMP1,LRP5,CER1,DVL1,ACVR1,FZD9,BMP10,APC,ATF2,FZD4,Bmp8b,BMP7,LRP1,WNT11,ACVR2A,GATA4 |
| Hepatic Fibrosis / Hepatic Stellate Cell Activation | 3.94 | 29.50% | MYH10,MYH9,MYH7B,VEGFA,IL1RL2,TGFB1,LAMA1,IL1RAP,MYL3,PDGFRB,FGFR1,MYH14,IL6R,FGFR2,IFNGR1,IFNAR2,PDGFB,MYL9,MET,MYH3,CD14,COL6A5,IL1RAPL1,PDGFD,COL3A1,FN1,ICAM1,IL1RL1,COL6A6,COL6A1,TIMP1,HGF,TGFB2,IGF1R,PDGFRA,SMAD4,ECE1,COL18A1,TNFRSF1B,AGT,EGFR,MYH1,VCAM1,FLT1,COL6A2,IL1RAPL2,EDNRB,Agtr1b,COL12A1,MYL1,CSF1,EDNRA,KDR,A2M |
| Role of NFAT in Cardiac Hypertrophy | 3.92 | 29.60% | MAP2K4,RAF1,PLCB2,CAMK1,CAMK1D,ADCY4,GNB2L1,CSNK1A1,GNB1,GNB4,PLCE1,CAMK2A,CAMK2D,TGFB1,MAPK3,PLCB1,GSK3B,HDAC4,HDAC2,ITPR2,GNG2,GNG3,ITPR1,PLCL2,HDAC5,ADCY9,ITPR3,MAPK10,PLCB3,CAMK2G,IL6ST,PDIA3,PRKAG1,EP300,PPP3CB,TGFB2,IGF1R,AKT3,GNB1L,MAP2K1,PPP3CA,CAMK2B,GNG4,GNAS,ADCY3,MAP3K1,HDAC1,PIK3C2G,ADCY6,PRKAR2A,GNAI1,GNAI2,GATA4 |
| GNRH Signaling | 3.90 | 31.80% | MAP2K4,RAF1,MAP3K15,PLCB2,MAP3K11,ADCY4,PRKAG1,EP300,PAK1,CAMK2D,CAMK2A,MAPK3,Map3k7,PLCB1,ATF4,MAP2K1,EGFR,CAMK2B,GNAS,ITPR2,CREBBP,ADCY3,MAP3K1,ADCY6,PRKAR2A,GNAI1,DNM3,ITPR1,CREB5,ATF2,GNAI2,DNM1,ADCY9,PAK3,ITPR3,MAPK10,PLCB3,DNM1L,ELK1,GNRHR,CAMK2G |
| Tight Junction Signaling | 3.87 | 29.90% | MYH10,F2RL2,MYH9,PPP2R2A,VAPA,PVRL3,MARK2,MPP5,NAPG,JAM2,MLLT4,MYLK,MYH7B,PRKAG1,NSF,TGFB1,PPM1J,PPM1L,TGFB2,AKT3,PVRL1,VCL,GOSR1,TNFRSF1B,CTNNB1,MYL3,NAPB,MYH1,TJP1,MYH14,ACTB,PRKAR2A,CASK,GPAA1,SNAP25,MYL1,MYL9,F11R,PPP2CB,PPP2R1A,CLDN12,PPP2R4,JAM3,MYH3,SPTAN1,INADL,GOSR2,ACTG1,CLDN22,PVRL2 |
| PCP pathway | 3.82 | 38.10% | CELSR1,MAP2K4,FZD10,WNT3,FZD3,DAAM1,DVL1,FZD9,ATF2,ROCK2,ROCK1,ROR2,WNT7A, FZD4,WNT10A,EFNB1,MAPK10,SMO,WNT4,PRICKLE1,FZD2,LGR4,WNT11,WNT5A |
| Wnt/Ca+ pathway | 3.77 | 39.30% | FZD10,PLCB2,FZD3,PDIA3,CREBBP,DVL1,FZD9,PLCL2,CREB5,ATF2,EP300,PLCE1,FZD4, CAMK2A,SMO,ATF4,PLCB3,PLCB1,GSK3B,FZD2,PPP3CA,WNT5A |
| Actin Cytoskeleton Signaling | 3.77 | 28.20% | KNG1,MYH10,RAF1,MYH9,ARPC5,PIKFYVE,ARHGEF1,TLN1,SSH1,MYH7B,SLC9A1,F2,LIMK1,ROCK2,ITGA3,PAK1,CYFIP2,MAPK3,SSH2,MYL3,ITGA4,ACTR2,MYH14,ITGA5,TTN,PDGFB,APC,MYL9,TLN2,ARPC1A,CYFIP1,MYH3,CD14,VAV1,ACTN4,PDGFD,ACTG1,PIP4K2C,FN1,ARHGEF7,PPP1CB,TRIO,CRK,MYLK,IQGAP1,PPP1R12A,VCL,MAP2K1,MYH1,MYL12A,ITGB1,ARHGEF12,ARPC5L,MYLPF,ACTB,ITGA2,PIK3C2G,GIT1,MYL1,ROCK1,PAK3 |
| Molecular Mechanisms of Cancer | 3.77 | 25.80% | MAP2K4,RAF1,Naip1 (includes others),PLCB2,WNT3,FZD3,ADCY4,NCSTN,ARHGEF1,RB1,CTNNA2,PAK1,ITGA3,RHOG,CAMK2D,CAMK2A,TGFB1,MAPK3,WNT4,PLCB1,GSK3B,BRCA1,FZD2,CDC25A,ITGA4,TP53,CREBBP,TYK2,PTCH1,ITGA5,FZD9,GNAZ,APC,BMP10,ADCY9,BCL2L1,Bmp8b,GNAO1,MAPK10,PLCB3,ARHGEF10,NOTCH1,GNAL,CAMK2G,RAP1B,FZD10,RALA,ARHGEF7,LRP6,BMPR2,CRK,JAK2,PRKAG1,EP300,SYNGAP1,ARHGEF19,WNT7A,SMO,TGFB2,SMAD4,AKT3,CTNNB1,MAP2K1,CAMK2B,BMP1,ITGB1,PRKDC,CDC25C,LRP5,ARHGEF12,GNAS,ITGA2,DVL1,ADCY3,GNAI1,PRKAR2A,ADCY6,PIK3C2G,SIN3A,GNAI2,FZD4,FOXO1,WNT10A,NF1,PAK3,ATR,BMP7,CDKN1B,ELK1,GLI1,WNT11,LRP1,WNT5A,CTNND1 |
| GPCR-Mediated Integration of Enteroendocrine Signaling Exemplified by an L Cell | 3.76 | 36.60% | PLCB2,PDIA3,NMB,ADCY4,PRKAG1,PLCE1,PLCB1,SST,ADRB2,CCKAR,GNAS,ITPR2,ADCY3,GNAI1,PRKAR2A,ADCY6,PLCL2,ITPR1,VIP,GNAI2,ADCY9,GLP1R,ITPR3,NPY2R,PLCB3,GALR1 |
| Cell Cycle: G2/M DNA Damage Checkpoint Regulation | 3.75 | 40.80% | TP53,PRKDC,CDC25C,YWHAG,YWHAE,YWHAB,WEE1,PTPMT1,YWHAZ,PLK1,CCNB1,EP300,YWHAQ,TOP2B,TRIP12,BTRC,BORA,ATR,SFN,BRCA1 |
| PI3K Signaling in B Lymphocytes | 3.72 | 31.50% | CD81,RAF1,PLCB2,PDIA3,ATF6,FCGR2B,PTPRC,IKBKB,PLCE1,CAMK2D,CAMK2A,PPP3CB,MAPK3,FOXO3,ATF4,AKT3,ATF6B,PLCB1,IRS2,CHUK,MAP2K1,PPP3CA,CAMK2B,CD19,C3,ITPR2,PLCL2,ITPR1,INPP5D,ATF2,BTK,CD180,DAPP1,BCL10,SH2B2,ITPR3,PLCB3,VAV1,ELK1,CAMK2G |
| Semaphorin Signaling in Neurons | 3.69 | 39.60% | DPYSL2,ITGB1,PLXNA1,ARHGEF12,DPYSL3,DPYSL4,DPYSL5,LIMK1,ROCK2,ROCK1,MET,SEMA3A,CRMP1,PAK1,RHOG,SEMA4D,PAK3,MAPK3,PLXNB1,SEMA7A,NRP1 |
| Neuroprotective Role of THOP1 in Alzheimer’s Disease | 3.52 | 42.50% | MME,KNG1,YWHAE,HLA-A,PRKAR2A,SERPINA3,IDE,PRKAG1,APP,PLG,ECE2,MAPT,SST, ECE1,HLA-E,ACE,AGT |
| PAK Signaling | 3.50 | 33.70% | MAP2K4,RAF1,ARHGEF7,MYLK,LIMK1,NCK2,ITGA3,PAK1,MAPK3,PDGFRA,MAP2K1,MYL3,MYL12A,PDGFRB,ITGA4,ITGB1,MYLPF,ITGA2,PIK3C2G,ITGA5,EPHA3,GIT1,PDGFB,MYL1,MYL9,PAK3,MAPK10,EPHB3,DSCAM,PDGFD |
| Gαq Signaling | 3.48 | 29.90% | RAF1,PLCB2,HTR2B,RGS18,GNB2L1,AVPR1A,ROCK2,GNB1,HRH1,IKBKB,GNB4,GYS1,RHOG,PPP3CB,MAPK3,GPLD1,AKT3,PLCB1,GSK3B,CHUK,GNB1L,MAP2K1,PPP3CA,ADRA1B,GNG4,PLD3,GNAS,GRM1,Agtr1b,ITPR2,GNG2,PIK3C2G,ITPR1,GNG3,CHRM5,ROCK1,PLD4,GRM5,BTK,ITPR3,CALCR,PLCB3,ELK1,ADRA1A |
| CXCR4 Signaling | 3.43 | 29.60% | MAP2K4,RAF1,PLCB2,CD4,ADCY4,GNB2L1,CRK,ROCK2,GNB1,ELMO3,GNB4,PAK1,RHOG,MAPK3,AKT3,PLCB1,GNB1L,MAP2K1,MYL3,MYL12A,GNG4,GNAS,ITPR2,MYLPF,GNG2,ADCY3,GNAI1,ADCY6,PIK3C2G,GNAZ,ITPR1,GNG3,MYL1,ROCK1,GNAI2,MYL9,ADCY9,PAK3,GNAO1,ITPR3,MAPK10,PLCB3,ELK1,ELMO1,GNAL |
| AMPK Signaling | 3.42 | 28.70% | CPT1B,AK1,GYS1,PPM1J,FASN,PPM1L,TSC2,ATF4,IRS2,SMARCC2,ADRA1B,SLC2A1,EEF2,CREBBP,CREB5,SLC2A4,ATF2,PPP2CB,PPP2R1A,ADRA2A,PPP2R4,ACACA,INSR,AK2,PBRM1,ARID1A,PPP2R2A,PRKAG1,EP300,MTOR,FOXO3,AKT3,EEF2K,GNB1L,ADRB2,CHRNA4,GNAS,CKM,ACTB,PRKAR2A,PIK3C2G,PFKP,PFKFB2,FOXO6,FOXO1,PPM1B,SMARCC1,ACTL6B,HMGCR,HLTF,ADRA1A |
| Role of NANOG in Mammalian Embryonic Stem Cell Pluripotency | 3.35 | 31.50% | IL6ST,RAF1,FZD10,T,WNT3,FZD3,BMPR2,JAK2,LIFR,WNT7A,MAPK3,SMO,AKT3,WNT4,SMAD4,GSK3B, CTNNB1,FZD2,MAP2K1,BMP1,TP53,TYK2,DVL1,PIK3C2G,FZD9,STAT3,APC,BMP10,FZD4,WNT10A,Bmp8b, BMP7,WNT11,GATA4,WNT5A |
| Phospholipase C Signaling | 3.34 | 27.00% | RAF1,PLCB2,ADCY4,GNB2L1,ARHGEF1,GNB1,GNB4,ITGA3,PLCE1,RHOG,MAPK3,ATF4,PLCB1,PLA2G4F,MYL3,ITGA4,PLD3,HDAC4,HDAC2,ITPR2,GNG2,CREBBP,ITGA5,GNG3,ITPR1,CREB5,ATF2,HDAC5,MYL9,PLA2G6,ADCY9,ITPR3,PLCB3,Ighg2b,ARHGEF10,RAP1B,PEBP1,RALA,ARHGEF7,PPP1CB,IGHG1,FCGR2B,EP300,ARHGEF19,PPP1R12A,PPP3CB,GPLD1,GNB1L,MAP2K1,PPP3CA,MYL12A,GNG4,ITGB1,ARHGEF12,GNAS,FCGR2A,MYLPF,ITGA2,HDAC1,ADCY3,ADCY6,MYL1,BTK,PLD4 |
| Reelin Signaling in Neurons | 3.32 | 34.20% | MAP2K4,MAP3K11,ARHGEF1,ITGB3,ITGA3,YES1,GSK3B,ITGA4,ITGB1,ARHGEF12,CNR1,ITGA2,ITGA6,PIK3C2G,ITGA5,MAPK8IP3,RELN,ITGAL,APP,ITGB2,MAPT,MAPK10,ITGA1,LRP8,PAFAH1B1,ARHGEF10,DCX |
| Glioblastoma Multiforme Signaling | 3.25 | 29.50% | RAF1,FZD10,PLCB2,WNT3,PDIA3,FZD3,RB1,MTOR,RHOG,WNT7A,PLCE1,MAPK3,TSC2,SMO,IGF1R,PDGFRA,AKT3,PLCB1,WNT4,GSK3B,CTNNB1,FZD2,MAP2K1,EGFR,PDGFRB,TP53,ITPR2,PIK3C2G,FZD9,PLCL2,ITPR1,PDGFB,APC,FZD4,FOXO1,WNT10A,NF1,ITPR3,PLCB3,CDKN1B,PDGFD,WNT11,WNT5A |
| Basal Cell Carcinoma Signaling | 3.23 | 34.70% | FZD10,WNT3,FZD3,WNT7A,GLIS1,SMO,WNT4,GSK3B,CTNNB1,FZD2,BMP1,TP53,DVL1,PTCH1,FZD9,HHIP,BMP10,APC,FZD4,WNT10A,Bmp8b,BMP7,GLI1,WNT11,WNT5A |
| Germ Cell-Sertoli Cell Junction Signaling | 3.18 | 28.80% | MAP2K4,MAP3K15,TUBA1B,MAP3K11,PVRL3,MLLT4,TUBB,IQGAP1,LIMK1,CTNNA2,ITGA3,PAK1,RHOG,TGFB1,MAPK3,TGFB2,Map3k7,TUBB4A,VCL,CTNNB1,MAP2K1,RAB8B,ITGB1,TUBB1,TUBB3,TJP1,TUBB4B,ACTB,MAP3K1,ITGA2,TUBB2A,ITGA6,TUBA4A,PIK3C2G,LAMC3,EPN2,CDH2,TUBB6,PAK3,MAPK10,ACTN4,CLINT1,A2M,ACTG1,PVRL2,CTNND1 |
| Dermatan Sulfate Degradation (Metazoa) | 3.15 | 56.20% | HYAL2,IDUA,IDS,GM2A,HYAL1,CD44,HEXB,HEXA,FGFRL1 |
| IL-8 Signaling | 3.06 | 27.70% | MAP2K4,RAF1,ANGPT2,PLCB2,ICAM1,GNB2L1,IQGAP1,ITGB3,LIMK1,GNB1,RAB11FIP2,ROCK2,VEGFA,IKBKB,GNB4,MTOR,RHOG,MAPK3,GPLD1,ITGAV,AKT3,CHUK,GNB1L,MAP2K1,ITGB5,TEK,EGFR,GNG4,VCAM1,GNAS,PLD3,ANGPT1,FLT1,GNG2,PIK3C2G,GNAI1,GNG3,GNAI2,PLD4,ROCK1,MYL9,ITGB2,BCL2L1,ITGAM,MPO,MAPK10,PTGS2,KDR,MAP4K4,IRAK4,ITGAX |
| p70S6K Signaling | 3.05 | 30.30% | RAF1,PLCB2,F2RL2,PPP2R2A,PDIA3,F2,YWHAQ,MTOR,PLCE1,PPM1J,MAPK3,PPM1L,AKT3,PLCB1,EEF2K,BCAP31,MAP2K1,AGT,EGFR,CD19,YWHAG,YWHAE,EEF2,YWHAB,GNAI1,YWHAZ,PIK3C2G,PLCL2,GNAI2,BTK,PPP2CB,PPP2R1A,PPP2R4,MAPT,PLCB3,SFN |
| Mitotic Roles of Polo-Like Kinase | 3.04 | 34.80% | FZR1,KIF23,SMC3,CDC25C,CDC20,PPP2R2A,WEE1,PRC1,PLK1,CCNB1,PPP2CB,HSP90B1,PPP2R1A,PLK4,HSP90AB1,PPP2R4,TGFB1,PPM1J,CAPN1,PPM1L,HSP90AA1,KIF11,CDC25A |
| ILK Signaling | 3.00 | 27.60% | MAP2K4,MYH10,MYH9,RICTOR,MYH7B,ITGB3,NCK2,VEGFA,RHOG,PPM1J,MAPK3,PPM1L,ATF4,IRS2,GSK3B,ITGB4,TESK1,ITGB5,MYL3,MYH14,CREBBP,CREB5,ATF2,MYL9,PPP2CB,PPP2R1A,PPP2R4,MAPK10,MYH3,ACTN4,ACTG1,FN1,PPP2R2A,EP300,MTOR,PPP1R12A,AKT3,VCL,NOS2,CTNNB1,MYH1,NACA,ITGB1,ACTB,PIK3C2G,VIM,MYL1,ITGB2,FLNC,SH2B2,PTGS2 |
| Xenobiotic Metabolism Signaling | 2.96 | 25.70% | MAP2K4,NDST3,MAP3K15,RAF1,MAP3K11,CAMK1,CAMK1D,GCLC,CHST15,CHST2,HS6ST1,CAMK2D,CAMK2A,CYP3A5,PPM1J,MAPK3,PPM1L,CHST11,Map3k7,HS6ST3,HS3ST1,ALDH7A1,HDAC4,MED1,GSTM3,CREBBP,GRIP1,UGT1A1,SULT2A1,HDAC5,PPP2CB,PPP2R1A,SULT1E1,PPP2R4,HSP90AA1,NDST1,HS3ST5,CAMK2G,CHST7,PPP2R2A,HS2ST1,SOD3,PTGES3,EP300,CUL3,HSP90B1,CES1,UST,HSP90AB1,CHST3,HS6ST2,CHST10,NOS2,MAP2K1,ALDH5A1,CAMK2B,ABCB1,MAP3K1,PIK3C2G,UGT8,CHST12,Sult1d1,CHST1,ALDH1L2,CAT,GSTO2,ABCC3,EIF2AK3,SULT1B1,DNAJC7 |
| α-Adrenergic Signaling | 2.95 | 32.20% | RAF1,ADCY4,GNB2L1,PRKAG1,GNB1,GNB4,PHKB,GYS1,MAPK3,GNB1L,MAP2K1,GNG4,GNAS,ITPR2,ADCY3,GNG2,GNAI1,PRKAR2A,ADCY6,PYGB,GNG3,ITPR1,GNAI2,ADCY9,PYGM,ADRA2A,ITPR3,ADRA1A |
| TREM1 Signaling | 2.93 | 33.30% | ITGB1,Naip1 (includes others),ICAM1,IL1RL1,TLR8,ITGA5,CD83,JAK2,STAT3,TLR9,FCGR2B,TLR2,NOD2,MPO,NLRC3,MAPK3,CASP1,TLR7,NLRP4,AKT3,Tlr13,CD86,TLR3,STAT5B,ITGAX |
| 14-3-3-mediated Signaling | 2.89 | 29.90% | MAP2K4,RAF1,TUBA1B,PLCB2,PDIA3,TUBB,YWHAQ,PLCE1,MAPK3,TSC2,PLCB1,AKT3,TUBB4A,GSK3B,MAP2K1,TUBB1,TUBB3,YWHAG,YWHAE,YWHAB,TUBB4B,TUBB2A,TUBA4A,PIK3C2G,YWHAZ,VIM,PLCL2,FOXO1,TUBB6,MAPT,MAPK10,PLCB3,CDKN1B,SFN,ELK1 |
| FXR/RXR Activation | 2.86 | 29.40% | MAP2K4,KNG1,APOB,APOH,VTN,AMBP,C9,C4A/C4B,PON1,SCARB1,LCAT,ITIH4,CYP19A1,FASN,AKT3,SERPINA1,GC,PON3,AGT,TTR,HPX,C3,FETUB,CREBBP,AHSG,PCYOX1,SULT2A1,A1BG,ALB,FOXO1,TF,MAPK10,NR5A2,PLTP,FGA,CLU,APOD |
| RhoA Signaling | 2.84 | 29.50% | LPAR4,ARPC5,PIKFYVE,PPP1CB,ARHGEF1,MYLK,SEPT11,LIMK1,ROCK2,PPP1R12A,IGF1R,SEMA3F,DLC1,MYL3,MYL12A,ACTR2,PLXNA1,SEPT5,ARHGAP6,ARHGEF12,NRP2,ARPC5L,ACTB,MYLPF,SEPT7,TTN,LPAR3,MYL1,MYL9,ROCK1,ARPC1A,LPAR1,ABL2,LPAR5,ACTG1,PIP4K2C |
| P2Y Purigenic Receptor Signaling Pathway | 2.75 | 29.40% | RAF1,PLCB2,ITGA2B,PDIA3,ADCY4,GNB2L1,PRKAG1,EP300,ITGB3,GNB1,GNB4,PLCE1,MAPK3,PLCB1,AKT3,ATF4,GNB1L,MAP2K1,GNG4,GNAS,CREBBP,ADCY3,GNG2,ADCY6,PRKAR2A,GNAI1,PIK3C2G,PLCL2,GNG3,CREB5,ATF2,GNAI2,P2RY2,ADCY9,PLCB3 |
| Unfolded protein response | 2.66 | 35.20% | CALR,SCAP,HSPH1,HSPA9,OS9,ATF6,HSPA2,HSPA1L,MBTPS2,HSPA8,SEL1L,HSP90B1,UBXN4,MBTPS1,ERO1B,VCP,ATF4,CD82,EIF2AK3 |
| Chondroitin Sulfate Degradation (Metazoa) | 2.65 | 53.30% | HYAL2,GM2A,HYAL1,CD44,HEXB,HEXA,GALNS,ARSB |
| Netrin Signaling | 2.64 | 38.50% | UNC5A,RYR2,UNC5B,PRKAR2A,NTN1,PRKAG1,NCK2,PPP3CB,ABLIM3,RYR3,DCC,UNC5D,RYR1,PPP3CA,UNC5C |
| Adipogenesis pathway | 2.62 | 28.40% | FZD10,FZD3,BMPR2,FBXW7,FGFR3,TGFB1,SMO,TBL1XR1,STAT5B,KAT2A,FZD2,GTF2H3,TP53,CCNH,HDAC4,ATG7,HDAC2,FGFR1,HDAC1,FGFR2,SENP2,FZD9,ERCC2,BSCL2,SLC2A4,SIN3A,HDAC5,Kat6b,NR1D2,SIRT2,FZD4,KDM1A,FOXO1,BMP7,FGFRL1,PER2,RBBP4,WNT5A |
| G Protein Signaling Mediated by Tubby | 2.60 | 40.60% | GNB1,GNG4,GNB4,PLCB2,GNAS,GNB2L1,GNG2,PLCB1,PLCB3,JAK2,INSR,GNG3,GNB1L |
| Glycolysis I | 2.60 | 44.00% | PGK1,ENO1,GPI,PGAM1,PKM,ENO2,ALDOA,GAPDH,PFKP,BPGM,ALDOC |
| Gluconeogenesis I | 2.60 | 44.00% | PGK1,ENO1,GPI,PGAM1,ENO2,ALDOA,GAPDH,MDH1,MDH2,BPGM,ALDOC |
| Leukocyte Extravasation Signaling | 2.55 | 26.30% | MAP2K4,MMP16,JAM2,MLLT4,ITGB3,ROCK2,CTNNA2,ITGA3,TXK,DLC1,ITGA4,ITGA6,THY1,ITGA5,BMX,ITGAM,CDH5,CLDN12,JAM3,MAPK10,PECAM1,ITGA1,VAV1,ACTN4,ACTG1,RAP1B,ICAM1,MMP15,CRK,TIMP1,VCL,CTNNB1,MMP12,MMP19,ITGB1,TIMP3,ARHGAP6,VCAM1,ACTB,ITGA2,GNAI1,PIK3C2G,ITGAL,ROCK1,GNAI2,BTK,F11R,ITGB2,EDIL3,CD44,CLDN22,CTNND1 |
| Ovarian Cancer Signaling | 2.53 | 28.20% | RAF1,FZD10,WNT3,FZD3,PRKAG1,VEGFA,RB1,MTOR,WNT7A,MAPK3,SMO,AKT3,WNT4,GSK3B,CTNNB1,BRCA1,FZD2,MAP2K1,EGFR,TP53,GJA1,PMS2,DVL1,PTGS1,PRKAR2A,PIK3C2G,FZD9,APC,SIN3A,FZD4,WNT10A,MSH6,CD44,EDNRA,PTGS2,WNT11,WNT5A |
| Cardiac β-adrenergic Signaling | 2.41 | 27.80% | ENPP6,PPP2R2A,ADCY4,GNB2L1,PPP1CB,PPP1R3A,AKAP7,ATP2A2,PRKAG1,MPPE1,GNB1,GNB4,CACNA1E,PPP1R12A,PPP1R7,PPM1J,PPM1L,GNB1L,GNG4,PDE2A,CACNA1D,GNAS,RYR2,ADCY3,GNG2,PRKAR2A,ADCY6,CACNA1C,GNG3,CACNA1A,PPP2CB,ADCY9,PPP2R1A,AKAP4,PPP2R4,PDE6D,AKAP1 |
| TCA Cycle II (Eukaryotic) | 2.36 | 43.50% | SDHB,CS,ACO2,DLST,DLD,IDH3A,MDH1,FH,MDH2,OGDH |
| Antioxidant Action of Vitamin C | 2.35 | 29.30% | MAP2K4,PLCB2,PDIA3,PLA2R1,JAK2,PLA2G7,SLC2A3,PRDX6,SLC23A2,IKBKB,PLCE1,LCAT,CSF2RA,MAPK3,PLB1,GPLD1,PLCB1,PLA2G4F,CHUK,STAT5B,PLD3,SLC2A1,PLCL2,SLC2A4,PLD4,PLA2G6,MAPK10,PLCB3,GSTO2 |
| Cyclins and Cell Cycle Regulation | 2.33 | 30.80% | TP53,RAF1,CCNH,HDAC4,HDAC2,PPP2R2A,WEE1,HDAC1,SIN3A,HDAC5,CCNB1,RB1,PPP2CB,PPP2R1A,PPP2R4,TGFB1,PPM1J,PPM1L,TGFB2,BTRC,ATR,CDKN1B,GSK3B,CDC25A |
| Dopamine Receptor Signaling | 2.33 | 30.80% | GNAS,PPP2R2A,PPP1R1B,PRL,ADCY4,ADCY3,ADCY6,PRKAR2A,PPP1CB,DRD5,PPP1R3A,SLC18A2,DRD2,PRKAG1,SLC6A3,PPP2CB,ADCY9,PPP2R1A,DRD1,PPP1R12A,PPP2R4,PPP1R7,PPM1J,PPM1L |
| Phospholipases | 2.27 | 32.80% | PLCB2,PLD3,PDIA3,PLA2R1,PLCL2,PLA1A,PLA2G7,PRDX6,PLCH1,PLD4,PLA2G6,PLCE1,LCAT,PLB1,GPLD1,PLCB1,PLCB3,PLA2G4F,LIPG |
| Sphingosine-1-phosphate Signaling | 2.26 | 28.40% | PLCB2,PDIA3,ADCY4,SMPD1,NAAA,RHOG,PLCE1,MAPK3,PDGFRA,CASP1,PLCB1,AKT3,PDGFRB,GNAS,S1PR2,S1PR5,ADCY3,PIK3C2G,GNAI1,ADCY6,PLCL2,SMPD2,PDGFB,ASAH1,ASAH2,GNAI2,S1PR3,ADCY9,S1PR1,PLCB3,PDGFD |
| phagosome formation | 2.26 | 28.40% | MRC1,PLCB2,FN1,PDIA3,VTN,PLA2R1,TLR8,IGHG1,FCGR2B,FCGR1A,ITGA3,PLCE1,RHOG,TLR7,PLCB1,TLR3,ITGA4,ITGB1,FCGR2A,MRC2,ITGA2,PIK3C2G,ITGA5,PLCL2,TLR9,INPP5D,TLR2,SCARA3,PLCB3,Tlr13,Ighg2b |
| Endothelin-1 Signaling | 2.25 | 26.20% | RAF1,PLCB2,PDIA3,ADCY4,PLA2R1,PLA2G7,PRDX6,PLCE1,LCAT,ECE2,PLB1,MAPK3,GPLD1,CASP1,PLCB1,PLA2G4F,ECE1,NOS2,GUCY1B3,PLD3,GNAS,EDNRB,GUCY2D,ITPR2,PTGS1,ADCY3,GNAI1,ADCY6,PIK3C2G,GNAZ,PLCL2,ITPR1,PLD4,GNAI2,PLA2G6,ADCY9,GNAO1,SHC2,ITPR3,MAPK10,PLCB3,EDNRA,PTGER2,PTGS2,GNAL |
| Neuregulin Signaling | 2.21 | 29.50% | RAF1,ADAM17,NRG2,CRK,TMEFF2,ITGA3,MTOR,HSP90B1,HSP90AB1,MAPK3,ERBB4,AKT3,DLG4,STAT5B,MAP2K1,EGFR,ITGA4,ITGB1,ERBB2IP,DCN,ITGA2,ITGA5,ERBB3,HSP90AA1,CDKN1B,ELK1 |
| Inhibition of Matrix Metalloproteases | 2.19 | 35.90% | HSPG2,TIMP3,ADAM17,ADAM12,TIMP1,MMP16,RECK,THBS2,MMP15,ADAM10,MMP12,A2M,LRP1,MMP19 |
| Regulation of the Epithelial-Mesenchymal Transition Pathway | 2.12 | 25.50% | MAP2K4,RAF1,FZD10,ADAM17,WNT3,FZD3,NCSTN,BCL9,JAK2,FGFR3,NOTCH2,WNT7A,TGFB1,HGF,MAPK3,TGFB2,SMO,SMAD4,AKT3,WNT4,GSK3B,CTNNB1,FZD2,MAP2K1,EGFR,PDGFRB,NOTCH3,JAG2,FGFR1,DVL1,TYK2,PIK3C2G,FGFR2,FZD9,STAT3,APC,MET,CDH2,CDH12,FZD4,WNT10A,FGFRL1,PDGFD,JAG1,NOTCH1,WNT11,WNT5A |
| Role of JAK family kinases in IL-6-type Cytokine Signaling | 2.06 | 40.00% | MAP2K4,IL6ST,MAPK3,IL6R,TYK2,MAPK10,OSMR,STAT3,JAK2,STAT5B |
| Relaxin Signaling | 2.05 | 26.70% | ENPP6,RAP1B,ADCY4,GNB2L1,PRKAG1,MPPE1,GNB1,VEGFA,GNB4,MAPK3,AKT3,NOS2,GNB1L,MAP2K1,Gucy2g,GUCY1B3,GNG4,PDE2A,GNAS,GUCY2D,ADCY3,GNG2,ADCY6,PRKAR2A,PIK3C2G,GNAI1,GNAZ,GNG3,GNAI2,ADCY9,NPR1,GNAO1,ELK1,NPR2,GNAL,PDE6D |
| NF-κB Signaling | 2.03 | 25.60% | RAF1,CSNK2A1,TGFBR3,TLR8,BMPR2,EP300,FGFR3,TANK,IKBKB,PDGFRA,IGF1R,TLR7,AKT3,TLR3,GSK3B,CHUK,TRAF5,LTBR,TNFRSF1B,PDGFRB,EGFR,TNFSF11,FLT1,HDAC2,FGFR1,MAP3K1,HDAC1,CREBBP,PIK3C2G,FGFR2,TNFRSF11A,TLR9,IGF2R,TLR2,NTRK2,CARD11,BCL10,NTRK3,BTRC,FGFRL1,INSR,KDR,MAP4K4,IRAK4 |
| Prostate Cancer Signaling | 2.03 | 29.30% | TP53,RAF1,KLK3,CREBBP,PIK3C2G,CREB5,SIN3A,ATF2,EP300,RB1,MTOR,HSP90B1,AR,FOXO1,HSP90AB1,MAPK3,ATF4,AKT3,HSP90AA1,CHUK,CDKN1B,GSK3B,CTNNB1,MAP2K1 |
| IL-1 Signaling | 2.01 | 28.60% | MAP2K4,ADCY4,GNB2L1,PRKAG1,GNB1,IKBKB,GNB4,CHUK,GNB1L,IL1RAP,GNG4,GNAS,ADCY3,MAP3K1,GNG2,GNAI1,PRKAR2A,ADCY6,GNAZ,GNG3,GNAI2,ADCY9,GNAO1,MAPK10,GNAL,IRAK4 |
| FAK Signaling | 1.99 | 28.70% | RAF1,CAPN11,ARHGEF7,CRK,TLN1,PAK1,ITGA3,MAPK3,AKT3,VCL,MAP2K1,EGFR,ITGA4,ITGB1,ACTB,ITGA2,PIK3C2G,ITGA5,GIT2,TLN2,CAPNS1,PAK3,CAPN1,ACTG1,CAPN3 |
| nNOS Signaling in Skeletal Muscle Cells | 1.97 | 46.70% | SNTB2,RYR3,RYR2,DMD,RYR1,DAG1,CAPN3 |
| γ-glutamyl Cycle | 1.97 | 46.70% | GGCT,GCLC,GGT5,GCLM,GGT1,ANPEP,GGT7 |
| PPAR Signaling | 1.88 | 28.00% | RAF1,IL1RL1,EP300,NR2F1,IKBKB,HSP90B1,IL1RL2,HSP90AB1,MAPK3,PDGFRA,CHUK,TNFRSF1B,STAT5B,MAP2K1,IL1RAP,PDGFRB,IL1RAPL2,MED1,CREBBP,PDGFB,HSP90AA1,IL1RAPL1,PTGS2,INSR,PDGFD,MAP4K4 |
| Inhibition of Angiogenesis by TSP1 | 1.88 | 35.30% | MAP2K4,VEGFA,HSPG2,TP53,CD47,TGFB1,THBS1,CD36,MAPK10,AKT3,KDR,GUCY1B3 |
| Antiproliferative Role of Somatostatin Receptor 2 | 1.86 | 30.20% | RAP1B,GNG4,GUCY2D,GNB2L1,GNG2,PIK3C2G,GNG3,GNB1,GNB4,NPR1,MAPK3,SST,CDKN1B,GNB1L,ELK1,NPR2,MAP2K1,Gucy2g,GUCY1B3 |
| Gustation Pathway | 1.83 | 26.40% | ENPP6,TAS2R31,LPAR4,PLCB2,ASIC2,Tas2r102,ADCY4,P2RX1,PANX1,PRKAG1,MPPE1,GNB1,P2RX3,PDE2A,GNAS,ITPR2,P2RX4,ADCY3,GNG2,PRKAR2A,ADCY6,TAS2R7,ITPR1,P2RY2,P2RY13,ADCY9,TAS1R2,SCNN1G,ITPR3,ASIC1,P2RX7,PDE6D |
| Role of CHK Proteins in Cell Cycle Checkpoint Control | 1.82 | 30.90% | TP53,CDC25C,PPP2R2A,PLK1,RFC5,PPP2CB,PPP2R1A,PCNA,PPP2R4,PPM1J,HUS1,PPM1L,TLK1,CLSPN,ATR,BRCA1,CDC25A |
| Amyloid Processing | 1.80 | 31.40% | CSNK2A1,CAPN11,CSNK1A1,CSNK1D,PRKAR2A,NCSTN,BACE1,PRKAG1,APP,CAPNS1,MAPK3,CAPN1,MAPT,AKT3,GSK3B,CAPN3 |
| fMLP Signaling in Neutrophils | 1.79 | 26.90% | RAF1,PLCB2,ARPC5,GNB2L1,GNB1,GNB4,PPP3CB,MAPK3,PLCB1,GNB1L,MAP2K1,PPP3CA,GNG4,ACTR2,GNAS,ARPC5L,ITPR2,GNG2,FPR2,GNAI1,PIK3C2G,ITPR1,GNG3,FPR1,GNAI2,ARPC1A,ITPR3,PLCB3,ELK1 |
| Regulation of Actin-based Motility by Rho | 1.74 | 27.50% | ARPC5,PIKFYVE,PPP1CB,MYLK,LIMK1,PAK1,ITGA3,RHOG,PPP1R12A,MYL3,MYL12A,ITGA4,ITGB1,ACTR2,ARPC5L,MYLPF,ACTB,ITGA2,ITGA5,MYL1,ROCK1,MYL9,ARPC1A,PAK3,PIP4K2C |
| Glucocorticoid Receptor Signaling | 1.74 | 23.30% | MAP2K4,RAF1,POLR2D,CD163,FCGR1A,FGG,IKBKB,TGFB1,MAPK3,SMARCC2,POLR2I,MED1,HSPA9,CREBBP,TAT,STAT3,ERCC2,MED14,HSPA8,BCL2L1,TAF5,MAPK10,HSP90AA1,PBRM1,ICAM1,ARID1A,PRL,POLR2B,JAK2,PTGES3,HSPA1L,PRKAG1,EP300,HSP90B1,AR,POLR2A,HSP90AB1,PPP3CB,FOXO3,TGFB2,AKT3,SMAD4,CHUK,NOS2,STAT5B,MAP2K1,PPP3CA,GTF2H3,AGT,ADRB2,CCNH,VCAM1,ACTB,MAP3K1,PIK3C2G,HSPA2,TAF6L,FKBP4,PTGS2,SMARCC1,ACTL6B,ELK1,A2M,HLTF |
| DNA Methylation and Transcriptional Repression Signaling | 1.73 | 40.00% | HDAC2,MTA1,CHD4,MBD3,HDAC1,DNMT1,SIN3A,RBBP4 |
| Ephrin A Signaling | 1.71 | 31.20% | EFNA2,EFNA3,PIK3C2G,EPHA4,EPHA3,LIMK1,EFNA1,ROCK2,ROCK1,EPHA10,PAK1,EFNA5,ADAM10,EPHA5,VAV1 |
| 2-ketoglutarate Dehydrogenase Complex | 1.69 | 75.00% | DLST,DLD,OGDH |
| Glutathione Redox Reactions II | 1.69 | 75.00% | GSR,TXNDC12,PDIA3 |
| Clathrin-mediated Endocytosis Signaling | 1.68 | 24.30% | CSNK2A1,APOB,EPHB2,PICALM,ARPC5,GAK,F2,ITGB3,VEGFA,PON1,PPP3CB,AMPH,AAK1,SERPINA1,ITGB4,PPP3CA,ITGB5,SNAP91,ITGB1,ACTR2,AP2M1,ARPC5L,ACTB,USP9X,CLTC,PIK3C2G,DNM3,ITGA5,PCYOX1,PDGFB,MET,HSPA8,DNM1,ITGB2,ALB,LDLR,ARPC1A,SYNJ1,TF,TFRC,DNM1L,PDGFD,ACTG1,CLU,APOD |
| Aldosterone Signaling in Epithelial Cells | 1.67 | 25.00% | RAF1,PLCB2,ASIC2,PDIA3,DNAJC6,PIKFYVE,DNAJC10,DNAJA1,SLC9A1,HSPA1L,HSP90B1,PLCE1,HSP90AB1,MAPK3,PLCB1,MAP2K1,ITPR2,HSPH1,SLC12A2,HSPA9,PIK3C2G,HSPD1,ITPR1,PLCL2,HSPA2,DNAJB14,HSPA8,SCNN1G,DNAJC5,HSCB,DNAJB11,ITPR3,HSPA13,ASIC1,PLCB3,HSP90AA1,PIP4K2C,DNAJC7 |
| Melatonin Signaling | 1.67 | 28.60% | MAP2K4,RAF1,PLCB2,PDIA3,PRKAR2A,GNAI1,PLCL2,SLC2A4,PRKAG1,GNAI2,PLCE1,CAMK2A,CAMK2D,MAPK3,GNAO1,PLCB3,PLCB1,MAP2K1,CAMK2B,CAMK2G |
| Regulation of Cellular Mechanics by Calpain Protease | 1.66 | 29.80% | ITGB1,CAPN11,ITGA2,ITGA5,TLN1,RB1,ITGA3,CAPNS1,TLN2,CAPN1,MAPK3,VCL,CDKN1B,ACTN4,CAPN3,EGFR,ITGA4 |
| Ketogenesis | 1.66 | 50.00% | ACAT2,ACAT1,HADHB,HMGCLL1,HADHA |
| Aspartate Degradation II | 1.62 | 57.10% | GOT1,MDH1,MDH2,GOT2 |
| GPCR-Mediated Nutrient Sensing in Enteroendocrine Cells | 1.62 | 27.40% | GNG4,PLCB2,GNAS,ITPR2,PDIA3,ADCY4,ADCY3,GNG2,ADCY6,PRKAR2A,GNAI1,PLCL2,ITPR1,GNG3,PRKAG1,GNAI2,ADCY9,CASR,PLCE1,ITPR3,LPAR5,PLCB3,PLCB1 |
| GM-CSF Signaling | 1.62 | 29.00% | RAF1,GNB2L1,PIK3C2G,STAT3,JAK2,BCL2L1,CAMK2A,CAMK2D,PPP3CB,CSF2RA,MAPK3,AKT3,STAT5B,ELK1,MAP2K1,PPP3CA,CAMK2G,CAMK2B |
| NGF Signaling | 1.61 | 26.20% | MAP2K4,RAP1B,RAF1,MAP3K15,MAP3K11,TRIO,SMPD1,CRK,EP300,ROCK2,IKBKB,RHOG,MAPK3,Map3k7,AKT3,ATF4,CHUK,MAP2K1,TP53,MAP3K1,CREBBP,PIK3C2G,CREB5,SMPD2,ATF2,ROCK1,MAPK10,ELK1 |
| Protein Ubiquitination Pathway | 1.59 | 23.10% | FZR1,USP45,USP20,DNAJC6,UBE2V2,FBXW7,PSMC5,USP7,USP8,SUGT1,USP40,BRCA1,NEDD4L,UBE4B,HSPA9,USP9X,USP19,DNAJB14,USP26,HSPA8,DNAJC5,USP29,UBR1,PSMA5,HSPA13,BAP1,HSP90AA1,USP24,USP12,USP14,CDC20,HLA-A,USP53,USP54,DNAJC10,DNAJA1,HSPA1L,UBE4A,UCHL1,USP3,HSP90B1,HSP90AB1,USP47,PSMD14,HSPH1,HSPD1,USP33,HSPA2,PSME1,USP4,HSCB,PSMD2,DNAJB11,USP37,BTRC,UBA1,PSMC3,UBE2D3,DNAJC7 |
| Ceramide Signaling | 1.59 | 27.50% | MAP2K4,RAF1,S1PR2,S1PR5,PPP2R2A,MAP3K1,PIK3C2G,SMPD1,CERK,SMPD2,S1PR3,PPP2CB,CTSD,PPP2R1A,PPP2R4,PPM1J,MAPK3,PPM1L,S1PR1,AKT3,TNFRSF1B,MAP2K1 |
| D-myo-inositol-5-phosphate Metabolism | 1.57 | 24.80% | Dusp21,PLCB2,DUSP8,PTPN2,PTPN13,NUDT3,PTPN12,PTPRF,PLCH1,PXYLP1,SACM1L,PTPRC,ATP1A1,PLCE1,PPP1R12A,PTPRJ,PTPRO,PPP1R7,PPP1R13B,PLCB1,ALPL,PTPRN,PPP3CA,CDC25A,CDC25C,PPP1R1B,PTPRM,PLD4,MTMR4,SYNJ1,PPP2R4,PPM1H,PLCB3,PIP4K2C,PTPN22,SIRPA |
| CCR3 Signaling in Eosinophils | 1.57 | 25.60% | RAF1,PLCB2,GNB2L1,PPP1CB,MYLK,LIMK1,GNB1,ROCK2,GNB4,PAK1,PPP1R12A,MAPK3,PLCB1,PLA2G4F,GNB1L,MAP2K1,GNG4,GNAS,ITPR2,GNG2,GNAI1,PIK3C2G,ITPR1,GNG3,GNAI2,ROCK1,PLA2G6,PAK3,ITPR3,PLCB3 |
| B Cell Receptor Signaling | 1.55 | 24.10% | MAP2K4,RAP1B,RAF1,MAP3K15,MAP3K11,IGHG1,FCGR2B,EP300,PTPRC,IKBKB,MTOR,CAMK2A,CAMK2D,PPP3CB,MAPK3,CD22,Map3k7,ATF4,AKT3,GSK3B,CHUK,MAP2K1,PPP3CA,CAMK2B,CD19,FCGR2A,MAP3K1,CREBBP,PIK3C2G,CREB5,INPP5D,ATF2,BTK,BCL2L1,FOXO1,SYNJ1,DAPP1,BCL10,VAV1,Ighg2b,ELK1,CAMK2G |
| Isoleucine Degradation I | 1.55 | 42.90% | BCAT1,ACAT2,ACAT1,DLD,HADHB,HADHA |
| Telomerase Signaling | 1.55 | 26.30% | RAF1,PPP2R2A,PTGES3,RB1,HSP90B1,SP1,HSP90AB1,MAPK3,PPM1J,PPM1L,AKT3,MAP2K1,EGFR,IL2RB,TP53,HDAC4,HDAC2,DKC1,HDAC1,PIK3C2G,HDAC5,PPP2CB,PPP2R1A,PPP2R4,TPP1,HSP90AA1 |
| Role of Oct4 in Mammalian Embryonic Stem Cell Pluripotency | 1.53 | 30.40% | TP53,Tdh,REST,PHC1,TDRD7,IGF2BP1,PARP1,NR2F1,RB1,PHB,JARID2,NR5A2,BRCA1,ASH2L |
| MSP-RON Signaling Pathway | 1.53 | 30.40% | F11,TLR2,KLKB1,ITGB2,F12,ITGAM,KLK3,CSF1,ACTB,PIK3C2G,MST1R,JAK2,NOS2,ACTG1 |
| ATM Signaling | 1.52 | 28.80% | TP53,MAP2K4,SMC3,CDC25C,TRIM28,CREBBP,TDP1,CREB5,ATF2,CCNB1,EP300,SMC2,MAPK10,ATF4,TLK1,BRCA1,CDC25A |
| Mouse Embryonic Stem Cell Pluripotency | 1.52 | 26.30% | TP53,IL6ST,RAF1,FZD10,T,FZD3,CREBBP,TYK2,DVL1,PIK3C2G,BMPR2,FZD9,STAT3,JAK2,APC,LIFR,FZD4,MAPK3,SMO,SMAD4,AKT3,GSK3B,CTNNB1,FZD2,MAP2K1 |
| Macropinocytosis Signaling | 1.51 | 27.90% | MRC1,ITGB1,ANKFY1,PIK3C2G,ITGA5,CSF1R,PDGFB,ITGB3,MET,ITGB2,PAK1,ABI1,CSF1,HGF,CD14,ITGB4,ACTN4,PDGFD,ITGB5 |
| Nitric Oxide Signaling in the Cardiovascular System | 1.49 | 26.00% | KNG1,PDE2A,CACNA1D,FLT1,GUCY2D,ITPR2,RYR2,PIK3C2G,PRKAR2A,SLC7A1,CACNA1C,ITPR1,ATP2A2,PRKAG1,CACNA1A,VEGFA,HSP90B1,CACNA1E,HSP90AB1,MAPK3,ITPR3,AKT3,HSP90AA1,KDR,MAP2K1,GUCY1B3 |
| Role of NFAT in Regulation of the Immune Response | 1.48 | 24.00% | RAF1,PLCB2,CD4,GNB2L1,CSNK1A1,FCGR2B,FCGR1A,GNB1,IKBKB,GNB4,PPP3CB,MAPK3,XPO1,AKT3,PLCB1,GSK3B,CHUK,GNB1L,MAP2K1,PPP3CA,GNG4,GNAS,ITPR2,FCGR2A,GNG2,GNAI1,CSNK1D,PIK3C2G,GNAZ,ITPR1,GNG3,ATF2,GNAI2,BTK,GNAO1,ITPR3,CD86,PLCB3,HLA-DRB5,GATA4,GNAL |
| Choline Degradation I | 1.48 | 100.00% | CHDH,ALDH7A1 |
| Palmitate Biosynthesis I (Animals) | 1.48 | 100.00% | OXSM,FASN |
| Fatty Acid Biosynthesis Initiation II | 1.48 | 100.00% | OXSM,FASN |
| Oncostatin M Signaling | 1.48 | 32.40% | IL6ST,RAF1,TIMP3,MAPK3,TYK2,OSMR,STAT3,JAK2,STAT5B,ELK1,MAP2K1 |
| Role of JAK2 in Hormone-like Cytokine Signaling | 1.48 | 32.40% | PRL,SH2B2,TYK2,IRS2,SOCS4,STAT3,JAK2,STAT5B,HLTF,SIRPA,SH2B1 |
| NF-κB Activation by Viruses | 1.48 | 27.40% | ITGB1,RAF1,CD4,MAP3K1,ITGA2,PIK3C2G,ITGA6,ITGA5,ITGAL,ITGB3,ITGB2,IKBKB,ITGA3,MAPK3,ITGAV,AKT3,ITGA1,CHUK,ITGB5,ITGA4 |
| Sonic Hedgehog Signaling | 1.47 | 33.30% | GLIS1,PTCH1,PRKAR2A,SMO,HHIP,GSK3B,GLI1,PRKAG1,CCNB1,DYRK1A |
| Tec Kinase Signaling | 1.47 | 24.20% | MAP2K4,GTF2I,GNB2L1,JAK2,GNB1,GNB4,YES1,ITGA3,PAK1,RHOG,TXK,GNB1L,STAT5B,ITGA4,GNG4,ITGB1,TNFRSF21,STAT6,GNAS,ACTB,TYK2,GNG2,ITGA2,GNAI1,PIK3C2G,ITGA5,BMX,STAT3,GNAZ,GNG3,BTK,GNAI2,PAK3,GNAO1,MAPK10,VAV1,ACTG1,GNAL |
| D-myo-inositol (1,4,5)-Trisphosphate Biosynthesis | 1.47 | 34.60% | PLD4,PLCB2,PLCE1,PI4K2A,PIKFYVE,PLCB1,PLCB3,PLCH1,PIP4K2C |
| nNOS Signaling in Neurons | 1.45 | 29.80% | GRIN1,GRIN2B,GRIN2A,CAPNS1,CAMK2A,PPP3CB,CAPN11,GRIN2C,CAPN1,GRIN2D,DLG2,DLG4,PPP3CA,CAPN3 |
| Hereditary Breast Cancer Signaling | 1.45 | 24.80% | FANCM,PBRM1,ARID1A,POLR2D,POLR2B,EP300,RB1,FANCB,POLR2A,AKT3,SMARCC2,BRCA1,TP53,POLR2I,CDC25C,PMS2,HDAC4,HDAC2,WEE1,ACTB,CREBBP,HDAC1,PIK3C2G,RFC5,HDAC5,CCNB1,MSH6,ATR,SMARCC1,ACTL6B,SFN,HLTF |
| Granulocyte Adhesion and Diapedesis | 1.44 | 23.70% | ICAM1,IL1RL1,MMP16,MMP15,HRH3,ITGB3,HRH1,ITGA3,ICAM2,IL1RL2,CCL28,TNFRSF1B,MMP12,IL1RAP,MMP19,ITGA4,CSF3R,ITGB1,VCAM1,HRH2,IL1RAPL2,ITGA2,GNAI1,FPR2,ITGA6,ITGA5,THY1,ITGAL,GLG1,C5,FPR1,GNAI2,ITGB2,ITGAM,CLDN12,SELP,CDH5,JAM3,PECAM1,ITGA1,IL1RAPL1,CLDN22 |
| Pancreatic Adenocarcinoma Signaling | 1.43 | 25.50% | MAP2K4,RAF1,RALA,JAK2,VEGFA,RB1,TGFB1,MAPK3,GPLD1,TGFB2,SMAD4,AKT3,MAP2K1,EGFR,TP53,PLD3,TYK2,PIK3C2G,STAT3,SIN3A,PLD4,BCL2L1,MAPK10,CDKN1B,PTGS2,ELK1,NOTCH1 |
| Leptin Signaling in Obesity | 1.42 | 27.00% | PLCB2,GNAS,PDIA3,ADCY4,ADCY3,PIK3C2G,ADCY6,PRKAR2A,JAK2,PLCL2,STAT3,PRKAG1,ADCY9,PLCE1,FOXO1,MAPK3,PLCB3,PLCB1,AKT3,MAP2K1 |
| IGF-1 Signaling | 1.41 | 25.80% | RAF1,CSNK2A1,YWHAG,YWHAE,YWHAB,PIK3C2G,YWHAZ,PRKAR2A,SOCS4,JAK2,IGFBP7,STAT3,PRKAG1,GRB10,YWHAQ,NOV,FOXO1,MAPK3,FOXO3,IGF1R,AKT3,IRS2,SFN,ELK1,MAP2K1 |
| Androgen Signaling | 1.41 | 25.20% | POLR2D,GNB2L1,POLR2B,PRKAG1,EP300,GNB1,GNB4,POLR2A,AR,MAPK3,GNB1L,GTF2H3,POLR2I,GNG4,CALR,CCNH,GNAS,CREBBP,GNG2,GNAI1,PRKAR2A,GNAZ,GNG3,ERCC2,GNAI2,GNAO1,HSP90AA1,GNAL |
| Superoxide Radicals Degradation | 1.39 | 50.00% | SOD2,CAT,SOD1,SOD3 |
| Fcγ Receptor-mediated Phagocytosis in Macrophages and Monocytes | 1.38 | 25.80% | ACTR2,PLD3,FCGR2A,ARPC5L,ACTB,ARPC5,PIK3C2G,CRK,TLN1,FCGR1A,INPP5D,NCK2,MYO5A,PLD4,PLA2G6,YES1,PAK1,TLN2,ARPC1A,MAPK3,GPLD1,AKT3,VAV1,ACTG1 |
| DNA damage-induced 14-3-3σ Signaling | 1.37 | 36.80% | TP53,HUS1,AKT3,ATR,SFN,BRCA1,CCNB1 |
| p53 Signaling | 1.37 | 25.50% | ST13,EP300,RB1,THBS1,PPP1R13B,CCNK,ADCK3,ADGRB1,AKT3,GSK3B,CTNNB1,BRCA1,TP53,PRKDC,TP63,MED1,TOPBP1,HDAC1,CSNK1D,PIK3C2G,SERPINE2,BCL2L1,PCNA,ATR,SFN |
| Virus Entry via Endocytic Pathways | 1.35 | 25.80% | ITGB1,AP2M1,HLA-A,ACTB,CLTC,ITGA2,PIK3C2G,ITGA6,ITGA5,ITGAL,ITGB3,FOLR1,DNM1,ITGB2,ITGA3,FLNC,TFRC,ITGA1,ITGB4,CXADR,ACTG1,ITGB5,ITGA4 |
| Crosstalk between Dendritic Cells and Natural Killer Cells | 1.35 | 25.80% | HLA-A,ACTB,CD69,TLN1,CD83,TLR9,ITGAL,KIR3DL3,CAMK2A,CAMK2D,TLN2,TLR7,CD86,LTBR,TLR3,TNFRSF1B,ACTG1,HLA-DRB5,HLA-E,PVRL2,CAMK2B,CAMK2G,IL2RB |
| Creatine-phosphate Biosynthesis | 1.35 | 60.00% | CKM,MAP4K4,CKMT1A/CKMT1B |
| Chemokine Signaling | 1.33 | 26.80% | RAF1,PLCB2,CAMK1,CAMK1D,PIK3C2G,GNAI1,PPP1CB,LIMK1,ROCK2,GNAI2,CAMK2A,CAMK2D,PPP1R12A,MAPK3,PLCB3,PLCB1,MAP2K1,CAMK2G,CAMK2B |
| Insulin Receptor Signaling | 1.32 | 24.20% | RAF1,ASIC2,PPP1CB,PPP1R3A,CRK,JAK2,PRKAG1,PTPRF,MTOR,GYS1,PPP1R12A,PPP1R7,MAPK3,FOXO3,TSC2,AKT3,IRS2,GSK3B,MAP2K1,PIK3C2G,PRKAR2A,ACLY,VAMP2,SLC2A4,INPP5D,GRB10,SCNN1G,FOXO1,SYNJ1,SH2B2,ASIC1,INSR |
| LPS/IL-1 Mediated Inhibition of RXR Function | 1.31 | 22.70% | MAP2K4,NDST3,CPT1B,CHST15,ABCA1,CHST2,HS6ST1,SCARB1,IL1RL2,CYP3A5,CHST11,XPO1,HS6ST3,HS3ST1,IL1RAP,ALDH7A1,GSTM3,ACSL6,SULT2A1,SULT1E1,CD14,IL1RAPL1,PLTP,ACOX3,NDST1,HS3ST5,CHST7,IL1RL1,HS2ST1,SOD3,UST,CHST3,HS6ST2,FABP5,CHST10,TNFRSF1B,ALDH5A1,ABCB1,IL1RAPL2,MAP3K1,CHST12,Sult1d1,CHST1,ALDH1L2,CAT,NR5A2,GSTO2,ABCC3,SULT1B1,ACSL1 |
| Rac Signaling | 1.30 | 25.00% | MAP2K4,ITGB1,ACTR2,RAF1,MAP3K11,ARPC5L,MAP3K1,ARPC5,ITGA2,PIK3C2G,PIKFYVE,ITGA5,IQGAP1,LIMK1,ITGA3,PAK1,ARPC1A,CYFIP2,PAK3,CYFIP1,MAPK3,CD44,ELK1,MAP2K1,PIP4K2C,ITGA4 |
| Glutaryl-CoA Degradation | 1.30 | 41.70% | ACAT2,ACAT1,HADHB,HADHA,GCDH |
