# Supplementary material for: In-depth mapping of the mouse brain N-glycoproteome reveals widespread N-glycosylation of diverse brain proteins
Source: Oncotarget. 2016 May 31;7(25):38796–809. doi: 10.18632/oncotarget.9737 (PMC5122430; doi:10.18632/oncotarget.9737)
Supplement: Supplementary file 6 [file oncotarget-07-38796-s006.docx]

| Supplementary Table S7: Canonical pathways that the identified N-glycoproteins were associated with in nervous system signaling | | | |
| --- | --- | --- | --- |
| **Ingenuity canonical pathways** | **–log(*p*-value)** | **Ratio** | **Molecules** |
| Axonal Guidance Signaling | 32.30 | 42.60% | SLIT3,DPYSL2,KLC1,RAF1,TUBA1B,ADAMTS8,ECEL1,Adam26a/Adam26b,ARPC5,SEMA4F,NTN1,ADAMTS2,GNB1,VEGFA,NCK2,GNB4,PAK1,ITGA3,EPHB1,ECE2,ABLIM3,Adam24,PLCB1,PLXNB2,SRGAP2,PLXNB3,MYL3,ITGA4,EFNA2,ACTR2,SEMA5A,PTCH1,TUBB2A,ITGA5,L1CAM,PLCL2,MET,MYL9,SRGAP3,ARPC1A,Bmp8b,RTN4,PDGFD,GNAL,NRP1,RAP1B,ADAM17,LRRC4C,PLXNA3,UNC5A,PDIA3,BDNF,ARHGEF7,SEMA6A,EPHA4,CRK,PLXNA2,EFNB2,WNT7A,MYSM1,EFNA5,DCC,TUBB4A,PSMD14,LNPEP,ROBO2,GNB1L,PPP3CA,UNC5C,MYL12A,BMP1,GNG4,ITGB1,TUBB1,PLXNA1,SEMA3G,EPHB4,ADAM2,NRP2,TUBB4B,MYLPF,ITGA2,PIK3C2G,EPHA3,SLIT2,MYL1,EFNA1,SEMA3A,SEMA4D,PAK3,MAG,NTRK3,ADAM10,EPHA5,SEMA4G,BMP7,SEMA3C,SEMA7A,NTN3,PLCB2,WNT3,EPHB2,FZD3,UNC5B,GNB2L1,SEMA6B,ADAM11,TUBB,LIMK1,ROCK2,SEMA6D,PLCE1,SEMA3D,MAPK3,UNC5D,ADAM23,WNT4,EFNB3,GSK3B,RTN4R,FZD2,ADAMTS4,TUBB3,GNG2,FZD9,GNAZ,HHIP,DPYSL5,GNG3,PDGFB,BMP10,HERC2,ADAM12,GNAO1,PLCB3,FZD10,ENPEP,ADAM22,ADAMTS7,SLIT1,ROBO1,ADAM21,SEMA4C,PRKAG1,EPHB6,NTNG1,PPP3CB,GLIS1,EFNB1,MKNK1,SMO,PLXNB1,AKT3,SEMA4A,SHANK2,MAP2K1,SEMA3F,ACE,SEMA3E,GNAS,PLXNC1,ARHGEF12,ARPC5L,ADAM15,C9orf3,GNAI1,TUBA4A,PRKAR2A,EFNA3,PLXND1,GIT1,ROCK1,GNAI2,EPHA10,NTRK2,FZD4,TUBB6,WNT10A,LINGO1,EPHB3,GLI1,SEMA4B,ADAM9,WNT11,WNT5A |
| Glutamate Receptor Signaling | 16.00 | 68.40% | GRM2,GRIN2A,SLC1A4,HOMER2,SLC17A6,GRM3,GRIA1,GRIN2D,GRID2,GRID1,SLC17A2,SLC1A3,GRIA4,GNB1,GRIK5,GRIN2C,HOMER1,DLG4,GRIK2,GRIK1,GRIN1,GRIN2B,SLC1A6,GRM1,GRM8,GLS,GRIK3,GNG2,GRIA2,GRIP1,SLC1A1,GRM4,GRM5,GRM7,GRIK4,SLC17A7,SLC1A2,GLUL,GRIA3 |
| Ephrin Receptor Signaling | 11.60 | 40.80% | RAF1,GRIN2A,EPHB2,ARPC5,GNB2L1,LIMK1,NCK2,VEGFA,ROCK2,GNB1,GNB4,EPHB1,ITGA3,PAK1,GRIN2C,MAPK3,ATF4,EFNB3,ITGA4,EFNA2,ACTR2,GNG2,CREBBP,ITGA5,GNG3,STAT3,GNAZ,CREB5,PDGFB,ATF2,ARPC1A,GNAO1,PDGFD,GNAL,RAP1B,PTPN13,GRIN2D,EPHA4,CRK,JAK2,EP300,EPHB6,EFNB2,EFNA5,EFNB1,AKT3,GNB1L,MAP2K1,ITGB1,GNG4,GRIN2B,GRIN1,EPHB4,GNAS,ANGPT1,ARPC5L,ITGA2,GNAI1,PIK3C2G,EFNA3,EPHA3,EFNA1,GNAI2,ROCK1,EPHA10,ABI1,PAK3,ADAM10,EPHA5,EPHB3,MAP4K4 |
| CREB Signaling in Neurons | 11.60 | 40.90% | RAF1,PLCB2,GRIN2A,POLR2D,GRM3,ADCY4,GNB2L1,GRIA4,GNB1,GNB4,GRIK5,PLCE1,CAMK2A,CAMK2D,GRIN2C,MAPK3,ATF4,PLCB1,GRIK1,POLR2I,ITPR2,GRM1,GRM8,CREBBP,GNG2,GRIA2,GNG3,ITPR1,GRM4,GNAZ,PLCL2,CREB5,ATF2,GRM7,ADCY9,ITPR3,GNAO1,PLCB3,GNAL,CAMK2G,GRM2,PDIA3,GRID1,GRID2,GRIN2D,GRIA1,POLR2B,PRKAG1,EP300,POLR2A,AKT3,GRIK2,GNB1L,MAP2K1,CAMK2B,GNG4,GRIN2B,GRIN1,GNAS,GRIK3,ADCY3,ADCY6,PRKAR2A,GNAI1,PIK3C2G,GRM5,GNAI2,GRIK4,ELK1,GRIA3 |
| Synaptic Long Term Depression | 8.21 | 38.70% | RAF1,PLCB2,GRM3,PLA2R1,PLA2G7,GRIA4,PLCE1,LCAT,PPM1J,MAPK3,RYR3,PPM1L,PLCB1,PLA2G4F,Gucy2g,GUCY1B3,ITPR2,GRM1,GRM8,GUCY2D,GRIA2,GRM4,PLCL2,GNAZ,ITPR1,GRM7,PLA2G6,PPP2CB,PPP2R1A,PPP2R4,NPR1,ITPR3,GNAO1,PLCB3,NPR2,GNAL,GRM2,PPP2R2A,PDIA3,GRID1,GRID2,GRIA1,PRDX6,PLB1,IGF1R,RYR1,NOS2,MAP2K1,GNAS,RYR2,GNAI1,CRHR1,GRM5,GNAI2,GRIA3 |
| GABA Receptor Signaling | 8.18 | 49.30% | GABRA5,UBQLN1,GABRA4,ADCY4,GPHN,SLC6A13,GABRB2,NSF,GABRG3,GABRB3,GABRG1,GPR37,GABRA6,GABRB1,GABRA1,GABRD,GABRA2,GABRA3,ALDH5A1,AP2M1,GNAS,ADCY3,ADCY6,GABBR1,KCNH2,DNM1,ADCY9,GABBR2,SLC6A11,GABRG2,GAD1,SLC6A1,SLC6A12 |
| Synaptic Long Term Potentiation | 7.42 | 39.50% | RAP1B,RAF1,GRM2,PLCB2,GRIN2A,PDIA3,GRM3,GRIA1,GRIN2D,PPP1CB,PPP1R3A,GRIA4,PRKAG1,EP300,CAMK2A,CAMK2D,PLCE1,PPP1R12A,PPP3CB,GRIN2C,PPP1R7,MAPK3,ATF4,PLCB1,MAP2K1,PPP3CA,CAMK2B,GRIN2B,GRIN1,ITPR2,GRM1,GRM8,CREBBP,PRKAR2A,GRIA2,CACNA1C,GRM4,PLCL2,ITPR1,CREB5,ATF2,GRM5,GRM7,ITPR3,PLCB3,GRIA3,CAMK2G |
| Ephrin B Signaling | 7.03 | 45.20% | EPHB2,GNB2L1,HNRNPK,LIMK1,GNB1,EPHB6,ROCK2,NCK2,EFNB2,GNB4,EPHB1,PAK1,MAPK3,EFNB1,ITSN2,EFNB3,CTNNB1,GNB1L,GNG4,EPHB4,GNAS,GNG2,GNAI1,GNAZ,GNG3,GNAI2,ROCK1,ABI1,GNAO1,CAP1,EPHB3,VAV1,GNAL |
| Neuropathic Pain Signaling In Dorsal Horn Neurons | 6.59 | 40.00% | GRIN2A,GRM2,PLCB2,CAMK1,CAMK1D,PDIA3,BDNF,GRM3,GRIA1,GRIN2D,GRIA4,PRKAG1,CAMK2A,CAMK2D,PLCE1,GPR37,GRIN2C,MAPK3,PLCB1,CAMK2B,GRIN1,GRIN2B,ITPR2,GRM8,GRM1,PRKAR2A,PIK3C2G,GRIA2,GRM4,PLCL2,ITPR1,KCNH2,GRM5,GRM7,NTRK2,ITPR3,PLCB3,ELK1,GRIA3,CAMK2G |
| Amyotrophic Lateral Sclerosis Signaling | 6.38 | 39.80% | GRIN2A,Naip1 (includes others),CAPN11,GRIA1,GRIN2D,GRID2,GRID1,GRIA4,VEGFA,PAK1,GRIK5,CACNA1E,HECW1,GRIN2C,CASP1,AKT3,GRIK2,PPP3CA,GRIK1,TP53,GRIN1,GRIN2B,CACNA1D,GRIK3,PIK3C2G,GRIA2,CACNA1C,SOD1,CACNA1A,CCS,BCL2L1,CAPNS1,GRIK4,CAPN1,CAT,SLC1A2,GLUL,CAPN3,GRIA3 |
| Huntington’s Disease Signaling | 5.81 | 31.00% | MAP2K4,PLCB2,POLR2D,REST,GNB2L1,NAPG,GNB1,GNB4,NSF,CTSD,MAPK3,ATF4,DLG4,PLCB1,POLR2I,TP53,HDAC4,HDAC2,GRM1,CLTC,HSPA9,GNG2,CREBBP,DNM3,GNG3,ITPR1,CREB5,STX1A,RPH3A,GPAA1,ATF2,HDAC5,HSPA8,BCL2L1,DNAJC5,CACNA1B,CAPN1,PENK,PLCB3,CAPN3,SDHB,CAPN11,BDNF,PACSIN1,POLR2B,HSPA1L,EP300,MTOR,POLR2A,SP1,CASP1,IGF1R,AKT3,GOSR1,GNB1L,NAPB,EGFR,GNG4,GRIN2B,GLS,HDAC1,PIK3C2G,HSPA2,SNAP25,SIN3A,GRM5,DNM1,PSME1,CAPNS1,DNM1L,GOSR2 |
| CDK5 Signaling | 5.77 | 38.40% | RAF1,PPP2R2A,BDNF,ADCY4,PPP1CB,PPP1R3A,PRKAG1,LAMC1,ITGA3,DRD1,PPP1R12A,PPP1R7,MAPK3,PPM1J,PPM1L,LAMA1,LAMB1,MAP2K1,ITGB1,LAMA5,GNAS,PPP1R1B,ADCY3,ITGA2,PRKAR2A,ITGA6,ADCY6,DRD5,CACNA1A,PPP2CB,ADCY9,PPP2R1A,NTRK2,PPP2R4,MAPT,CAPN1,MAPK10,GNAL |
| Dopamine-DARPP32 Feedback in cAMP Signaling | 5.70 | 33.50% | PLCB2,GRIN2A,ADCY4,CSNK1A1,PPP1R3A,ATP2A2,PLCE1,CACNA1E,GRIN2C,PPP1R7,PPM1J,PPM1L,ATF4,PLCB1,GUCY1B3,KCNJ8,ITPR2,PPP1R1B,CREBBP,CSNK1D,CACNA1C,ITPR1,PLCL2,CREB5,DRD2,CACNA1A,ATF2,PPP2CB,ADCY9,PPP2R1A,PPP2R4,ITPR3,PLCB3,PPP2R2A,PDIA3,GRIN2D,PPP1CB,PRKAG1,EP300,PPP1R12A,DRD1,PPP3CB,PPP3CA,GRIN2B,GRIN1,GNAS,CACNA1D,ADCY3,ADCY6,PRKAR2A,GNAI1,DRD5,GNAI2,CAMKK1 |
| Agrin Interactions at Neuromuscular Junction | 4.95 | 40.60% | MAP2K4,NRG2,ARHGEF7,ITGB3,LAMC1,ITGA3,PAK1,MAPK3,ERBB4,LAMB1,DAG1,EGFR,ITGA4,ITGB1,ACTB,DVL1,ITGA2,LAMA2,ITGA6,ITGA5,ERBB3,ITGAL,ITGB2,PAK3,MAPK10,ITGA1,AGRN,ACTG1 |
| Circadian Rhythm Signaling | 4.84 | 51.50% | GRIN2B,AVP,GRIN1,GRIN2A,GRIN2D,CREBBP,CSNK1D,VIP,CREB5,ATF2,EP300,ADCYAP1R1,NR1D1,GRIN2C,ATF4,CRY1,PER2 |
| GNRH Signaling | 3.90 | 31.80% | MAP2K4,RAF1,MAP3K15,PLCB2,MAP3K11,ADCY4,PRKAG1,EP300,PAK1,CAMK2D,CAMK2A,MAPK3,Map3k7,PLCB1,ATF4,MAP2K1,EGFR,CAMK2B,GNAS,ITPR2,CREBBP,ADCY3,MAP3K1,ADCY6,PRKAR2A,GNAI1,DNM3,ITPR1,CREB5,ATF2,GNAI2,DNM1,ADCY9,PAK3,ITPR3,MAPK10,PLCB3,DNM1L,ELK1,GNRHR,CAMK2G |
| GPCR-Mediated Integration of Enteroendocrine Signaling Exemplified by an L Cell | 3.76 | 36.60% | PLCB2,PDIA3,NMB,ADCY4,PRKAG1,PLCE1,PLCB1,SST,ADRB2,CCKAR,GNAS,ITPR2,ADCY3,GNAI1,PRKAR2A,ADCY6,PLCL2,ITPR1,VIP,GNAI2,ADCY9,GLP1R,ITPR3,NPY2R,PLCB3,GALR1 |
| Semaphorin Signaling in Neurons | 3.69 | 39.60% | DPYSL2,ITGB1,PLXNA1,ARHGEF12,DPYSL3,DPYSL4,DPYSL5,LIMK1,ROCK2,ROCK1,MET,SEMA3A,CRMP1,PAK1,RHOG,SEMA4D,PAK3,MAPK3,PLXNB1,SEMA7A,NRP1 |
| Neuroprotective Role of THOP1 in Alzheimer’s Disease | 3.52 | 42.50% | MME,KNG1,YWHAE,HLA-A,PRKAR2A,SERPINA3,IDE,PRKAG1,APP,PLG,ECE2,MAPT,SST,ECE1,HLA-E,ACE,AGT |
| Reelin Signaling in Neurons | 3.32 | 34.20% | MAP2K4,MAP3K11,ARHGEF1,ITGB3,ITGA3,YES1,GSK3B,ITGA4,ITGB1,ARHGEF12,CNR1,ITGA2,ITGA6,PIK3C2G,ITGA5,MAPK8IP3,RELN,ITGAL,APP,ITGB2,MAPT,MAPK10,ITGA1,LRP8,PAFAH1B1,ARHGEF10,DCX |
| Netrin Signaling | 2.64 | 38.50% | UNC5A,RYR2,UNC5B,PRKAR2A,NTN1,PRKAG1,NCK2,PPP3CB,ABLIM3,RYR3,DCC,UNC5D,RYR1,PPP3CA,UNC5C |
| Dopamine Receptor Signaling | 2.33 | 30.80% | GNAS,PPP2R2A,PPP1R1B,PRL,ADCY4,ADCY3,ADCY6,PRKAR2A,PPP1CB,DRD5,PPP1R3A,SLC18A2,DRD2,PRKAG1,SLC6A3,PPP2CB,ADCY9,PPP2R1A,DRD1,PPP1R12A,PPP2R4,PPP1R7,PPM1J,PPM1L |
| Neuregulin Signaling | 2.21 | 29.50% | RAF1,ADAM17,NRG2,CRK,TMEFF2,ITGA3,MTOR,HSP90B1,HSP90AB1,MAPK3,ERBB4,AKT3,DLG4,STAT5B,MAP2K1,EGFR,ITGA4,ITGB1,ERBB2IP,DCN,ITGA2,ITGA5,ERBB3,HSP90AA1,CDKN1B,ELK1 |
| Gustation Pathway | 1.83 | 26.40% | ENPP6,TAS2R31,LPAR4,PLCB2,ASIC2,Tas2r102,ADCY4,P2RX1,PANX1,PRKAG1,MPPE1,GNB1,P2RX3,PDE2A,GNAS,ITPR2,P2RX4,ADCY3,GNG2,PRKAR2A,ADCY6,TAS2R7,ITPR1,P2RY2,P2RY13,ADCY9,TAS1R2,SCNN1G,ITPR3,ASIC1,P2RX7,PDE6D |
| Amyloid Processing | 1.80 | 31.40% | CSNK2A1,CAPN11,CSNK1A1,CSNK1D,PRKAR2A,NCSTN,BACE1,PRKAG1,APP,CAPNS1,MAPK3,CAPN1,MAPT,AKT3,GSK3B,CAPN3 |
| Regulation of Actin-based Motility by Rho | 1.74 | 27.50% | ARPC5,PIKFYVE,PPP1CB,MYLK,LIMK1,PAK1,ITGA3,RHOG,PPP1R12A,MYL3,MYL12A,ITGA4,ITGB1,ACTR2,ARPC5L,MYLPF,ACTB,ITGA2,ITGA5,MYL1,ROCK1,MYL9,ARPC1A,PAK3,PIP4K2C |
| Ephrin A Signaling | 1.71 | 31.20% | EFNA2,EFNA3,PIK3C2G,EPHA4,EPHA3,LIMK1,EFNA1,ROCK2,ROCK1,EPHA10,PAK1,EFNA5,ADAM10,EPHA5,VAV1 |
| Melatonin Signaling | 1.67 | 28.60% | MAP2K4,RAF1,PLCB2,PDIA3,PRKAR2A,GNAI1,PLCL2,SLC2A4,PRKAG1,GNAI2,PLCE1,CAMK2A,CAMK2D,MAPK3,GNAO1,PLCB3,PLCB1,MAP2K1,CAMK2B,CAMK2G |
| GPCR-Mediated Nutrient Sensing in Enteroendocrine Cells | 1.62 | 27.40% | GNG4,PLCB2,GNAS,ITPR2,PDIA3,ADCY4,ADCY3,GNG2,ADCY6,PRKAR2A,GNAI1,PLCL2,ITPR1,GNG3,PRKAG1,GNAI2,ADCY9,CASR,PLCE1,ITPR3,LPAR5,PLCB3,PLCB1 |
| NGF Signaling | 1.61 | 26.20% | MAP2K4,RAP1B,RAF1,MAP3K15,MAP3K11,TRIO,SMPD1,CRK,EP300,ROCK2,IKBKB,RHOG,MAPK3,Map3k7,AKT3,ATF4,CHUK,MAP2K1,TP53,MAP3K1,CREBBP,PIK3C2G,CREB5,SMPD2,ATF2,ROCK1,MAPK10,ELK1 |
| nNOS Signaling in Neurons | 1.45 | 29.80% | GRIN1,GRIN2B,GRIN2A,CAPNS1,CAMK2A,PPP3CB,CAPN11,GRIN2C,CAPN1,GRIN2D,DLG2,DLG4,PPP3CA,CAPN3 |
