# Supplementary material for: In-depth mapping of the mouse brain N-glycoproteome reveals widespread N-glycosylation of diverse brain proteins
Source: Oncotarget. 2016 May 31;7(25):38796–809. doi: 10.18632/oncotarget.9737 (PMC5122430; doi:10.18632/oncotarget.9737)
Supplement: Supplementary file 9 [file oncotarget-07-38796-s009.docx]

| Supplementary Table S10: Glutamate receptors (GluRs) identified in this study | | | | | | |
| --- | --- | --- | --- | --- | --- | --- |
| **ID** | **Symbol** | **Entrez Gene Name** | **Location** | **Type(s)** | **Biomarker Application(s)** | **Drug(s)** |
| P23818 | GRIA1 | glutamate receptor, ionotropic, AMPA 1 | Plasma Membrane | ion channel |  | talampanel, farampator, LY451395, tezampanel, perampanel, sevoflurane, isoflurane, desflurane, methoxyflurane, enflurane |
| P23819 | GRIA2 | glutamate receptor, ionotropic, AMPA 2 | Plasma Membrane | ion channel |  | talampanel, farampator, LY451395, tezampanel |
| Q9Z2W9 | GRIA3 | glutamate receptor, ionotropic, AMPA 3 | Plasma Membrane | ion channel |  | talampanel, farampator, LY451395, tezampanel |
| Q9Z2W8 | GRIA4 | glutamate receptor, ionotropic, AMPA 4 | Plasma Membrane | ion channel |  | talampanel, farampator, LY451395, tezampanel |
| Q61627 | GRID1 | glutamate receptor, ionotropic, delta 1 | Plasma Membrane | ion channel |  |  |
| Q61625 | GRID2 | glutamate receptor, ionotropic, delta 2 | Plasma Membrane | ion channel |  |  |
| Q60934 | GRIK1 | glutamate receptor, ionotropic, kainate 1 | Plasma Membrane | ion channel |  | phentermine/topiramate, topiramate |
| P39087 | GRIK2 | glutamate receptor, ionotropic, kainate 2 | Plasma Membrane | ion channel |  |  |
| B1AS29 | GRIK3 | glutamate receptor, ionotropic, kainate 3 | Plasma Membrane | ion channel |  |  |
| Q8BMF5 | GRIK4 | glutamate receptor, ionotropic, kainate 4 | Plasma Membrane | ion channel |  |  |
| Q61626 | GRIK5 | glutamate receptor, ionotropic, kainate 5 | Plasma Membrane | ion channel |  |  |
| P35438 | GRIN1 | glutamate receptor, ionotropic, N-methyl D-aspartate 1 | Plasma Membrane | ion channel | unspecified application | dextromethorphan/morphine, neramexane, bicifadine, delucemine, nebostinel, besonprodil, UK-240455, dextromethorphan/quinidine, ketamine, felbamate, ifenprodil, memantine, orphenadrine, cycloserine, aspirin/caffeine/orphenadrine, N-(2-indanyl)glycinamide, dextromethorphan, acamprosate, brompheniramine/dextromethorphan/pseudoephedrine, chlorpheniramine/dextromethorphan/phenylephrine, carbinoxamine/dextromethorphan/pseudoephedrine, dextromethorphan/promethazine, agmatine, 1-aminocyclopropane-1-carboxylic acid |
| P35436 | GRIN2A | glutamate receptor, ionotropic, N-methyl D-aspartate 2A | Plasma Membrane | ion channel | unspecified application | dextromethorphan/morphine, neramexane, bicifadine, delucemine, nebostinel, besonprodil, UK-240455, tenocyclidine, dextromethorphan/quinidine, donepezil/memantine, ketamine, felbamate, memantine, orphenadrine, cycloserine, N-(2-indanyl)glycinamide, dextromethorphan, brompheniramine/dextromethorphan/pseudoephedrine, chlorpheniramine/dextromethorphan/phenylephrine, carbinoxamine/dextromethorphan/pseudoephedrine, dextromethorphan/promethazine, 1-aminocyclopropane-1-carboxylic acid |
| Q01097 | GRIN2B | glutamate receptor, ionotropic, N-methyl D-aspartate 2B | Plasma Membrane | ion channel | diagnosis,unspecified application | dextromethorphan/morphine, neramexane, bicifadine, delucemine, nebostinel, besonprodil, UK-240455, tenocyclidine, dextromethorphan/quinidine, donepezil/memantine, ketamine, felbamate, ifenprodil, memantine, orphenadrine, cycloserine, N-(2-indanyl)glycinamide, dextromethorphan, brompheniramine/dextromethorphan/pseudoephedrine, chlorpheniramine/dextromethorphan/phenylephrine, carbinoxamine/dextromethorphan/pseudoephedrine, dextromethorphan/promethazine, 1-aminocyclopropane-1-carboxylic acid |
| Q01098 | GRIN2C | glutamate receptor, ionotropic, N-methyl D-aspartate 2C | Plasma Membrane | ion channel |  | dextromethorphan/morphine, neramexane, bicifadine, delucemine, nebostinel, besonprodil, UK-240455, tenocyclidine, dextromethorphan/quinidine, ketamine, felbamate, memantine, orphenadrine, cycloserine, N-(2-indanyl)glycinamide, dextromethorphan, brompheniramine/dextromethorphan/pseudoephedrine, chlorpheniramine/dextromethorphan/phenylephrine, carbinoxamine/dextromethorphan/pseudoephedrine, dextromethorphan/promethazine, 1-aminocyclopropane-1-carboxylic acid |
| Q03391 | GRIN2D | glutamate receptor, ionotropic, N-methyl D-aspartate 2D | Plasma Membrane | ion channel |  | dextromethorphan/morphine, neramexane, bicifadine, delucemine, nebostinel, besonprodil, UK-240455, dextromethorphan/quinidine, ketamine, felbamate, memantine, orphenadrine, cycloserine, aspirin/caffeine/orphenadrine, N-(2-indanyl)glycinamide, dextromethorphan, brompheniramine/dextromethorphan/pseudoephedrine, chlorpheniramine/dextromethorphan/phenylephrine, carbinoxamine/dextromethorphan/pseudoephedrine, dextromethorphan/promethazine, 1-aminocyclopropane-1-carboxylic acid |
| Q925T6 | GRIP1 | glutamate receptor interacting protein 1 | Plasma Membrane | transcription regulator |  |  |
| P97772 | GRM1 | glutamate receptor, metabotropic 1 | Plasma Membrane | G-protein coupled receptor | efficacy | fasoracetam |
| Q14BI2 | GRM2 | glutamate receptor, metabotropic 2 | Plasma Membrane | G-protein coupled receptor |  | talaglumetad, fasoracetam |
| Q9QYS2 | GRM3 | glutamate receptor, metabotropic 3 | Plasma Membrane | G-protein coupled receptor |  | fasoracetam |
| Q68EF4 | GRM4 | glutamate receptor, metabotropic 4 | Plasma Membrane | G-protein coupled receptor |  | fasoracetam |
| Q3UVX5 | GRM5 | glutamate receptor, metabotropic 5 | Plasma Membrane | G-protein coupled receptor |  | fasoracetam, acamprosate |
| Q68ED2 | GRM7 | glutamate receptor, metabotropic 7 | Plasma Membrane | G-protein coupled receptor |  | fasoracetam |
| P47743 | GRM8 | glutamate receptor, metabotropic 8 | Plasma Membrane | G-protein coupled receptor |  | fasoracetam |
