# Supplementary material for: In-depth mapping of the mouse brain N-glycoproteome reveals widespread N-glycosylation of diverse brain proteins
Source: Oncotarget. 2016 May 31;7(25):38796–809. doi: 10.18632/oncotarget.9737 (PMC5122430; doi:10.18632/oncotarget.9737)
Supplement: Supplementary file 10 [file oncotarget-07-38796-s010.docx]

| Supplementary Table S11: N-glycosylated proteins in amyloid processing | | | | | | | |
| --- | --- | --- | --- | --- | --- | --- | --- |
| **Symbol** | **Synonym(s)** | **Entrez Gene Name** | **GenPept/UniProt/Swiss-Prot Accession** | **Location** | **Type(s)** | **Biomarker Application(s)** | **Drug(s)** |
| AKT3 | AI851531,D930002M15Rik,MPPH,MPPH2,Nmf350,PKB-GAMMA,PKB-γ,PKBG,PRKBG,Protein kinase Akt-3,RAC-gamma,RAC-PK-gamma,RAC-PK-γ,RAC-γ,STK-2,thymoma viral proto-oncogene 3,v-akt murine thymoma viral oncogene homolog 3 | v-akt murine thymoma viral oncogene homolog 3 | Q9WUA6 | Cytoplasm | kinase | efficacy | enzastaurin |
| APP | AAA,Abetapp,ABPP,AD1,Adap,Ag,amyloid beta (A4) precursor protein,Amyloid precursor,amyloid precursor protein,amyloid β (A4) precursor,amyloid β (A4) precursor protein,amyloid β A4 precursor,amyloid β precursor,amyloid β-protein precursor,amyloid-beta-protein,amyloid-β-protein,Amyloidogenic glycoprotein,APP isoform 1,APPI,appican,beta amyloid precursor protein,betaApp,CTFgamma,CVAP,E030013M08RIK,P3,PN-II,PN2,PreA4,protease nexin2,β amyloid precursor protein,β APP,β PP,β-amyloid precursor | amyloid beta (A4) precursor protein | P12023 | Plasma Membrane | other | diagnosis, efficacy, unspecified application | bapineuzumab, florbetapir F18, florbetaben F |
| BACE1 | ASP2,BACE,Beta-secretase,beta-site APP-cleaving enzyme 1,C76936,HSPC104,MEMAPSIN 2,β-secretase,β-site APP-cleaving enzyme 1 | beta-site APP-cleaving enzyme 1 | P56818 | Cytoplasm | peptidase |  | MK-8931 |
| CAPN1 | CALCIUM ACTIVATED NEUTRAL PROTEASE,calpain 1,calpain 1, (mu/I) large subunit,CALPAIN I,CANP,CANP1,CANPL1,Capa-1,MU-CALPAIN,mu-calpin,muCANP,muCL | calpain 1, (mu/I) large subunit | O35350 | Cytoplasm | peptidase |  |  |
| CAPN3 | AI323605,Calpain-3,CANP3,CANPL3,Capa-3,LGMD2,LGMD2A,Lp82,Lp84,Lp85,M calpain,nCL-1,p94 | calpain 3 | Q64691 | Cytoplasm | peptidase |  |  |
| CAPN11 | calpain 11,protease | calpain 11 | Q6J756 | Cytoplasm | peptidase |  |  |
| CAPNS1 | CALCIUM-DEPENDENT PROTEASE, SMALL subunit,CALPAIN 30 KDA REGULATORY subunit,Calpain Regulatory Subunit,Calpain Small Subunit 1,calpain small subunit 1-like,calpain, small subunit 1,CALPAIN4,CANP,CANPS,Capa-4,CAPN4,CDPS,Cpns1,CSS1,D7Ertd146e,LOC100911363,LOC100912380 | calpain, small subunit 1 | O88456 | Cytoplasm | peptidase |  |  |
| CSNK1A1 | 2610208K14Rik,4632404G05Rik,5430427P18RIK,casein kinase 1, alpha 1,casein kinase 1, α 1,Casein kinase-1, α-1 polypeptide,CASEIN KINASE1 alpha,CASEIN KINASE1 α,CK1,CK1a,CKI-alpha,CKI-α,CKIa,Csnk1a,HEL-S-77p,HLCDGP1,PRO2975 | casein kinase 1, alpha 1 | Q8BK63 | Cytoplasm | kinase |  |  |
| CSNK1D | 1200006A05Rik,AA409348,ASPS,casein kinase 1, delta,Ck1 delta,CK1δ,CKIdelta,D930010H05RIK,FASPS2,HCKID | casein kinase 1, delta | Q9DC28 | Cytoplasm | kinase |  |  |
| CSNK2A1 | casein kinase 2, alpha 1 polypeptide,casein kinase 2, α 1 polypeptide,Casein kinase II alpha 1 polypeptide,Casein kinase II α 1 polypeptide,Ck II Alpha Subunit,Ck II α Subunit,CK1 alpha2,Ck2 alpha,Ck2 α,CK2A1,CKII,CKII alpha,CKII α,Csnk2a1-rs4,CSNK2A3 | casein kinase 2, alpha 1 polypeptide | Q60737 | Nucleus | kinase |  |  |
| GSK3B | 7330414F15Rik,8430431H08Rik,C86142,glycogen synthase kinase 3 beta,glycogen synthase kinase 3 β,GSK-3,GSK-3beta,GSK-3β,GSKbeta,GSKβ,Tpk1 | glycogen synthase kinase 3 beta | Q9WV60 | Nucleus | kinase | efficacy | enzastaurin |
| MAPK3 | ERK-1,ERT2,Esrk1,HS44KDAP,HUMKER1A,Mapk p44,MAPK1,Mapkapk3,mitogen-activated protein kinase 3,MNK1,MTAP2K,p44,p44 Erk,P44ERK1,P44MAPK,PRKM3 | mitogen-activated protein kinase 3 | Q63844 | Cytoplasm | kinase | efficacy | ulixertinib |
| MAPT | AI413597,ALZ50,AW045860,DDPAC,FTDP-17,MAPTL,microtubule-associated protein tau,MSTD,Mtapt,MTBT1,MTBT2,PPND,PPP1R103,pTau,RNPTAU,TAU,Tau 3r,Tau-1,TAU-FACTOR,TAU4R | microtubule-associated protein tau | P10637 | Plasma Membrane | other | diagnosis, efficacy, unspecified application |  |
| NCSTN | 9430068N19Rik,AA727311,APH2,ATAG1874,D1Dau13e,Kiaa0253,mKIAA0253,NCT,NICASTRIN | nicastrin | P57716 | Plasma Membrane | peptidase |  |  |
| PRKAG1 | AA571379,Ampk gamma,AMPK gamma1,Ampk γ,Ampk γ 1,AMPKG,BB036179,Prkaac,Prkga1,protein kinase, AMP-activated, gamma 1 non-catalytic subunit,protein kinase, AMP-activated, γ 1 non-catalytic subunit | protein kinase, AMP-activated, gamma 1 non-catalytic subunit | O54950 | Nucleus | kinase |  |  |
| PRKAR2A | 1110061A24Rik,AI317181,AI836829,cAK RII α,Pka rII α,Pkarsa,PKR2,PRKAR2,protein kinase, cAMP dependent regulatory, type II alpha,protein kinase, cAMP dependent regulatory, type II α,protein kinase, cAMP-dependent, regulatory, type II, alpha,protein kinase, cAMP-dependent, regulatory, type II, α,RII alpha,RII α,RII(alpha),RII(α) | protein kinase, cAMP-dependent, regulatory, type II, alpha | P12367 | Cytoplasm | kinase |  |  |
